# Identification of Transient Intermediates and Active Species in Atomic CZA Catalysts for CO_2_ Hydrogenation to Methanol

**DOI:** 10.1021/jacs.5c08043

**Published:** 2025-11-11

**Authors:** Leonardo da Silva Sousa, Andrea Bertuzzi, Tanna Elyn Rodrigues Fiuza, Edson Roberto Leite, Patricia Benito, Davide Ferri, Daniela Zanchet, Andrew M. Beale

**Affiliations:** † Instituto de Química, 344102 Universidade Estadual de Campinas, Cidade Universitária Zeferino Vaz, Campinas, São Paulo 13083-970, Brazil; ‡ Department of Chemistry, University College London, London WC1H 0AJ, U.K.; § Research Complex at Harwell, Rutherford Appleton Laboratory, Harwell Science and Innovation Campus, Didcot OX11 0FA, U.K.; ∥ Department of Industrial Chemistry, 9296 Università di Bologna, Viale Risorgimento 4, Bologna 40136, Italy; ⊥ Brazilian National Nanotechnology Laboratory (LNNano), CNPEM, Campinas, São Paulo 13083-970, Brazil; # Paul Scherrer Institute, 28498 PSI Center for Energy and Environmental Sciences, Villigen CH-5232, Switzerland; 7 Chemistry Department, Federal University of São Carlos (UFSCar), São Carlos, São Paulo 13565-905, Brazil

## Abstract

Direct hydrogenation of carbon dioxide to methanol is a promising strategy for carbon capture and utilization (CCU). Copper–zinc–alumina (CZA) catalysts are widely used for this transformation, yet the nature of the active Cu and Zn species and the reaction intermediates remains debated due to their sensitivity to feed composition and temperature. This challenge is compounded by the high metal loading in conventional CZA catalysts, which obscures active species signals with background contributions from spectator species. To address this, we synthesized model CuZn/Al_2_O_3_ catalysts using bimetallic coordination complexes, achieving low metal loadings that yield small Cu clusters and Cu^+^ single atoms adjacent to isolated Zn^2+^ sites. In situ XANES and UV–vis data were analyzed using multivariate curve resolution–alternating least-squares (MCR–ALS), revealing that Cu dispersion and reagglomeration—phenomena suspected in industrial systems—also occur at low loadings. Operando and modulation excitation with phase sensitive detection DRIFTS (ME-PSD-DRIFTS) showed: (a) Cu clusters dissociate H_2_ and activate CO_2_ via monodentate formate; (b) Al_2_O_3_ stabilizes Cu^+^ under reducing conditions, with Cu content correlating with methanol productivity via CO hydrogenation; and (c) Zn in ZnAl_2_O_4_ promotes CO_2_ activation through reactive carbonate formation and enhances oxygenate conversion kinetics. ZnAl_2_O_4_ also acts as a structural promoter, facilitating CO_2_ conversion via reverse water gas shift (RWGS) and CO hydrogenation. These findings reveal new structure–activity relationships, highlighting the role of the mixed-metal interface in stabilizing transient intermediates and providing some guidance in the rational design of improved catalysts for CO_2_ valorization.

## Graphical abstract

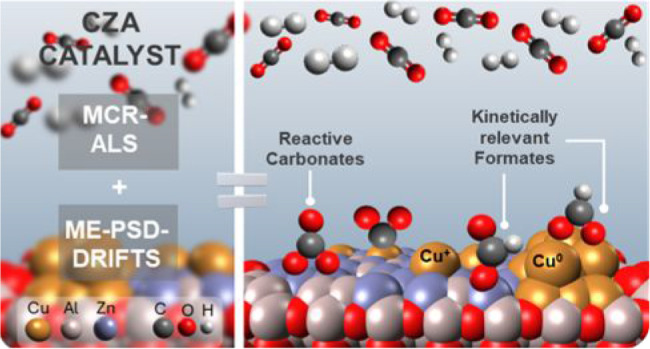

## 1 Introduction

The catalytic conversion of CO_2_ offers a promising strategy to mitigate rising atmospheric CO_2_ levels, while simultaneously enabling the production of valuable fuels and chemical feedstocks needed for a carbon-neutral future.^1^ Beyond providing alternatives to fossil resources, CO_2_ utilization is critical for closing the carbon cycle, decarbonizing hard-to-electrify sectors such as aviation and shipping, and supporting the transition toward sustainable chemical manufacturing. Recent analyses have highlighted that CO_2_-derived fuels and chemicals could play a pivotal role in sectors where renewable electricity alone is insufficient, offering pathways for deep emission reductions while enhancing energy security and resource circularity.^2,3^ The scientific challenges lie in the high thermodynamic stability of CO_2_, a low-energy, highly stable molecule, and its kinetic inertness, as breaking the carbon–oxygen double bonds requires substantial energy.^1–3^ CO_2_ hydrogenation to methanol is particularly compelling when combined with hydrogen from renewable sources, as methanol serves as a versatile chemical, clean fuel, and liquid hydrogen carrier, making it integral to decarbonizing the energy and chemical sectors.

After decades of research, an economically viable catalyst for methanol synthesis from syngas (a mixture of CO, CO_2_, and H_2_) was developed by Imperial Chemical Industries (ICI) in the late 1960s. It is composed of three components: Cu/ZnO/Al_2_O_3_, typically with concentrations of 60 wt % Cu, 30 wt % ZnO, and 10 wt % Al_2_O_3_.^4^ This catalyst, named CZA, has the advantage of operating under mild conditions (220–300 °C, 50–100 bar), however, the reactional feed is majorly composed of CO and H_2_, with smaller contributions of CO_2_. In fact, direct CO_2_ hydrogenation (CO_2_ + 3H_2_ ⇌ CH_3_OH + H_2_O, ΔH_r_^298^ = −41.2 kJ mol^–1^), i.e., in the absence of CO, is thermodynamically more challenging, leading to significant conversion and selectivity drops.^5,6^ From here onward, we will refer to syngas hydrogenation as methanol synthesis, and the direct CO_2_ hydrogenation, i.e., a CO_2_-rich stream without CO, will be referred to as CO_2_ hydrogenation.

The transition toward an optimized catalyst for CO_2_-rich feed has been based on the knowledge built over the years for CZA catalysts and their adaptations. Industrial formulation and derivations have been studied for over a century, and a thorough review written by Bokhoven and coworkers^7^ has covered this extensive history, exploring how the understanding of this material has evolved over the past decades up to the present day. However, despite this wealth of knowledge, the debates and controversies raised over the years only serve to establish that CZA and methanol synthesis do not operate in a singular and well-defined manner. Instead, they function across a wide range of conditions, involving multiple active sites and reaction pathways. Although extensive studies have advanced the understanding of structural characteristics and catalytic behavior, critical gaps remain regarding the dynamic evolution of Cu and Zn species under reaction conditions and how these evolving species correlate with intermediate formation and mechanistic pathways. Traditional characterization approaches often fail to capture the transient, reactive states of the catalyst, leaving fundamental questions unresolved: What is the nature of the Cu and Zn species under working conditions? How do these species interact with reactants to promote specific reaction intermediates? And how does their evolution influence the overall mechanism of methanol production? Addressing these questions requires techniques capable of probing catalysts under realistic conditions with sufficient temporal and chemical resolution to unravel the complex structure–activity relationships governing CO_2_ hydrogenation.

It has been shown that copper alone is active in methanol synthesis.^8,9^ Surface science studies have established that CO_2_ conversion occurs on a bare copper surface, with catalytic activity being directly dependent on particle size and morphology—i.e., a typical case of structure sensitivity, often showing that the reactivity trend follows the order Cu(111) < Cu(100) < Cu(110).^10–13^ From a theoretical perspective, Higham et al.^14^ helped elucidate the reactivity differences using density functional theory (DFT) calculations of CO_2_ conversion to methanol over the less dominant Cu(110) and Cu(100) surfaces. Not only did this corroborate the structure sensitivity, but it also demonstrated that these two surfaces enabled different pathways from the dominant Cu(111), explaining their higher activity. In the context of traditional CZA catalyst, Behrens et al.^15^ studied the industrial catalyst Cu/ZnO/Al_2_O_3_ using X-ray photoemission spectroscopy (XPS), aberration-corrected transmission electron microscopy (AC-TEM), neutron diffraction, and performed DFT calculations to support their experimental data. The catalytic activity model proposed by the authors demonstrated the importance of defects, showing that undistorted Cu is relatively inactive. In contrast, high activity was achieved with a Cu surface enriched in steps, which can be stabilized by bulk defects, such as stacking faults or twin boundaries. Another crucial factor was the presence of Zn^δ+^ on the defective Cu surface.

Similar to Cu, ZnO alone can act as an active material for CO_2_ activation and subsequent hydrogenation to methanol.^16^ ZnO has been attributed to various roles, from controlling copper particle size distribution to promoting strong metal–support interaction (SMSI) and even forming Cu–Zn alloys. In the SMSI context, a nonstoichiometric ZnO_*x*_ phase evolves around the Cu nanoparticles, increasing the number of Cu/Zn interfacial sites and helping stabilize Cu^+^ sites, which are active for methanol synthesis.^17^ Additionally, the Cu/ZnO interaction enhances Cu nanoparticle dispersion, resulting in smaller nanoparticles and a higher metallic surface area.^17,18^

Regarding the debate over alloyed versus oxidic zinc, a key study was conducted by Kattel et al.^19^ The authors investigated a model catalyst, ZnCu(111), to simulate the ZnCu alloy, ZnO/Cu(111), Cu/ZnO(0001̅), and ZnO/Cu/ZnO (to simulate Cu/ZnO interfaces) and used XPS, DFT, and kinetic Monte Carlo (KMC) simulations. The authors demonstrated that under reaction conditions (CO_2_:H_2_ 1:9, 250–300 °C), ZnCu undergoes oxidation, with Zn segregating from the alloy to form ZnO, which is coupled with an enhancement in catalytic activity. Experiments with ZnO/Cu(111) clarified the origin of the increased activity, which the authors associated with the facilitation of key elementary steps. Overall, this work provides strong evidence that the more active site in Cu/Zn catalysts is the interfacial site between Cu and ZnO. From these past studies, the following questions arise: is it necessary for Zn to be specifically in the ZnO phase? Is CuZn alloying essential? Do these aspects serve only to improve an already active system further?

The third component, which is often overlooked, is Al_2_O_3_. Long believed to be merely a structural promoter, alumina has been shown to be less inert than previously thought, participating in material reconstruction under reaction conditions and even playing a role in the reaction mechanism. From a compositional perspective, Behrens et al.^20^ reported a positive impact of Al^3+^ cations being inserted into the ZnO lattice, acting as a promoter when present in ideal concentrations. The authors hypothesized that this effect could be related to the promotion of defects in the ZnO phase and improved reducibility. Conversely, Zn can be incorporated into the Al_2_O_3_ lattice to form ZnAl_2_O_4_. A long-duration (150 days) stability study by Lunkenbein et al.^21^ described two deactivation phases in CZA. The first, relatively fast, was mainly associated with transformations in the ZnO phase, in which alumina reacted with ZnO to form zinc aluminate. In contrast, the second one was slower and related to the sintering of both Cu and Zn phases. It is worth noting that Al_2_O_3_ is not inert to CO_2_; it can bind CO_2_ and stabilize intermediates or spectators, such as formate and methanol.^22–24^

Herein, we aim to understand the direct CO_2_ hydrogenation to methanol and what is behind copper and zinc synergism. For that, we synthesized model catalysts that resulted in both copper clusters or single atoms in the vicinity of isolated Zn^2+^ sites on the Al_2_O_3_ surface. Our goal was to understand model copper sites (small clusters and single atoms) and determine whether the mere presence of Zn^2+^ sites was sufficient to promote CO_2_ hydrogenation to methanol, avoiding the influence of different ZnO phases, vacancy formation, SMSI, and alloying processes. To achieve these goals, we synthesized materials with low metal loading (1 wt % on Al_2_O_3_) to avoid extensive sintering. Additionally, to ensure spatial proximity between the bimetallic CuZn pair, we synthesized a metallic precursor based on organic ligands with two coordination sites to simultaneously bind Cu and Zn. Thus, both metals were impregnated simultaneously and, most importantly, in close spatial proximity from the early stages of catalyst preparation. We studied the stability of the materials *in situ* and demonstrated that Cu was highly dynamic within the cluster size range. Even under inert atmosphere, the metallic clusters redisperse during cooling down steps. Once exposed to the reaction atmosphere, the metallic phase was recovered, showing that the reaction atmosphere played a major role in the actual active sites during the catalytic tests, highlighting the need for caution during the analysis of ex situ data.

Subsequently, through a series of *in situ* and *operando* measurements combined with multivariate curve resolution alternating least-squares (MCR-ALS) analysis and diffuse reflectance infrared Fourier transform spectroscopy (DRIFTS), including modulation excitation phase-sensitive detection DRIFTS (ME-PSD-DRIFTS), we gained unprecedented insights into structure–activity relationships in this important catalytic system. In particular, we found that Cu clusters not only facilitated H_2_ dissociation but also provided sites for CO_2_ activation via Cu-bound formate species. We further observed a correlation between the presence of isolated Cu^+^ species and methanol yield, with Al_2_O_3_ stabilizing Cu^+^ ions even under highly reducing conditions. Meanwhile, Zn was incorporated into the Al_2_O_3_ surface, forming ZnAl_2_O_4_, with no evidence of CuZn alloy formation, indicating that at low concentrations, Zn preferentially reacted with the support rather than with Cu nanoparticles. Operando ME-PSD-DRIFTS demonstrated that the ZnAl_2_O_4_ phase promoted CO_2_ hydrogenation by offering an alternative route for CO_2_ activation through the formation of reactive carbonates and by accelerating the conversion of monodentate formate species. Additionally, Zn exerted an indirect promotional effect by stabilizing Cu^+^ sites. Overall, we demonstrated that copper played a crucial role in both CO_2_ and H_2_ activation, with formate bound in the monodentate configuration on the support identified as a key intermediate, while a CO hydrogenation mechanism operating through Cu^+^ sites significantly contributed to methanol production.

## 2 Experimental Section

### 2.1 Materials

All reagents were used as received without further purification. *o-*Vanillin (99%), ethylenediamine (≥99%), copper­(II) acetate monohydrate (Cu­(OAc)_2_·H_2_O, 97%), zinc­(II) nitrate hexahydrate (Zn­(NO_3_)_2_·6H_2_O, 98%), gamma aluminum oxide nanopowder (γ-Al_2_O_3_, 50 nm), anhydrous ethanol (EtOH, 99.5%), and acetonitrile (>99%) were purchased from Sigma-Aldrich.

### 2.2 Complex Synthesis and Characterization

The synthesis of the complexes consisted of two steps: ligand preparation and subsequent coordination with metallic centers. The synthesis was adapted from a previously reported method;^25^ the general procedure is shown in Scheme 1. Briefly, *o-*vanillin was condensed with ethylenediamine under ethanol reflux, and the valen ligand was isolated, which was later coordinated with Cu­(OAc)_2_. The Cu­(valen) complex was isolated by precipitation. For the bimetallic complex, Zn­(NO_3_)_2_ was added to a solution of Cu­(valen) in MeCN:EtOH 7:3 at 75 °C to yield the CuZn­(valen) complex, which could be isolated by precipitation. The complete synthesis and characterization are provided in the Supporting Information.

**Scheme 1:**
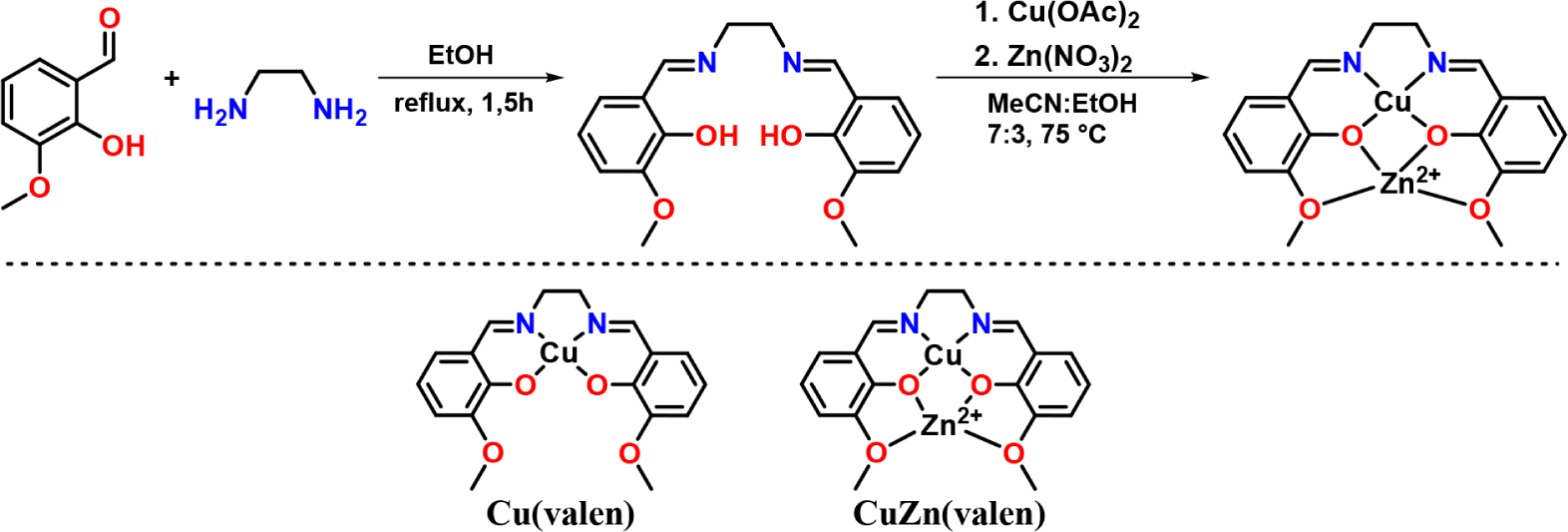
General Procedure for the Synthesis of Metallic Precursors Starts with Ligand Synthesis and is Followed by Coordination with Cu or Cu and Zn

### 2.3 Catalyst Preparation

The catalysts were prepared via wet impregnation. In the case of the 1 wt % Cu/Al_2_O_3_ preparation, in a 100 mL round-bottom flask, 60 mL of ethanol was added, and heated to 75 °C, then Cu­(valen) complex (31.2 mg, 0.08 mmol) was added, once fully dissolved, 495 g of Al_2_O_3_ was added. The material was agitated and heated until completely dry. For the 1 wt % CuZn/Al_2_O_3_ impregnation, the bimetallic complexes CuZn­(valen) was freshly prepared, and instead of being isolated, 495 mg of Al_2_O_3_ would be added to the solution, then the systems were kept under agitation and heating until completely dry. All impregnated materials were calcined in a tubular furnace under 100 mL·min^–1^ synthetic air flow (20% O_2_ in 80% N_2_) at 500 °C, a heating rate of 10 °C·min^–1,^ and a dwell time of 1 h. The materials were named Cu/Al_2_O_3__C500Rx or CuZn/Al_2_O_3__C500Rx, where C indicates calcination and R indicates reduction at x = 200 or 400 °C.

### 2.4 Characterization

Thermogravimetric analysis (TGA) was performed using a TA Instruments TGA Q600 instrument at a heating rate of 20 °C·min^–1^ to 1000 °C under a flow of 100 mL·min^–1^ of either synthetic air or nitrogen.

Pretreatment: Unless otherwise stated, all *in situ* calcination experiments were done using 20% O_2_ in Ar at a total flow of 50 mL·min^–1^, with a heating rate of 10 °C·min^–1^ and a 30 min dwell time at 500 °C. The reduction involved 30% H_2_ in Ar at a total flow of 50 mL/min, with a heating rate of 10 °C·min^–1^ and a 30 min dwell time at 200 or 400 °C.

*In situ* Diffuse Reflectance UV–vis (DR-UV–vis) was performed using a Linkam CCR1000 cell with an OceanOptics high-resolution spectrometer. Spectra were collected in reflectance mode from 200 to 1030 nm, averaging 20 scans with a 700 ms integration time each. Typically, 20 mg of material was loaded into a ceramic sample holder. During calcination, a mass spectrometer (MS) was connected to the cell exhaust to monitor the combustion products. Data was processed using SpectraGryph software, converting reflectance units into Kubelka–Munk units.

*In situ* X-ray Absorption Fine Structure (XAFS) experiments were conducted at the B18 Quick-EXAFS beamline at Diamond Light Source, UK. The setup included a bending magnet source, Si(111) double crystal monochromator, toroidal mirrors for beam collimation, and plane mirrors for harmonic rejection. The samples were loaded into quartz capillaries with 3 mm outer diameter, 100 mm length, and 0.025 mm wall thickness, and mounted in a capillary furnace. Gas flow was maintained below 20 ml·min^–1^, using 6 ml·min^–1^ of H_2_ and 14 ml·min^–1^ of He (30% H_2_/He mixture) during the reduction step. The heating ramp was 10 °C·min^–1^ up to 400 °C, with a 15 min dwell time. Sample spectra were collected in fluorescence mode using a 36-element Ge detector, with short scans (8779–9259 eV) at the Cu K-edge (emission set to Cu Kα) and Zn short scans (9459–9959 eV) at K-edge (emission set to Zn Kα) during heating, and long scans for Cu K-edge (8779–9959 eV) and Zn K-edge (9459–10639 eV) at the initial state (room temperature) and 400 °C. Reference foils of Cu and Zn were scanned simultaneously for continuous energy calibration in the transmission mode. The ion chambers were filled with He/N_2_/Ar mixtures optimized for the beam energy. Calibration was performed using the first inflection point of metallic Cu and Zn foils. Reference spectra for the oxides were prepared as pellets using commercial standards (Cu_2_O, CuO, ZnO, ZnAl_2_O_4_) and measured ex situ in transmission mode under ambient conditions at the B18 beamline. Data processing was done using the Demeter software^26^ package, including polynomial fitting, background subtraction, and normalization. The Fourier Transform (FT) was applied in the k-range of 3 to 11.5 Å^–1^, using a k-weighting of 2.

MCR-ALS analysis for *in situ* XAFS and DR-UV–vis data was performed using MCR-ALS GUI 2.0, a MATLAB-based package reported by Tauler and coworkers.^27^ Prior to MCR-ALS analysis, Principal Component Analysis (PCA) was performed using Singular Value Decomposition (SVD) to determine the appropriate number of components for the spectral data matrices. The number of components was selected based on the inflection point (“knee”) in the scree plot of singular values, corresponding to the point where additional components contribute minimally to the explained variance. This ensured that the selected number of components captured the chemically relevant information while minimizing contributions from experimental noise. The initial estimation was done by Evolving Factor Analysis (EFA). For the MCR analysis, non-negativity and unimodality constraints for all components were applied using the fast non-negative least-squares (FNNLS) algorithm with 10% tolerance. The spectra were normalized using the Euclidean norm.

*Quasi in situ* XPS was performed using a Thermo Fisher K-Alpha X-ray Photoelectron Spectrometer, which features an Al Kα microfocused monochromator as the X-ray source and a 180° double-focusing hemispherical analyzer. The pass energy was maintained at 50 eV throughout the measurements. Samples were reduced *ex situ* in a fixed-bed reactor in a tubular furnace under 15 mL·min^–1^ of H_2_ and 35 mL·min^–1^ of Ar (30% H_2_), 10 °C·min^–1^, at 200 or 400 °C. To avoid exposure to air, the reactor was sealed with gas still flowing, and the flow ceased immediately after to avoid pressure buildup. The reactor was then opened inside a glovebox, and the samples were mounted on the XPS sample holder, which was sealed under vacuum in the antechamber of the glovebox. Data processing was performed using CASA software. A linear background was subtracted from the raw data and the binding energy was calibrated to adventitious carbon C 1s at 284.8 eV.

*Operando* DRIFTS was performed using a Bruker VERTEX 80 system with a high-pressure dome (up to 25 bar) and a DiffusIR accessory from PIKE Technologies. A 1:3 CO_2_:H_2_ mixture was supplied using a Bronkhorst EL-FLOW mass-flow controller. The entire setup has been reported previously in detail by our group.^28^ Spectra were collected in absorption mode, averaging 32 scans in the 4000 to 500 cm^–1^ range with a 4 cm^–1^ resolution. Background spectra were obtained by replacing the sample with a mirror and averaging 64 scans. Typically, 20 mg of material was reduced *in situ*, and the samples were then cooled to room temperature, switched to the reaction mixture at 30 mL·min^–1^, heated to 250 °C at 5 °C·min^–1^, and pressurized to 20 at 1 bar·min^–1^. Reference spectra for methanol and formate were collected by injecting 10 μL of methanol or formic acid in 30 mL·min^–1^ Ar flow with the sample heated to 130 °C.

ME-PSD-DRIFTS experiments were performed using a Bruker VERTEX 70v spectrometer, the cell and modulation system used here were previously described in detail, including the gas flow paths and residence time studies.^29^ To achieve fast acquisition, spectra were collected in absorption mode with a resolution of 4 cm^–1^, averaging 10 scans, scanner velocity of 80 kHz, LN-MCT detector, and aperture of 8 mm. The reaction was carried out at 250 °C and atmospheric pressure, and the feed was modulated by three solenoidal valves, one constantly feeding 15 mL·min^–1^ H_2_ and 5 mL·min^–1^ Ar, the second 5 mL·min^–1^ CO_2_, and the third 5 mL·min^–1^ Ar, resulting in 25 mL·min^–1^ total flow, and CO_2_:H_2_ 1:3 ratio. After reaching steady state (approximately 1 h), the second valve was turned off for 60 s, whereas the third valve was turned on to maintain a constant total flow and H_2_ concentration. After another half cycle of 60 s, the two valves were switched, and the modulation was repeated over 50 cycles of 2 min each. The modulation period of 120 s (60 s half-cycle, 8 mHz) was selected to match the modulation frequency to the estimated turnover frequency (TOF) of the CO_2_ hydrogenation reaction under our conditions (∼3 mHz). The data were processed using homemade software; typically, the initial and last five cycles were disregarded, averaging over 40 cycles. The periodic component of the spectral intensity at each wavenumber was calculated using phase-sensitive detection, as described in ref^30^, using eq 1:

1 $$I\left(\varphi^{PSD}\right)=\frac{2}{T}\int_{0}^{T}I\left(t\right)\sin\left(k\omega t+\varphi^{PSD}\right)dt$$

where I­(*φ* ^PSD^) is the PSD data signal (absorbance) intensity, T is the modulation period, I­(t) the intensity at time t, k is the demodulation index and was set equal to 1 for every analysis, t is the time, and φ^PSD^ is the phase angle.

*In situ* CO–DRIFTS was conducted to probe the superficial Cu sites on the reduced and used catalysts using a Bruker VERTEX 80 spectrometer and a PIKE cell. Sample spectra were collected in absorption mode, averaging 32 scans in the 4000 to 500 cm^–1^ range with a 4 cm^–1^ resolution. For the background spectrum, a mirror was used, and 64 scans were averaged. After *in situ* treatment, materials were cooled to room temperature and exposed to 10% CO in He at 10 mL·min^–1^ until spectral changes ceased. The flow was then switched to Ar at 10 mL·min^–1^ to remove excess CO and desorb physically adsorbed species, and the spectra were collected until no further changes were observed. Temperature-programmed desorption (TPD) experiments were done in a Harrick cell, after CO chemisorption and desorption steps, the sample was heated in 25 °C steps under Ar 10 mL·min^–1^ holding for 10 min at each temperature, and the temperature was raised until all chemisorbed species were desorbed.

Scanning transmission electron microscopy (STEM) was done using a spherical aberration-corrected Titan Cubed Themis (Thermo Fisher Scientific) microscope at LNNano/CNPEM (Proposal 20243636), operated at 300 kV. The CuZn_C500R400 before and after reaction samples were dispersed in isopropyl alcohol and dripped on Au grids coated with carbon membrane, and the images were collected using a high-angle annular dark field (HAADF) detector.

### 2.5 Catalytic Tests

For the CO_2_ hydrogenation to methanol, the calcined catalysts were pelletized, crushed, and sieved through 30–40 mesh (0.595–0.400 mm). Subsequently, 500 mg of the sieved material was mixed with SiC (30–40 mesh) to achieve a final volume of 1.2 cm^3^. The samples were loaded into a tubular reactor (INCOLOY 800HT) with an internal diameter of 10 mm. The gas flow rates were regulated using mass flow controllers (Brooks Instruments), and the pressure was controlled using a back-pressure regulator (Swagelok). All lines after the reactor outlet were heated to 200 °C to prevent the condensation of methanol and water. Prior to testing, the catalysts were reduced *in situ* using 50 mL·min^–1^ of 30% H_2_ in N_2_, a heating rate of 10 °C·min^–1^, and held at 200 °C (or 400 °C) for 1 h. CO_2_ hydrogenation to methanol was carried out using a feed mixture with a CO_2_:H_2_ ratio of 1:3 and a total flow of 140 mL·min^–1^. Catalytic tests were conducted at 30 bar with a GHSV value of 7000 mL·V_cat_^–1^·h^–1^, where V_cat_ is the final volume of the catalytic bed in cm^3^. In a typical test, the reactor was purged with N_2_ until it reached 225 °C, then pressurized to the working pressure with the reaction mixture. Catalyst activity was monitored at temperatures ranging from 225 to 300 °C with steps of 25 °C and isothermals of 3 h at each temperature. The reaction stream was analyzed using an online gas chromatograph (Agilent Technology 7890A) equipped with two thermal conductivity detectors (TCD) and a CarboPLOT P7 column for CH_4_, CO, CO_2_, and CH_3_OH analyses using H_2_, and an HP-Molesieve column for H_2_ detection using N_2_. Data are presented as Space-Time Yield (STY) by taking the ratio between methanol flow (g_MeOH_·h^–1^) and the catalyst mass (g_cat_).

## 3 Results and Discussion

### 3.1 Precursor Impact on Cu and Zn Speciation

Working with two metals even at a low loading of 1 wt % can pose the challenge of heterogeneous distribution on the support surface during impregnation steps, hindering the proximity between the two sites. To overcome this challenge, we opted to use bimetallic coordination compounds, in which both metal centers were impregnated simultaneously and, most importantly, with proximity in space, enhancing the chances of these metallic centers sticking close to one another after thermal treatment. The precursors can be seen in Scheme 1; they are Schiff bases, commonly referred to as “salen-like” ligands. Initially, Cu binds to the first coordination shell, which is composed of two nitrogen and two oxygen binding sites. Then, for the bimetallic precursor, Zn^2+^ is inserted into the second coordination shell, which is also tetradentate and composed of four oxygen binding sites. We have successfully employed such a class of precursors in the synthesis of monometallic Pt/TiO_2_ systems by designing the ligand structure to tailor their thermal stability, consequently influencing the thermal decomposition steps.^31^ Other studies have also successfully reported the use of similar bimetallic systems.^32,33^

To understand the decomposition profiles of the precursors and speciation of Cu during thermal treatment, we employed *in situ* DR-UV–vis spectroscopy. Figure 1a shows the results of calcination of the alumina-supported CuZn­(valen) complex (CuZn/Al_2_O_3_), with similar data for Cu/Al_2_O_3_ presented in Figure S1. At room temperature, the spectrum consisted of four bands characteristic of the complex: the bands at 240 and 281 nm were associated with the π–π* transitions of the aromatic ligand, the band at 370 nm arose from ligand-to-metal charge transfer (LMCT), and the band at 569 nm was due to the d–d transition of the Cu^2+^ center in its square planar geometry. As the temperature increased to approximately 250 °C, the bands broadened, and the d–d band blue-shifted. When the temperature reached 300 °C, the d–d band disappeared, leaving only π–π* bands. Finally, at 500 °C, a new d–d band appeared at 725 nm, which was associated with copper coordinated to oxygen atoms (Cu^2+^-O^2–^) on the Al_2_O_3_ surface.

**Figure 1:**
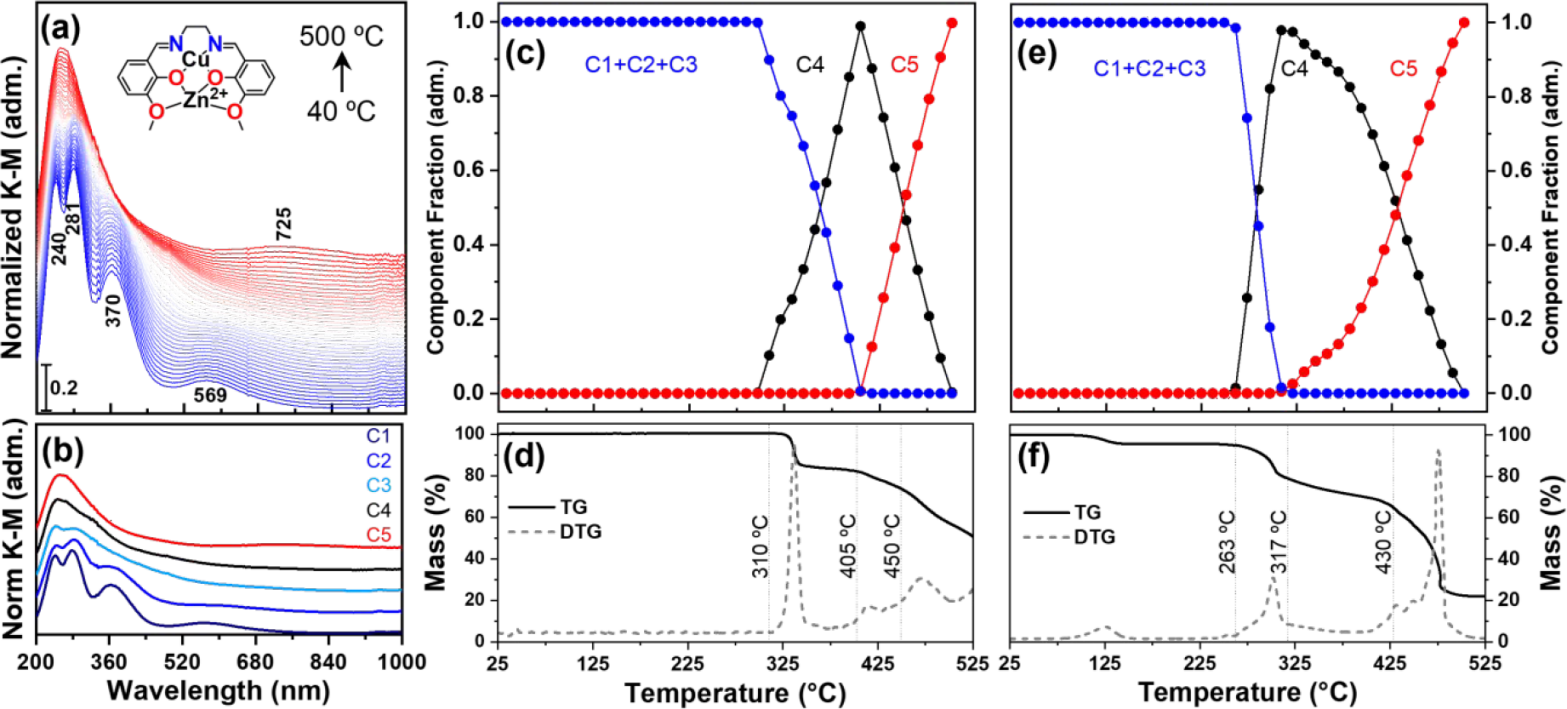
(a) *In situ* DR-UV–vis calcination of CuZn/Al_2_O_3_; (b) MCR-ALS components extracted from (a); (c) concentration profile extracted by MCR-ALS of CuZn/Al_2_O_3_; (d) TGA CuZn­(valen); (e) concentration profile extracted by MCR-ALS of Cu/Al_2_O_3_; and (f) TGA of Cu­(valen).

To gain a better understanding of the evolution of the species involved in calcination, we resorted to MCR-ALS analysis. MCR is a powerful chemometric tool that allows the extraction of chemically meaningful components from *in situ* data and the relative quantification of these species without the need for standards, which are commonly used for traditional linear combination fitting (LCF) methods.^34^ Figure 1b presents the five components extracted from the MCR analysis: component C1 is identical to the initial complex spectrum, while components C2 and C3 are similar to C1, apart from some broadening and small position shifts, which can be explained by thermal effects; overall, the three components most likely represent the same chemical species, that is, the initial CuZn­(valen) complex. Component C4 differed significantly from the previous ones and only had π–π* transitions, which could indicate the presence of residual aromatic carbon species on the surface as intermediaries in the calcination process. Finally, component C5 depicts the new Cu­(II) environment, with a broad LMCT band centered at 255 nm associated with O^2–^ ligands and a d–d transition band at 725 nm. The results of calcination of the monometallic complex Cu/Al_2_O_3_ are summarized in Figure S1. A similar trend to that of the bimetallic counterpart was observed, in which the complex decomposition went through an intermediary organic residue buildup on the surface, followed by its consumption and Cu^2+^ center formation on the alumina surface.

Considering that the three components C1, C2, and C3 extracted from the MCR analysis describe the same species, we can narrow down to the evolution of the three intermediates, and their concentration profiles as a function of temperature can be seen in Figure 1c for CuZn/Al_2_O_3_. The complex remained stable up to 310 °C, after which its concentration decreased sharply, followed by an increase in the C4 component. When the temperature reached 405 °C, (C1 + C2 + C3) decreased to zero, whereas C4 reached its highest value. TGA data (Figure 1d) showed that in this temperature range, only a partial weight loss of 16.5% occurred, indicating that most of the organic mass remained on the surface, which is consistent with the formation of component C4 associated with aromatic residues. At 405 °C, component C5, associated with Cu^2+^, started to evolve. From the TGA analysis, the weight slowly decreased until 450 °C, after which major changes occurred, with a 47.2% mass loss above this temperature. From the mass spectrometry results (Figure S2a), there was a large evolution of CO, CO_2_, and H_2_O at this stage, which was a consequence of the complete combustion of the residual carbon phase. From the MCR concentration profile, this was the region where C4 decreased rapidly, accompanied by C5 increment. The results for the monometallic precursor Cu/Al_2_O_3_ are shown in Figure 1e,f. This precursor had outcomes similar to those of the monometallic precursor; however, the presence of the second metal (Zn) affected the overall thermal stability of the complex, which shifted the decomposition process to higher temperatures. In the Cu/Al_2_O_3_ case, the decomposition started earlier at 265 °C, and the formation of Cu^2+^ (C5) took place at 320 °C. Another aspect is the stability of the C4 residual phase, as indicated by the MCR-ALS and TGA analyses, which occurred during a larger temperature interval.

To understand the speciation and morphological aspects of the Cu^2+^ and Zn^2+^ species formed after calcination, we analyzed the samples using *ex situ* XAFS spectroscopy, as shown in Figure 2 (Figure S5 for k-space plotting). The X-ray absorption near edge structure (XANES) and extended X-ray absorption fine structure (EXAFS) results for the calcined samples CuZn/Al_2_O_3__C500 and Cu/Al_2_O_3__C500 at the Cu K-edge are summarized in Figure 2a. The results showed that, independent of the precursor, the final Cu^2+^ chemical environment on the alumina surface was similar for all materials. In the XANES region, the pre-edge feature at 8977.5 eV corresponds to the dipole-forbidden 1s–3d transition, which is typical for the Cu^2+^ electronic state. The edge region helps to understand the structural aspects in more detail, and the samples show a rather featureless edge, similar to the Cu­(OH)_2_ reference. It corresponds to a more symmetrical environment, indicating that Cu^2+^ is most likely in an octahedral environment, being coordinated by O^2–^ ligands from the surface, and −OH and H_2_O from the atmosphere. From the Fourier Transform (FT) of the EXAFS, the three materials presented a similar first coordination shell, which matched the Cu­(OH)_2_ reference, with no clear higher shells, indicating that copper was in a highly dispersed state. The quantitative analysis of the EXAFS data is shown in Table S1.

**Figure 2:**
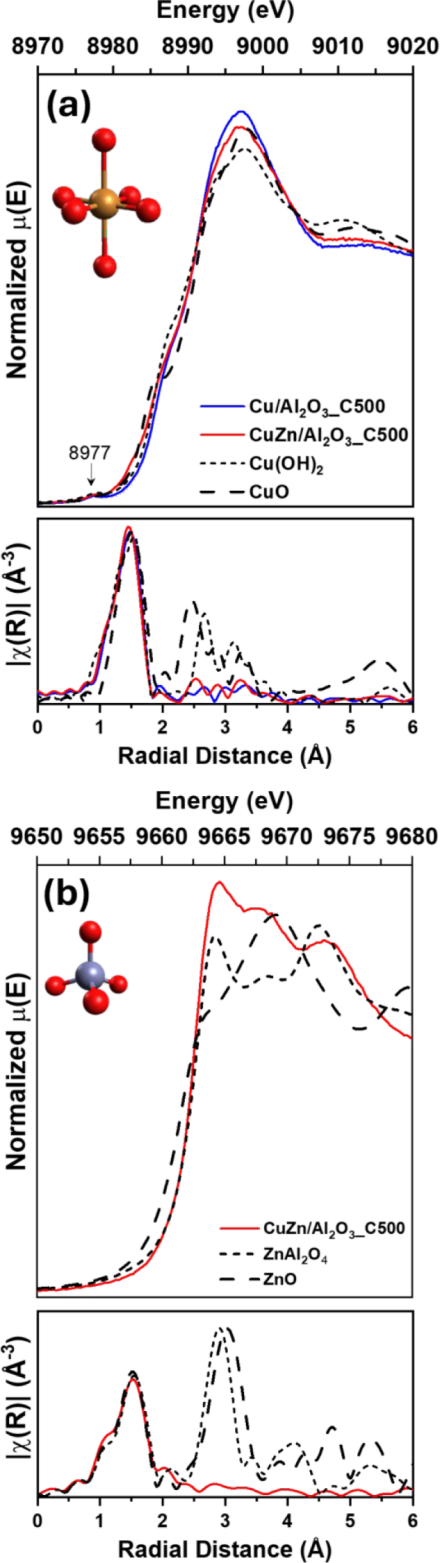
XANES and EXAFS spectra: (a) Cu K-edge for Cu/Al_2_O_3__C500 and CuZn/Al_2_O_3__C500; (b) Zn K-edge for CuZn/Al_2_O_3__C500. References (CuO, Cu­(OH)_2_, ZnO, and ZnAl_2_O_4_) are shown for comparison. Typical k range: 3–11 Å.

The XANES and EXAFS analysis at the Zn K-edge for CuZn/Al_2_O_3__C500 are shown in Figure 2b. In the XANES region, Zn presented a distinct profile from the references, but of all the references, it most closely matched ZnAl_2_O_4_; the higher intensity observed at 9668 eV points at Zn^2+^ in a closer environment to Zn in ZnAl_2_O_4_, but not as ordered as it would be in a crystalline material. The deviation from the bulk ZnAl_2_O_4_ XANES profile is attributed to possible contributions of ZnO, and it is also consistent with literature reports, where structural disorder and oxygen vacancies lead to significant spectral variations despite maintaining tetrahedral Zn^2+^ coordination.^35,36^ From the EXAFS data, the first coordination shell matched a tetrahedral environment of Zn, as found in both the ZnAl_2_O_4_ and ZnO (hexagonal phase) references. No clear higher shell contribution was observed, indicating the presence of a highly dispersed Zn species. The quantification of Cu and Zn species on the surface was done by X-ray photoelectron spectroscopy (XPS) and all values closely matched the expected theoretical numbers, the complete discussion can be found in Supporting Information Figures S3 and S4, also Tables S2 and S3. Overall, the bimetallic and monometallic precursors had similar decomposition mechanisms during calcination; the main differences were in the thermal stability of the species, which increased in the presence of Zn, influencing the Cu^2+^ formation temperature. All the precursors resulted in the same Cu^2+^ configuration, which was a distorted octahedron that was highly dispersed on the alumina surface. Zn was incorporated into the alumina lattice and remained on the surface.

### 3.2 Cu Speciation during Activation and Reaction Conditions. The Dynamic Nature of Cu Clusters and Single Atoms and the Impact of Zn

#### 3.2.1 Effect of the Promoter on Cu Reduction

To clarify the effects of reduction on the active sites, we studied the two catalysts using *in situ* XANES and DR-UV–vis spectroscopy. Both techniques are sensitive to the electronic structure of Cu, with XANES probing core to valence and unbound state transitions and UV–vis describing the valence electronic transitions for molecular species or plasmon resonance for metallic clusters.

The evolution of the Cu K-edge from room temperature to 400 °C for Cu/Al_2_O_3__C500 is illustrated in Figure 3a, where the white line (WL) shows a clear change from Cu^2+^ to Cu^0^. A closer inspection of the edge region showed the presence of not only one, but two shoulders. The first one at 8980 eV corresponds to metallic copper, while the second, at 8983 eV, relates to the Cu^+^ 1s–4p transition. Nevertheless, the position of this second feature is blue-shifted from 8982 eV of the Cu_2_O reference, suggesting a slightly different Cu^+^ species of the linearly bicoordinated Cu_2_O phase. The XANES results showed that even under harsh reducing conditions, a minority of the oxidic-copper species remained. Figure 3b shows the *in situ* reduction monitored by DR-UV–vis spectroscopy. The initial Cu^2+^ state (blue line) was consumed, and after reduction, two new bands appeared at 580 and 360 nm, which were attributed to the surface plasmon bands of metallic copper clusters.^50–52^

**Figure 3:**
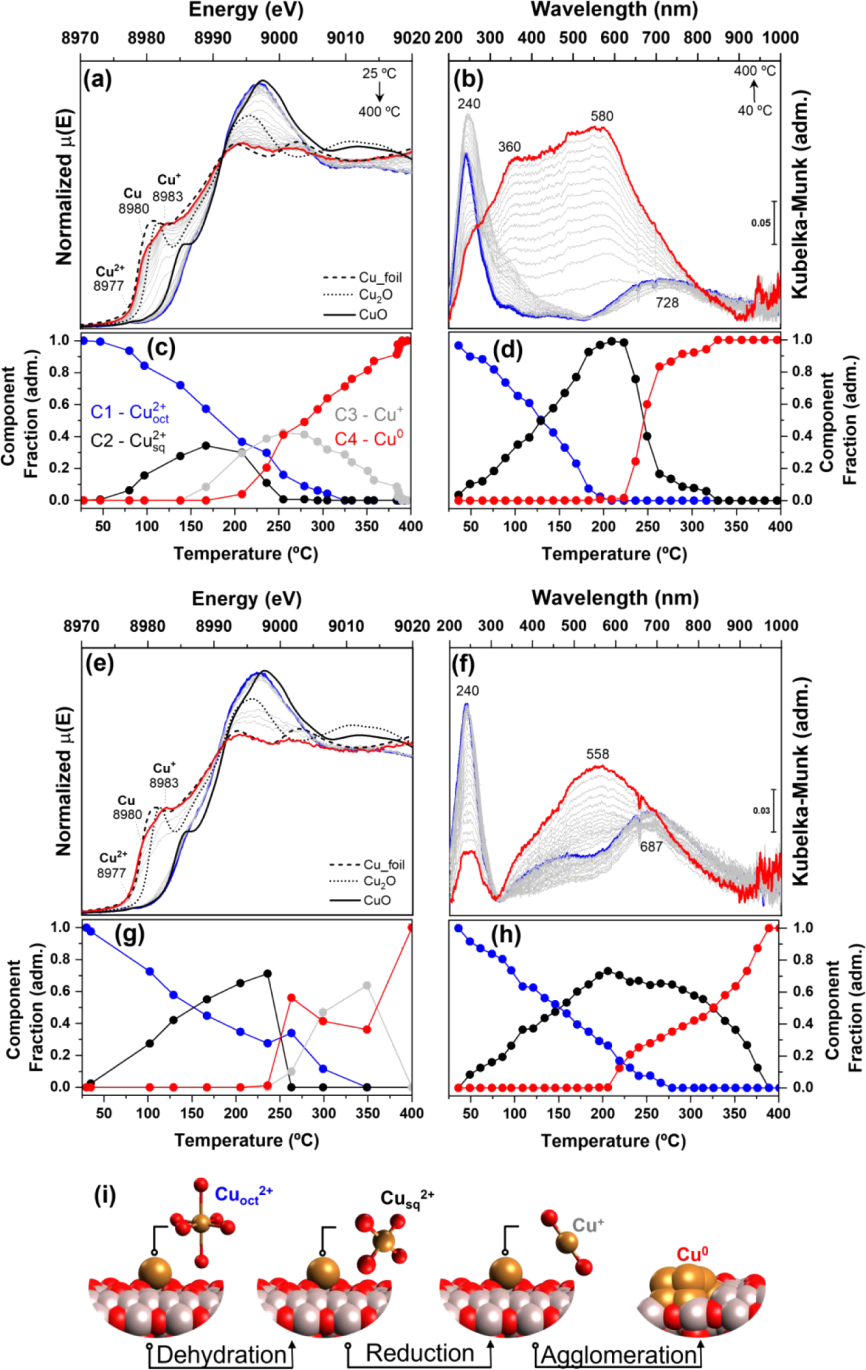
(a) *In situ* XANES of the Cu K-edge and (b) *in situ* DR-UV–vis spectra of Cu/Al_2_O_3__C500; (c) and (d) MCR-ALS components extracted from (a–b). (e) *In situ* XANES of the Cu K-edge and (f) *in situ* DR-UV–vis spectra of CuZn/Al_2_O_3__C500; (g) and (h) MCR-ALS components extracted from (e–f). (i) Schematic representation of Cu species evolution. The blue line corresponds to the initial state at room temperature, and the red line to the final state at 400 °C. Experimental conditions: 30% H_2_ in inert gas, 50 mL·min^–1^ (DR-UV–vis) and 20 mL·min^–1^ (XANES), 10 °C·min^–1^, from room temperature to 400 °C.

We further analyzed both sets of experiments using MCR-ALS to provide a better description of species evolution as a function of temperature. *In situ* XANES analysis revealed four components describing the reduction process (Figure S6a). C1 corresponds to hydrated octahedral Cu^2+^ (Cu^2+^_oct_),^37,38^ and C2 corresponds to dehydrated Cu^2+^ as the CuO reference,^37–39^ which has a square planar structure resulting from octahedral dehydration (Cu^2+^_sq_). The C3 ^39,40^ component showed a profile similar to that of Cu^+^ in the Cu_2_O phase, except for the 1s–4p transition at a higher energy of 8983 eV, which can be attributed to 2- to 3-coordinated Cu­(I) sites, as shown by systematic investigations of Cu model complexes, in the literature.^41–45^ where the 2- and 3-coordinate Cu­(I) complexes, edge absorption features appear in the 8982–8985 eV range, attributed to 1s → 4p transitions. In contrast, for 4-coordinate tetrahedral Cu­(I) complexes, 1s → 4p transitions were observed at energies exceeding 8985 eV. It is also important to highlight that when comparing the component C3 to the Cu_2_O reference spectrum (Figure S7), there are solid evidence for a highly dispersed Cu^+^ moieties, as further discussed in the SI and at the following sections. The final component, C4, closely matched the metallic Cu reference, although the absorption at 8983 eV suggested the presence of Cu^+^, indicating that this component was not purely metallic Cu.

The MCR-ALS analysis of the *in situ* DR-UV–vis data resulted in only three components (Figure S6b). The first component, C1^uv^, corresponds to the initial hydrated distorted octahedral Cu^2+^.^46–48^ The second component, C2^uv^, shows a broadening of the LMCT band and a red shift of the d-d transition to 752 nm, which are typical characteristics of dehydrated Cu^2+^, that is, square planar Cu­(II), such as CuO.^46–49^ The final component, C3^uv^, corresponds to the surface plasmon resonance of metallic Cu^0^. No component for Cu^+^ was detected because Cu^+^, being a d^10^ system, does not exhibit d-d transitions/no noticeable MLCT bands. Figure 3c,d show the evolution of the MCR components for Cu/Al_2_O_3__C500 as a function of temperature based on the XANES and DR-UV–vis data, respectively. As shown in Figure 3c, the catalyst began to dehydrate as early as 80 °C and continued to dehydrate up to 175 °C, the temperature at which the C2 component peaked and the C3 component (Cu^+^) began to emerge, reaching its maximum at 250 °C, and the C4 component (Cu^0^) began to make significant contributions after 225 °C. The UV–vis results were consistent with the XANES data, indicating that dehydration was the primary process up to approximately 200 °C. However, UV–vis spectroscopy showed complete conversion from hydrated to dehydrated states, likely due to the differences in the furnaces used for the two techniques: a capillary furnace for XANES and a Linkam cell for DR-UV–vis. As shown in Figure 3d, UV–vis also supported the metallic particles formation, with the C3^uv^ component (Cu^0^) beginning to appear at 225 °C. Despite the limitations of DR-UV–vis in detecting Cu^+^, it proved to be an effective technique for describing the transitions between oxidized and reduced states.

Figure 3e shows the results observed for CuZn/Al_2_O_3__C500 during the *in situ* XANES Cu K-edge analysis; overall, it presented similar transitions to those of Cu/Al_2_O_3__C500, with the final state comprising a majority Cu^0^ particles and residual Cu^+^ species. However, in the *in situ* DR-UV–vis analysis of CuZn/Al_2_O_3__C500 (Figure 3f), the resulting surface plasmon band was dominated by a single band centered at 558 nm. The typical plasmon band for copper nanoparticles (10 nm) has been reported to be centered at 570 nm.^50^ A detailed study conducted by Vázquez-Vázquez et al. explored the growth of copper particles using UV–vis spectroscopy and showed that the plasmon changes as a function of particle size, with small clusters presenting bands at 295 nm, once grown into larger clusters red shifting to 360 nm (0.8 nm) and 390 nm (1.3 nm), followed by larger clusters at 460 nm (1.8 nm), and finally reaching nanoparticles with plasmonic bands at 560 nm (>2 nm).^51,52^ Considering these attributions, the presence of bands at 360 and 580 nm for Cu/Al_2_O_3__C500R400 indicates the presence of small and larger clusters, while the dominant band at 558 nm for CuZn/Al_2_O_3__C500R400 implies larger clusters as the major component. It is worth mentioning that in our case, it is not possible to infer the exact particle size, as our materials present a supported copper moiety, which is correlated to a different dielectric environment than those discussed previously in the literature, but the correlation between band position and particle size is expected to hold true.

The MCR-ALS components for the CuZn/Al_2_O_3__C500 catalysts can be seen in the Supporting Information (Figure S6c,d); the overall trends are similar to those of the monometallic Cu catalyst, with changes restricted to the band positions. The evolution of the MCR components as a function of temperature is shown in Figure 3g,h, while a schematic representation of the observed transitions for both catalysts can be found in Figure 3i. Both techniques indicated that the dehydrated components peaked at 225 °C. At this temperature, XANES analysis suggested the simultaneous formation of Cu^+^ and Cu^0^. However, because the Cu and Zn K-edges were acquired sequentially during the same experiment, the temporal resolution of the Cu K-edge was compromised, resulting in fewer data points. Despite this, the data indicate that from 250 to 350 °C, the highest contribution of Cu^+^ was observed, with a slower evolution of Cu^0^. DR-UV–vis spectroscopy, which had a higher temporal resolution, confirmed that oxidic Cu was more stable between 250 and 325 °C, and within this range, Cu^0^ formation was slower. This behavior contrasts with that of Cu/Al_2_O_3__C500, where Cu^+^ was formed and consumed earlier, leading to a faster reduction to Cu^0^. Our results demonstrated that the presence of Zn delayed the reduction, stabilizing Cu^+^ on the surface at temperatures as high as 350 °C.

To gain further insight into the morphological aspects, we collected EXAFS data at 400 °C under steady-state conditions (Figure S8), and the quantitative fitting results are summarized in Table S4. The Cu/Al_2_O_3__C500R400 and CuZn/Al_2_O_3__C500R400 samples produced similar Cu clusters, with Cu–Cu coordination numbers (CN Cu–Cu) of 5.6 and 5.9, respectively, which are statistically similar when error bars are considered. As previously reported, the estimated particle size can be inferred from the EXAFS coordination number.^53–57^ After accounting for the contribution of Cu^+^ species, the Cu–Cu coordination number was corrected from 5.9 to 7.9 (see details in the SI). Based on this corrected value and assuming a hemispherical particle geometry, the estimated particle size is approximately 1.8 nm. Also, both samples showed a Cu–Cu bond distance of 2.50 Å, slightly shorter than the 2.54 Å observed in the reference, another indicative of small particle formation, as the bond distance has been shown to decrease as particles get smaller.^56^ For all samples, a small shoulder at 1.54 Å (uncorrected for phase) was observed, which was attributed to the Cu–O bond. EXAFS fitting resulted in an average Cu–O bond length of 1.87 Å, in agreement with the typical Cu–O bond in the Cu_2_O reference, which is 1.85 Å. This observation supports the presence of Cu^+^ at high temperatures, which is consistent with the 1s–4p transition at 8983 eV observed in the XANES region.

#### 3.2.2 Zn Speciation during Reduction

We also monitored the changes in Zn using *in situ* XANES, as its speciation during reduction has been a topic of debate, there are claims to oxidic zinc been the active phase, whereas others argue that alloying between Cu and Zn occurs.^19^ Figure 4 shows the Zn K-edge reduction behavior of CuZn/Al_2_O_3__C500 from room temperature to 400 °C. The most significant changes were observed in the white line features at 9664 and 9669 eV, where a reduction in the intensity occurred at higher temperatures. These modifications were reversible after cooling back to room temperature, as evident in Figure S9, showing that the changes were mostly due to thermal effects. No evidence of alloying was observed in either the Zn or Cu results; we believe that under this diluted metallic regime, the competition was won by surface incorporation instead of alloying. The analysis also indicated that ZnAl_2_O_4_ is stable under reducing conditions and, once formed, is unlikely to be converted into other chemical states. The changes in zinc are in good agreement with previous reports that followed CZA stability over long periods, where zinc aluminate represented an energetic global minimum in their thermodynamic conditions.^21^

**Figure 4:**
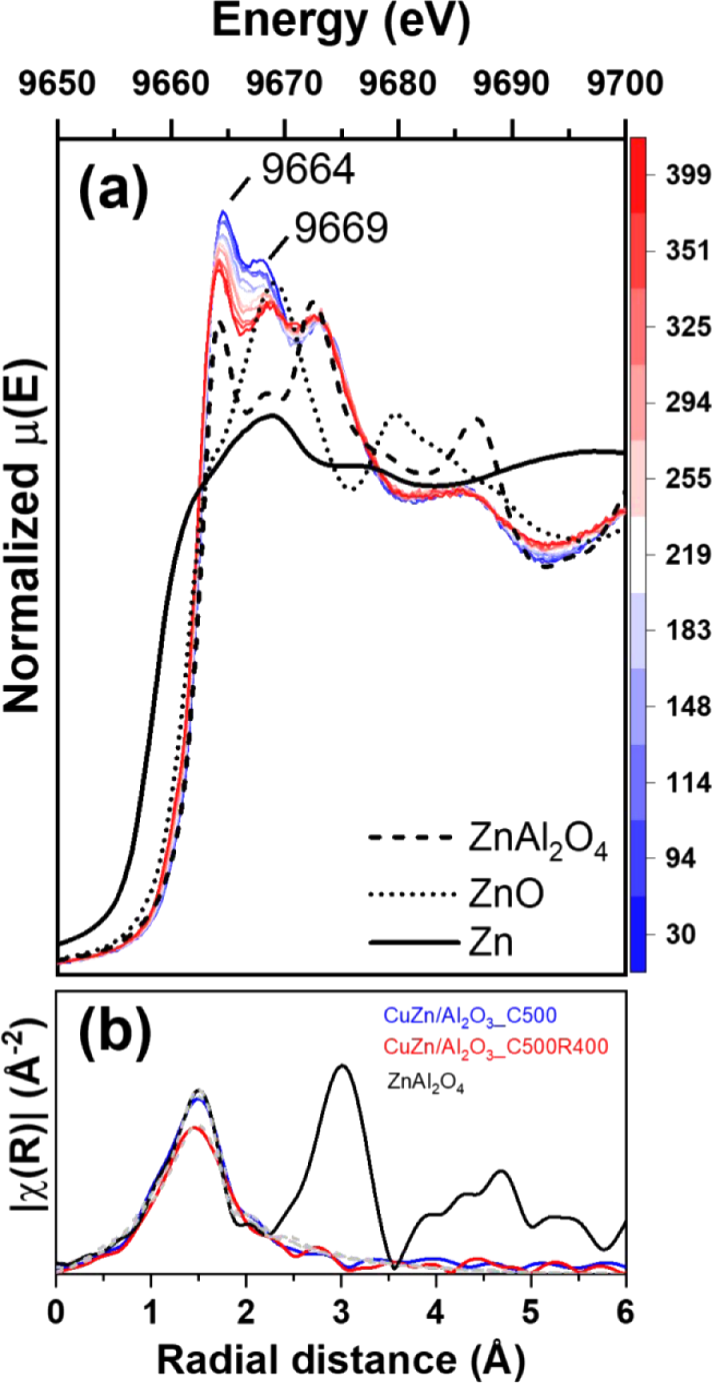
(a) *In situ* reduction XANES of the Zn K-edge for CuZn/Al_2_O_3__C500; (b) EXAFS spectra at Zn K-edge for CuZn/Al_2_O_3__C500 at room temperature and CuZn/Al_2_O_3__C500R400 measured at 400 °C. Experimental conditions: 30% H_2_ in inert gas, 50 mL·min^–1^, 10 °C·min^–1^, from 30 to 400 °C. References: Zn foil, ZnO, and ZnAl_2_O_4_. Typical k range: 3–11 Å.

From the qualitative analysis of the Zn EXAFS region in Figure 4b (Figure S10 for k-space plotting), the initially calcined CuZn/Al_2_O_3__C500 sample showed an intensity very close to that of the ZnAl_2_O_4_ standard, and after reduction, CuZn/Al_2_O_3__C500R400 exhibited a lower intensity. Quantitatively, the Zn–O coordination number (CN) was 3 for CuZn/Al_2_O_3__C500, which is lower than the expected value of 4 from the reference. This discrepancy could be explained by the higher Debye–Waller factor for the calcined sample (σ^2^ = 0.005) compared to that of the reference (σ^2^ = 0.002), which suggests that the dispersed Zn species is more disordered than its crystalline bulk counterpart. A closer inspection of the Zn–O bond length shows that both samples (calcined and reference) have a statistically similar bond length of 1.93 Å. The reduced sample, the coordination number decreased slightly to 2.6, with an increase in the Debye–Waller factor, and Zn–O bond length of 1.95 Å, which is similar to the reference. Both samples (calcined and reduced) were statistically similar, corroborating the stability of Zn in ZnAl_2_O_4_. Considering all the observations, we demonstrated that the presence of Zn modulates the reduction behavior of Cu/Al_2_O_3_; specifically, Zn delayed the complete reduction of Cu ions, leading to the stabilization of Cu^+^, which remained stable up to the maximum temperature used of 400 °C.

#### 3.2.3 Cluster Redispersion and Reagglomeration

The STEM HAADF images for CuZn/Al_2_O_3__C500R400 before (Figure 5a,b) and after the reaction (Figure 5c,d) show the presence of two morphologically distinct populations: clusters larger than 1 nm and single atoms lodged on the surface. To access the speciation of these sites, we resorted to CO–DRIFTS, as shown in Figure 5e,f. Typically, the CO stretching frequency for Cu^2+^ falls above 2140 cm^–1^, Cu^+^ in the 2140–2110 cm^–1^ range, and Cu^0^ below 2110 cm^–1^. It is also well established that CO weakly binds to Cu^2+^ and is completely reversible at room temperature, while it binds strongly to Cu^+^ and moderately to Cu^0^.^58^

**Figure 5:**
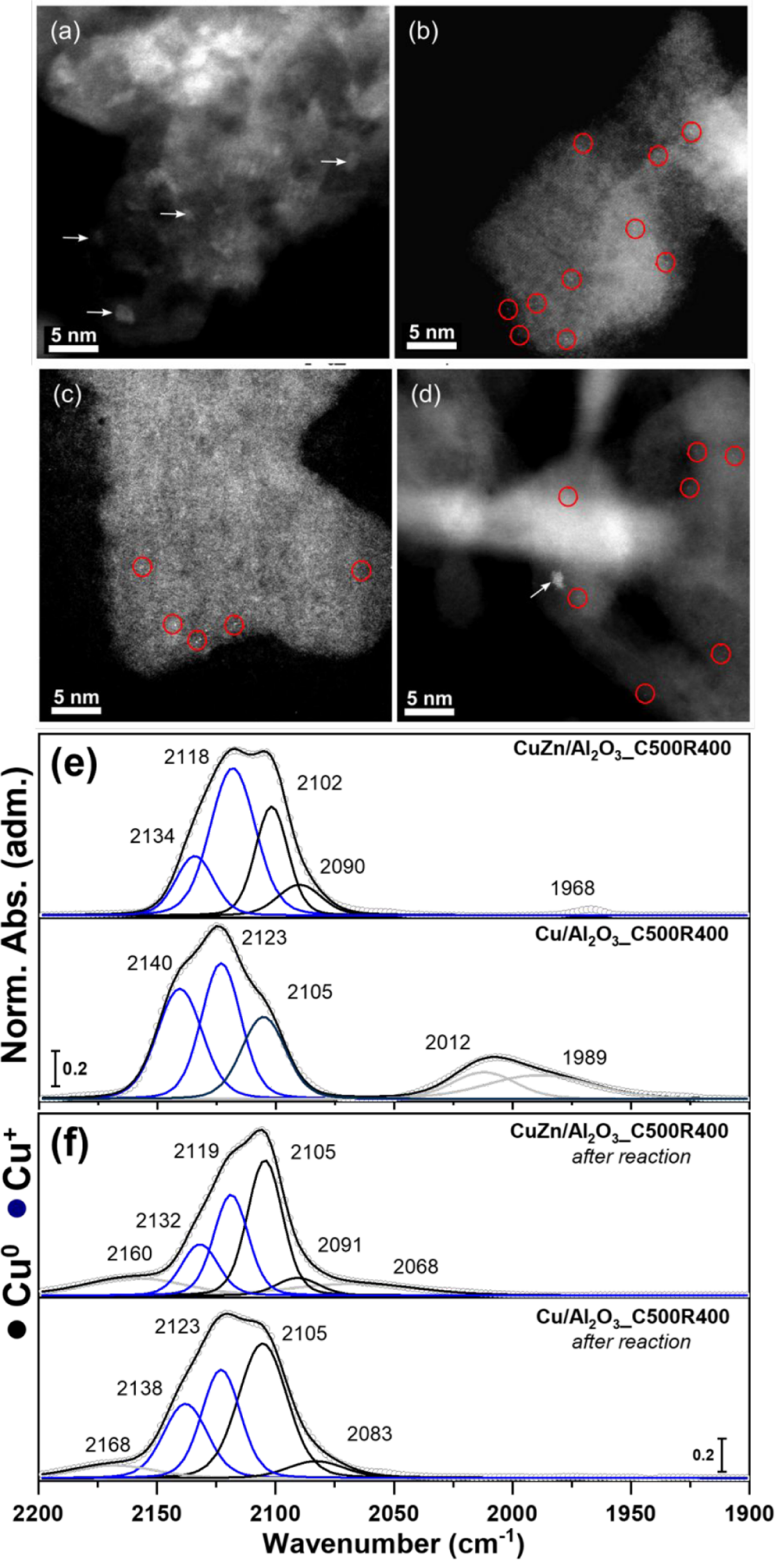
STEM HAADF images for CuZn/Al_2_O_3__C500R400 (a,b) before and (c,d) after the reaction, highlighting the presence of clusters (white arrows) and single atoms dispersed on the surface (red circle). Scale bar: 5 nm. *In situ* CO DRIFTS for CuZn/Al_2_O_3__C500R400 and Cu/Al_2_O_3__C500R400 (e) before and (f) after reaction (reduction at 400 °C).

The Cu and CuZn/Al_2_O_3__C500R400 samples after reduction (Figure 5e) and after the reaction (Figure 5f) had three major bands, 2105–2101 cm^–1^, 2123–2118 cm^–1^, and 2140–2134 cm^–1^. We attributed the bands at ∼2120 and 2134 cm^–1^ to Cu^+^ (two distinct sites;^59^ the former relates to typical Cu_2_O with Cu centered in a linear coordination environment and the latter to possible distortions of this site. The bands around 2100 cm^–1^ were attributed to high-index Cu^0^ sites. To further support these attributions, we performed TPD of these species, as shown in Figure S16, which demonstrated that the CO at 2100 cm^–1^ desorbed at considerably lower temperatures than those at 2120 and 2134 cm^–1^, consistent with previous assignments to the presence of weaker CO bonding on Cu^0^ and Cu^+^ species, respectively. In this context, we attributed the oxidic Cu^+^ bands to the isolated sites as single atoms, and the metallic Cu^0^ band to the superficial sites of the observed nanoparticles. All band positions and assignments are summarized in Table 1. Overall, the main difference between the reduced and used catalysts was the relative intensity between the observed species. After the reaction, there was an increase in the metallic population, possibly due to the formation of larger particles, which are more resistant to redispersion than smaller particles.^60–62^ The data set for materials reduced at 200 °C is summarized in Figure S17. Overall, the changes followed a similar trend to those reported for materials reduced at 400 °C.

**Table 1:** Band Assignments for *In Situ* CO–DRIFTS Experiments

Wavenumber (cm^–1^)	ASSIGNMENT	LITERATURE
2090	Cu(110)	2093,[Bibr ref71] 2092 ^72^
2102–2107	High index Cu^0^ surfaces	2100–2110, [Bibr ref58],[Bibr ref59],[Bibr ref73] 2090–2103,^74^ 2102–2104,^75^ 2110,[Bibr ref76] 2099–2104 ^71^
2118–2128	Cu^+^ in Cu_2_O structure	2125–2127,^69^ 2127 ^70^
2132–2134	Cu^+^	2132 ^71^

The *ex situ* observations suggest the presence of plentiful amounts of oxidized copper in the samples, in contrast to the *in situ* results studied during reduction at high temperatures, which showed a material mostly composed of metallic clusters (particles < 1 nm). The presence of Cu^+^ sites under the reaction conditions has been a highly debated topic in the literature. Studies that claim the existence of Cu^+^ often base their conclusions using *ex situ* data alone, raising concerns because copper nanoparticles can readily oxidize in the presence of oxygen and *ex situ* experiments frequently fail to account for this behavior.^63,64^ To obtain a complete description of the copper species dynamics, we followed the evolution of the Cu species after the *in situ* reduction during the cooling step, then the heating under reaction conditions, and cooling after reaction, with the *in situ* DR-UV–vis data summarized in Figure 6 for CuZn/Al_2_O_3__C500R400 and Figure S18 for Cu/Al_2_O_3__C500R400.

**Figure 6:**
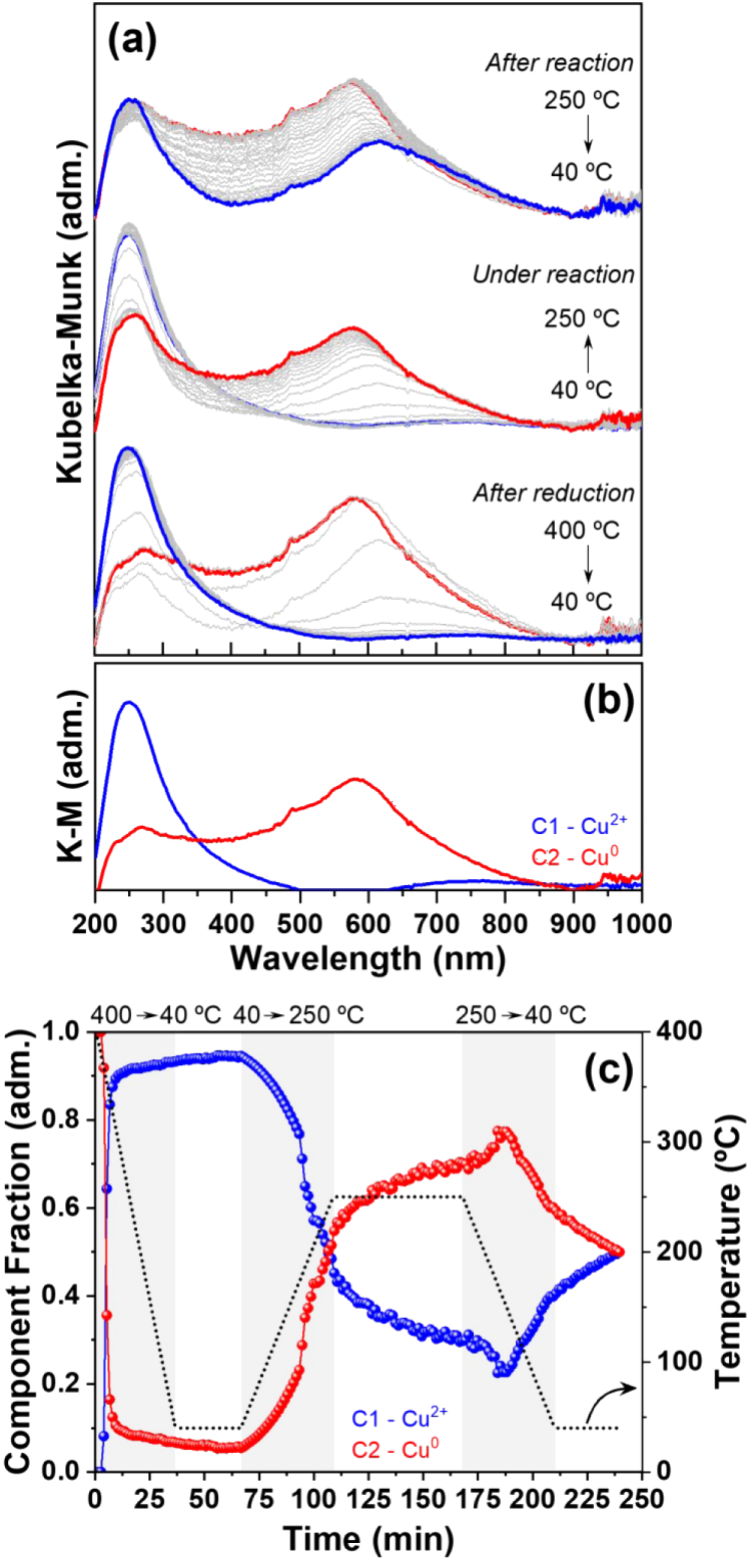
*In situ* DR-UV–vis during the cooling step after reduction, heating under reaction atmosphere, and cooling after reaction for (a) CuZn/Al_2_O_3__C500R400; (b) MCR-ALS components C1 (Cu^2+^) and C2 (Cu^0^); and (c) MCR-ALS temperature profile.

The cooling step after reduction for CuZn/Al_2_O_3__C500R400 is shown in the bottom part of Figure 6a, revealing the evolution from metallic Cu^0^ to oxidic Cu^2+^. Once the atmosphere was changed to CO_2_:H_2_ 1:3 and heated to 250 °C, the metallic clusters were restored, and after 1 h under reactional conditions, the system was cooled again, and partial oxidation was observed. These changes were followed by MCR-ALS and two components were extracted (Figure 6b): C1 corresponding to Cu^2+^ and C2 to Cu^0^. From the temperature profile (Figure 6c), as soon as the system started to cool, quick oxidation occurred, rapidly reaching the steady state. Once exposed to the reaction conditions (ambient pressure), the system was continuously reduced and kept reducing at a slower pace than that during the isothermal experiment. Finally, during the cooling step after reaction, slower oxidation occurred, with both chemical states coexisting by the end of it. The oxidation observed in both cooling steps could be explained by two mechanisms: the first as stable copper particles that undergo redox changes, or the second in which oxidation is associated with metallic particles redispersion into oxidized Cu^+^/Cu^2+^ single sites. Based on *ex situ* TEM and CO–DRIFTS observations, we believe that redispersion is the most plausible mechanism. It has also been reported that the tendency to undergo redox changes or redispersion is size-dependent, with the latter occurring more readily for smaller particles.

Considering the limitations of DR-UV–vis in the observation of Cu^+^ (d^10^), we studied the same dynamics using XANES, and the combination of both techniques helped provide a better understanding of Cu^+^/Cu^2+^ evolution. During the cooling step after reduction (inert atmosphere), the XANES data for CuZn/Al_2_O_3__C500R400 (Figure 7a) and Cu/Al_2_O_3__C500R400 (Figure 7b) clearly indicate the conversion of metallic Cu^0^ to Cu^+^. When the atmosphere was switched to a CO_2_:H_2_ (1:3) mixture and heated to 250 °C (ambient pressure), the redispersed sites reagglomerated into the metallic state while maintaining the feature at 8983 eV. By the end of the reaction, during the cooling phase, the clusters were redispersed into Cu^+^ sites. The changes observed for the Zn sites during *in situ* XANES are shown in Supporting Information Figure S9. Zn^2+^ in ZnAl_2_O_4_ environment was stable throughout all steps, with no indication of coordination change, only variations in the white line intensity, consistent with previous observations. In such dilute catalysts (1 wt %), the Zn could have migrated to form a CuZn alloy, agglomerated into ZnO_*x*_, or incorporated into the alumina lattice and it is this latter step that occurs, particularly at the alumina surface.

**Figure 7:**
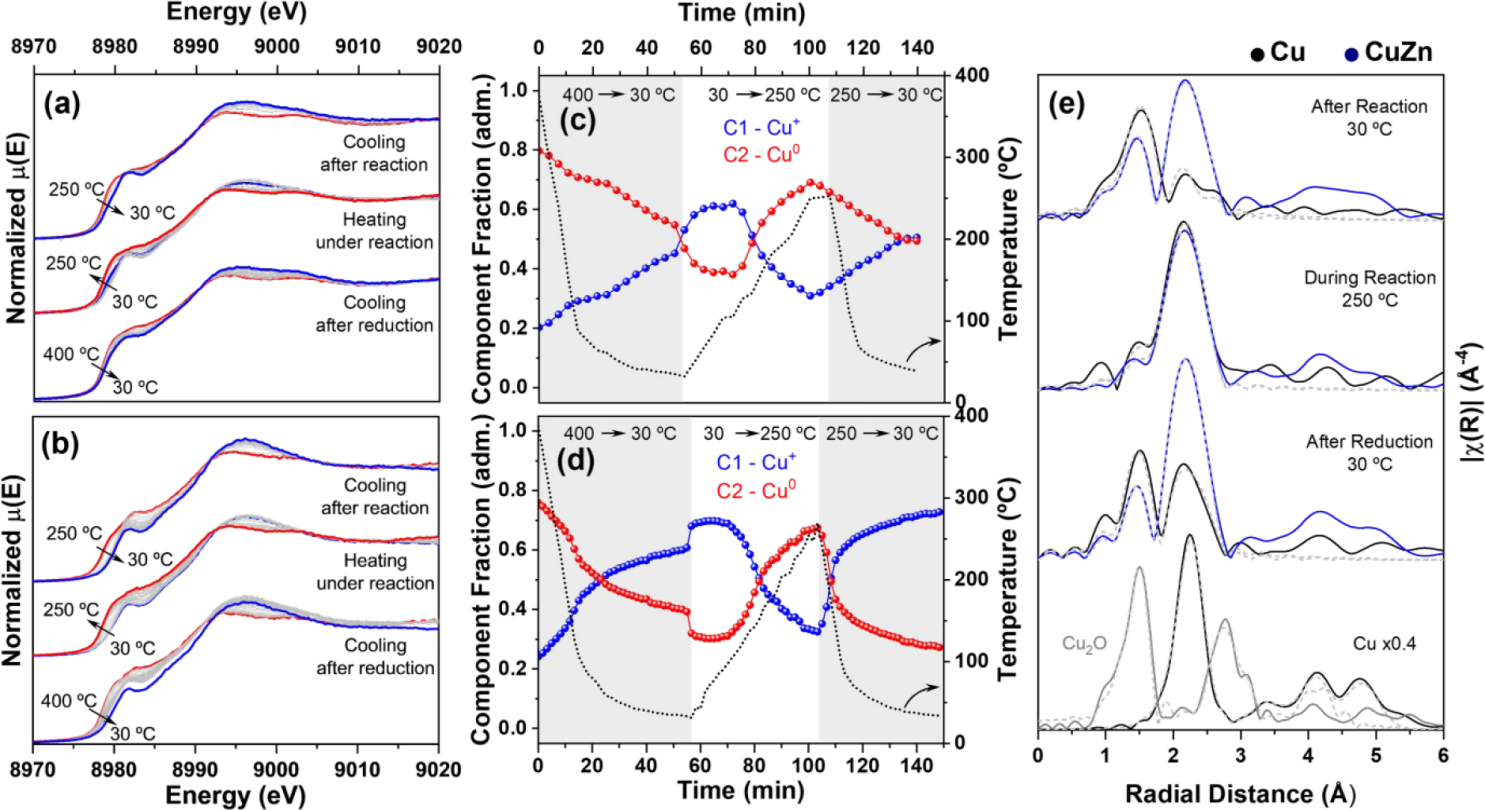
*In situ* XANES in the Cu K-edge during the cooling step after reduction, heating under the reaction atmosphere, and cooling after reaction for (a) CuZn/Al_2_O_3__C500R400 and (b) Cu/Al_2_O_3__C500R400; MCR-ALS components C1 (Cu^0^) and C2 (Cu^+^) as a function of temperature for (a) and (b), respectively; and (e) Corresponding FT of the EXAFS oscillations. Typical k range: 3–11 Å.

The MCR-ALS components C1, corresponding to a typical Cu^+^ phase, and C2, representing Cu^0^ with a feature at 8983 eV, can be seen in Figure S11a,b. For CuZn/Al_2_O_3__C500R400 (Figure 7c), redispersion after reduction followed a single steady regime, independent of temperature, and continued even after the gas atmosphere was switched to the reaction mixture. At 105 °C, oxidic copper began to convert into metallic clusters. After the reaction, the redispersion again followed a single steady regime, with Cu^0^ steadily converting to Cu^+^ during the cooling stage. For Cu/Al_2_O_3__C500R400 (Figure 7d), after reduction, redispersion occurred in two stages: a faster process at temperatures above 66 °C, followed by a slower one as the temperature approached room temperature. After the gas was switched to the reaction mixture, the oxidic and reduced species remained stable until 105 °C, at which point the oxidic contribution began to decrease. At 151 °C, the metallic species were predominant. After the reaction, during the cooling step, redispersion occurred again in a manner similar to that discussed previously. Without the promoter, Cu redispersion into Cu^+^ was rapid at temperatures above ∼70 °C, transitioning to a slower regime below this threshold. In contrast, with the promoter, the redispersion remained consistent across the entire temperature range. This demonstrates that the presence of the promoter tailors the Cu reduction profile, especially affecting the transition between the Cu^0^ and Cu^+^ oxidation states. It is worth highlighting that the redispersed species behaved differently from the initial state. Compared with typical reduction conditions, the Cu^+^ sites formed after reduction were reduced back to Cu^0^ at milder temperatures (<150 °C).

The steady-state EXAFS spectra for Cu/Al_2_O_3__C500R400 and CuZn/Al_2_O_3__C500R400 after reduction, under reaction (250 °C, ambient pressure), and after reaction are presented in Figure 7e (Figure S12 for k-space plotting). The quantitative values extracted from the fitting are summarized in Table 2. After reduction at room temperature, the Cu–O and Cu–Cu coordination numbers (CN) were, respectively, 1.7 and 2.2 for the monometallic catalyst, 0.9 and 5.2, respectively, for the bimetallic catalysts. Consistent with the XANES MCR-ALS results, the results confirmed that in the absence of Zn, Cu was more oxidized, resulting in a cationic Cu rich mixture. For Cu/Al_2_O_3__C500R400, the Cu–O bond distance was 1.89 Å, shorter than the expected value for CuO but longer than the Cu–O bond in Cu_2_O, which could be explained as an average value of both sites Cu^+^ and Cu^2+^. And the Cu–O bond distance was 1.85 Å for CuZn/Al_2_O_3__C500R400, which matches the expected value for Cu_2_O, once again showing that Zn promotes Cu^+^ enrichment.

**Table 2:** EXAFS k^3^-Weighting in the Cu K-Edge Fitting Parameters for Cu/Al_2_O_3__C500R400 (Cu) and CuZn/Al_2_O_3__C500R400 (CuZn) after In Situ Reduction and at Room Temperature (C500R400_RT), under Reaction Conditions at 250 °C (Reaction_250C), and after Reaction at Room Temperature (after reaction_RT)

	C500R400_RT		Reaction_250C		After Reaction_RT	
	Cu	CuZn	Cu	CuZn	Cu	CuZn
**CN Cu–O**	1.7 (3)	0.9 (1)	0.6 (3)	0.5 (6)	2.7 (6)	1.5 (2)
**R Cu–O (Å)**	1.89 (1)	1.85 (1)	1.91 (1)	1.87 (5)	1.91 (1)	1.85 (1)
**σ** ^**2**^ **Cu–O (Å** ^**2**^)	0.004 (2)	0.003 (1)	0.006 (8)	0.01 (1)	0.007 (2)	0.006 (1)
**CN Cu–Cu**	2.2 (7)	5.2 (3)	7 (1)	7.0 (8)	0.6 (5)	2.9 (3)
**R Cu–Cu (Å)**	2.54 (1)	2.52 (2)	2.50 (1)	2.51 (1)	2.56 (2)	2.53 (1)
**σ** ^**2**^ **Cu–Cu (Å** ^**2**^)	0.010 (2)	0.010 (1)	0.014 (1)	0.014 (1)	0.007 (6)	0.008 (1)
**R-factor**	0.011	0.002	0.009	0.002	0.009	0.002
**Reduced χ** ^**2**^	5	8	3	3	4	2
**K range (Å** ^**–1**^)	3–11	3–11	3–11	3–11	3.5–11	3–11
**R range (Å)**	1.1–2.8	1.1–2.8	1.1–2.8	1.2–2.8	1.1–2.9	1.1–2.8

Under reactional mixture (250 °C, ambient pressure), both catalysts showed a similar Cu–O CN of 0.5, indicating Cu^+^ was still present, also the Cu–O bond distances were 1.91 and 1.87 Å for the mono and bimetallic systems, respectively. The metallic particles in both materials were similar to those observed during the reduction step, with Cu–Cu coordination numbers of 7, which were statistically unchanged from those during reduction. After the reaction, the coordination numbers and bond lengths of Cu–O and Cu–Cu remained consistent with the observed patterns, i.e., Cu/Al_2_O_3__C500R400 showed a higher amount of Cu–O with bond lengths of 1.91 Å, while CuZn/Al_2_O_3__C500R400 displayed lower amounts of Cu–O but with a typical 1.85 Å bond length.

The presence of Cu^+^ at 400 °C under a reducing atmosphere or at 250 °C under a hydrogen-rich reaction atmosphere is intimately related to the Al_2_O_3_ capability to stabilize this site.^65^ The stabilization of Cu^+^ sites by alumina was also demonstrated in a detailed experimental and theoretical study carried out by Meng et al.^66^ controlling the Cu:Al ratio of Cu_2_O and amorphous Al_2_O_3_ phases under reducing conditions at 220 °C and 25% H_2_, clearly indicating that alumina can stabilize Cu^+^ even under highly reducing conditions. Regarding the redispersion of the copper moieties, not only the particle size plays an important role, but also the alumina surface functionalization. Fan et al.^60^ reported the redispersion of metallic copper nanoparticles on hydroxylated support surfaces, such as γ-Al_2_O_3_, SiO_2_, and CeO_2_, at room temperature in the presence of humid O_2_, highlighting the key role of hydroxyl groups in this process. Purdy et al.^61^ reported the reduction of Cu^2+^ to Cu^+^ (270 °C, 101 kPa H_2_), followed by full reduction to Cu^0^ after exposure to reactional atmosphere (270–350 °C, 7 kPa EtOH + 94 kPa H_2_). The copper nanoparticles were completely redispersed to Cu^2+^ once regenerated at 550 °C in air, and water played an important role in this redispersion. The work by Liu et al.^67^ has also explored the influence of Cu particle size and steam within the Cu redispersion, pointing out the importance of hydroxyl groups. In the specific case of Cu/ZnAl_2_O_4_, an extensive work by Song et al.^68^ showed that accordingly to the heat treatment, the defects on the zinc aluminate surface can be tuned, and in the scenario where hydroxyl groups are favored, there is also a higher tendency in the stabilization of highly dispersed Cu species.

All the steps taken together are summarized in Figure 8, starting from the Cu^2+^ formed after the precursors’ calcination, the reduction induced the growth of copper clusters and Cu^+^ sites; in the presence of Zn^2+^, the Cu^+^ remained stable throughout a larger temperature range, while we cannot elucidate the exact mechanism behind it. We believe that water and hydroxyl groups on the support surface play an important role. Further experiments with water vapor can be found in the Supporting Information (Figures S13–S15 and Table S5), where *in situ* XAFS experiments suggest that Cu oxidation is more pronounced in the presence of Zn once exposed to water. Once the system was cooled, even though under inert atmosphere, the smaller copper clusters readily dispersed into Cu^+^ and Cu^2+^ sites, while metallic particles larger than 1 nm remained stable. During this step, the presence of Zn favored Cu^+^. These newly redispersed sites (Cu^+^/Cu^2+^) had a different reduction profile to that of the initially calcined sample (Cu^2+^), with reduction taking place at lower temperatures. Heating under the reaction conditions led to the reagglomeration of metallic clusters with the presence of Cu^+^ for both sets of catalysts, and the reduction under CO_2_:H_2_ (1:3, ambient pressure) had slower kinetics. Once the system was cooled, the redispersion was slower, and the larger metallic particle population was increased.

**Figure 8:**
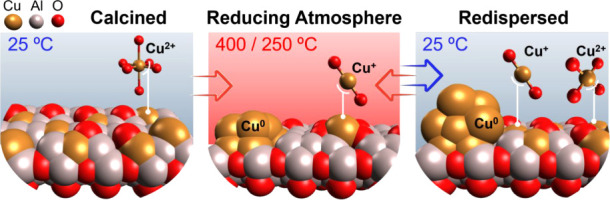
Schematic representation of copper morphological and speciation changes during the reduction of the initial calcined sample, cooling down after reduction, and heating under the reaction conditions of the redispersed phase. The backgrounds in blue and red indicate low (25 °C) and high temperatures (400 or 250 °C), respectively.

### 3.3 The Reaction Mechanism

To further correlate the catalysts structures with their catalytic activity, we studied the reaction mechanism by transient DRIFTS, ME-PSD-DRIFTS, and *operando* DRIFTS. All band assignments are summarized in Table 3, along with a pictorial representation of possible formates binding geometries adapted from ref 80. With the notion that the selectivity drops at higher temperatures, we opted to work at 250 °C to achieve higher sensitivity to intermediates selective to methanol. Figure 9 shows the CO_2_ hydrogenation to methanol in the transient regime and phase domain for the materials reduced at 400 °C, and the corresponding data for soft reduction can be found in Supporting Information Figure S19. We started from Cu/Al_2_O_3_, due to its simpler composition, to be able to isolate the contribution of Cu species and later understand the changes induced by the presence of zinc.

**Figure 9:**
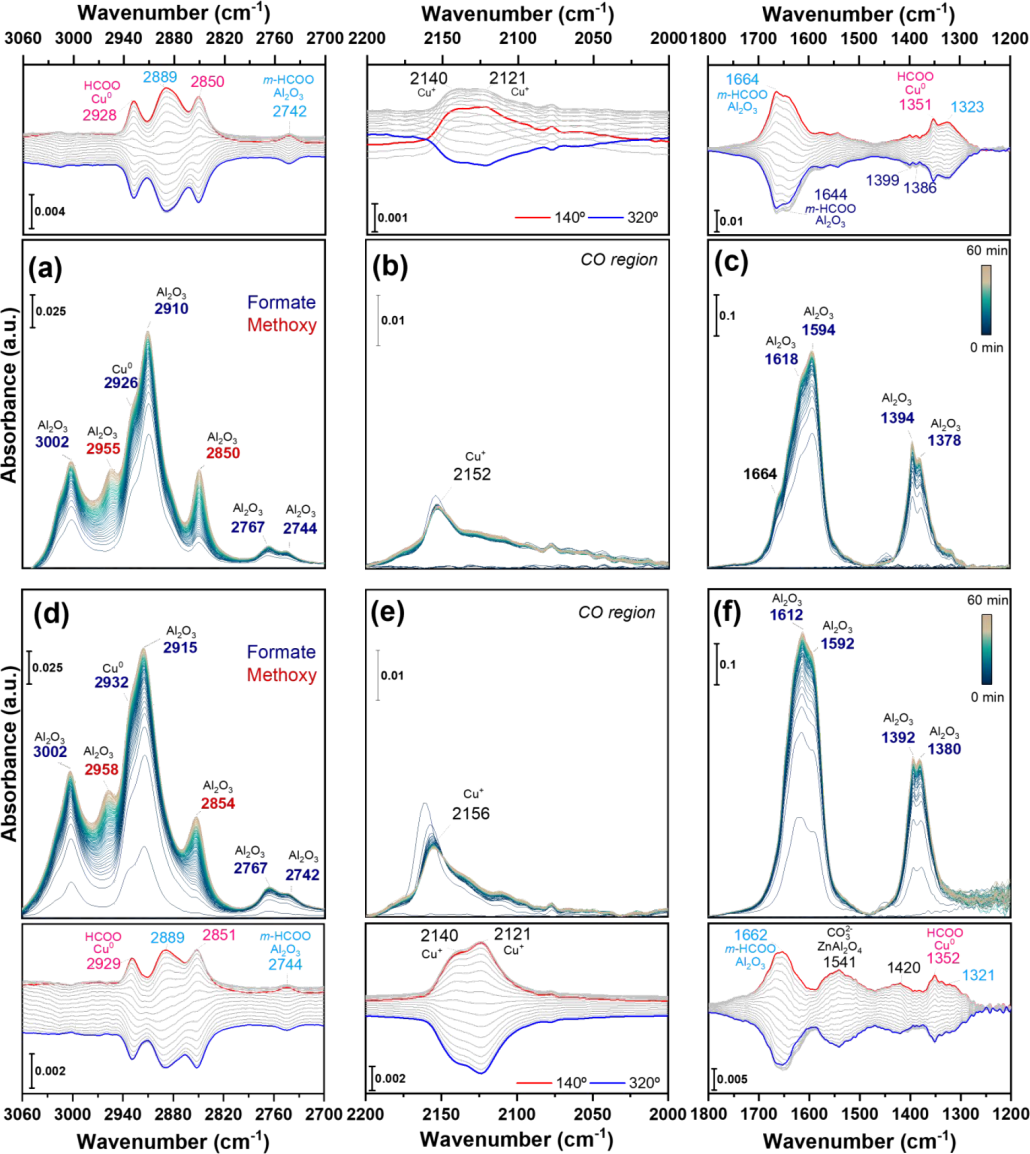
*In situ* reactional DRIFTS spectra as a function of time for (a–c) Cu/Al_2_O_3__C500R400 and (d–f) CuZn/Al_2_O_3__C500R400, highlighting the C–H stretching region (left), carbon monoxide (middle), and C–O stretching (right). Experimental conditions: 1:3 CO_2_:H_2_, 250 °C, 1 bar, 1 h. Formates on alumina are highlighted in blue tones, formate on copper in pink, methoxy in dark red, and CO or carbonate on ZnAl_2_O_4_ in black. ME-PSD-DRIFTS conditions: 250 °C, 1 bar, constant feed of Ar 5 mL·min^–1^ + H_2_ 15 mL·min^–1^, modulation between 5 mL·min^–1^ of CO_2_ or Ar every 60 s.

**Table 3:**
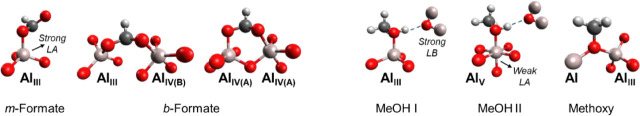
Band Assignments for Cu/Al_2_O_3__C500R400 and the Bare Support γ-Al_2_O_3_ under *Operando* DRIFTS Conditions (250 °C, 20 bar), and for Controlled Chemisorption of Formic Acid (HCO_2_H) and Methanol (CH_3_OH) on γ-Al_2_O_3_

	Wavenumber (cm^–1^)				
	Cu/Al_2_O_3_	γ-Al_2_O_3_			
Species	*In situ*	*In situ*	Control	Assignment	Literature Value (cm^–1^)
*b*-HCOO	3002	-	3005	δ(C–H) + ν_as_(OCO)	2995;^101^ 2996;^28^ 2999 ^105^
Al_2_O_3_	2910	2906	2902	ν(C–H)	2902;^28^ 2904 ^105^
Δν_as‑s_(OCO) = 216	2767	-	2760	δ(C–H) + ν_s_(OCO)	2751;^99^ 2769;^101^ 2769 ^103^
	1594	1592	1590	ν_as_(OCO)	1595;^98^ 1589 ^100^
	1394	-	1392	δ(C–H)	1386;^100^ 1388;^101^ 1392;^28^ 1393 ^106^
	1378	-	1376	ν_s_(OCO)	1384;^97^ 1381;^98^ 1384;^99^ 1382 ^105^
*m*-HCOO Al_2_O_3_	2887	-	2884	ν(C–H)	2878;^97^ 2881;^98^ 2888;^28^ 2876 ^105^
Δν_as‑s_(OCO) = 341	2742	-	2742	δ(C–H) + ν_s_(OCO)	2737;^99^ 2742;^101^ 2742;^101^ 2745 ^106^
	1664	-	1663	ν_as_(OCO)	1664,^107^ 1660 ^93^
	1323	-	1320	ν_s_(OCO)	1326,^101^ 1319 ^93^
HCOO	2928	-	-		2922–2928;^85^ 2930;^86^ 2932–2928;^87^
Cu^0^	2850	-	-	ν(C–H)	2846–2850;^85^ 2849;^86^ 2854–2850;^87^
	1351	-	-	ν_s_(OCO)	1352–1362;^85^ 1355;^86^ 1354–1360;^87^
MeOH I Al_2_O_3_	2850	-	-	ν_s_(CH_3_)	2850;^70^ 2856;^102^ 2858^103^
MeOH II	-	-	2944	ν_s_(CH_3_)	2944 ^24^
Al_2_O_3_	-	-	2824		2825 ^24^
Methoxy	2955	-	2955	ν_s_(CH_3_)	2959;^102^ 2958;^103^ 2960 ^28^
Al_2_O_3_	2844	-	2846		2844 ^24^
	-	1652	-	ν_as_(OCO)	1642;^104^ 1648;^106^ 1658 ^105^
Bicarbonate	-	1438	-	ν_s_(OCO)	1434;^104^ 1438;^106^ 1439 ^105^
	-	1230	-	δ(COH)	1231;^106^ 1227 ^105^
Carbonate	1541	-	-		1530–1540;^88^ 1510;^79^ 1530;^89^ 1534 ^90^
	1420	-	-		1420;^88^ 1415;^79^ 1428 ^90^

The regions of C–H and C–O stretching modes of the DRIFTS spectra of Cu/Al_2_O_3__C500R400 are shown in Figure 9a,c, respectively. From the transient data, intense formate HCOO bands (3002, 2911, 2767, 1594, 1394, and 1379 cm^–1^) were observed that we assigned to bidentate formate species based on the difference between ν_as_(OCO) and ν_s_(OCO) modes (Δ ν_as‑s_(OCO) = 216 cm^–1^).^77^ The shoulder at 1618 cm^–1^ has been reported to be a second type of formate with a slightly different binding geometry, and it is also more resistant to desorption.^78,79^ Analogous signals (3005, 2902, 2760, 1590, 1392, and 1376 cm^–1^) were obtained upon controlled formic acid chemisorption on γ-Al_2_O_3_ (Figure S20) and reaction on the bare support (Figure S21), suggesting that the *b-*HCOO species observed under reaction conditions is bound to the alumina surface.

Two bands at 2955 and 2846 cm^–1^ are correlated to methoxy groups coordinated to the alumina surface based on the controlled methanol chemisorption on γ-Al_2_O_3_ (Figure S20). Methanol can exhibit three adsorption geometries on Al_2_O_3_,^24^ on tricoordinated aluminum sites (Al_lII_) formed under reducing conditions and acting as strong Lewis acids (LA), where the molecule is stabilized by neighboring strong Lewis bases (LB) through hydrogen bonding via the acidic proton of methanol. Methanol chemisorbed at these sites exhibits bands at 2850 cm^–1^ (ν_s_(CH_3_)), 1440 cm^–1^ (δ_s_(CH_3_)) and 1330 cm^–1^ (δ­(OH)). Only the band at 2850 cm^–1^ matches that observed on Cu/Al_2_O_3__C500R400, because the other two are likely overwhelmed by the intense ν­(C–H) formate bands. We refer to this species as MeOH I, whose pictorial representation is provided in Table 3. Additionally, it has been proposed that MeOH I can be converted into a methoxy species stabilized as a bridge between an Al_III_ site and a neighboring Al site (coordination environment not specified), with the hydrogen transferred to a nearby basic site. This methoxy species exhibits bands at 2955 and 2844 cm^–1^,^24^ the former one being visible in our transient experiments, showing the accumulation of both MeOH I and methoxy on the alumina surface during the reaction.

The data for CuZn/Al_2_O_3__C500R400 (Figure 9d,f) corroborates the previous observations. In the transient regime, once the material was exposed to the reaction atmosphere, the same intermediates started to accumulate on the alumina surface. In the presence of Zn, the *b-*HCOO bands (3002, 2915, 2767, 1592, 1392, and 1380 cm^–1^) were blueshifted and Δν_as‑s_(OCO) was 212 cm^–1^. Interestingly, there was an inversion in the relative intensity between the bands at 1612 and 1592 cm^–1^, and the former became more intense. In the case of methanol-related species, the methoxy band shifted to 2958 cm^–1^, and MeOH I to 2854 cm^–1^.

Another important product is CO, which can be formed by the RWGS reaction and can either desorb, contributing to a selectivity loss, or be hydrogenated to methanol. There is evidence for the presence of adsorbed CO during reaction on Cu/Al_2_O_3__C500R400 (Figure 9b) and CuZn/Al_2_O_3__C500R400 (Figure 9e). The broad bands around 2152/2156 cm^–1^ hint at the formation of CO at the oxidized copper (Cu^+^) sites, with no contribution from metallic copper.^30,80^

To determine whether the observed species were truly reaction intermediates or merely spectators, we resorted to ME-PSD-DRIFTS. This is a powerful technique that enhances the signal-to-noise ratio by isolating dynamic signals related to surface processes from static signals. In addition, ME-PSD-DRIFTS is particularly useful for distinguishing between the active and inactive species involved in catalytic reactions, as spectators do not respond to feed modulation and are consequently filtered out by the PSD algorithm. This approach allows for a more accurate interpretation of the reaction mechanisms and identification of true intermediates.^81,82^

Once the material reached a steady state in reaction feed, we performed modulation by switching off and then on the CO_2_ feed in half-periods of 60 s each, resulting in a modulation frequency of 8 mHz, which is of the same order of magnitude as the catalysts’ STY. The phase domain spectra for Cu/Al_2_O_3__C500R400 are shown in the top part of Figure 9a,c for the ν­(C–H) and ν­(C–O) regions, respectively. The intense bands previously observed in the transient experiments and the band at 2955 cm^–1^ completely disappeared in the phase domain, indicating that *b-*HCOO and methoxy species were spectator species that accumulated and did not play a significant role in the reaction. Our data corroborates previous observations that methanol observed in time dependent DRIFTS is not an intermediate.^83,84^

In the phase domain, the new bands at 2889, 2742, 1664, 1644, 1399, 1386, and 1323 cm^–1^ were attributed to two types of formate species. The first one found is characterized by intense bands at 2889, 2742, 1664, and 1323 cm^–1^ with a Δν_as‑s_(OCO) = 341, matching the expected value typical of monodentate formate (*m-*HCOO).^71^ The second species exhibited less intense bands at 1644, 1399, and 1386 cm^–1^, and the high ν_as_(OCO) mode at 1644 cm^–1^ could also be associated with *m*-HCOO. We believe that these new *m*-HCOO species are in close proximity to the copper particle, either directly on the support/particle interface or in the surrounding perimeter. Most importantly, in the phase domain, a third type of formate became evident (2928, 2850, and 1351 cm^–1^) that we associate to metallic copper bound formate (HCOO-Cu^0^) in agreement with previous surface science experiments^85,86^ showing copper bound formate at 2932, 2854, and 1354 cm^–1^ on Cu(111), or at 2928, 2850, and 1360 cm^–1^ on Cu(110).^87^ It is worth highlighting that the band at 2850 cm^–1^ could cause confusion with that of MeOH I, but the relative intensity among 2928, 2850, and 1351 cm^–1^ also phase arguments that will soon be discussed, lead us to believe that in the phase domain 2850 cm^–1^ is related to HCOO-Cu^0^ and not MeOH on alumina.

The behavior of the promoted catalyst, CuZn/Al_2_O_3__C500R400 (Figure 9d,f) was similar to that of the monometallic catalyst. The bands of *b-*HCOO, MeO^–^, and MeOH observed initially disappeared in the phase domain of the ME-PSD-DRIFTS experiments, corroborating the nature of spectators of the related species accumulating on the catalyst surface. Only two formates were found, HCOO-Cu^0^ (2929, 2851, and 1352 cm^–1^) and *m*-HCOO (2889, 2744, 1662, and 1321 cm^–1^), which we believe to be bound on the metal/support interface. Two broad bands were additionally visible at 1541 and 1420 cm^–1^, which are commonly attributed to carbonates (CO_3_^2–^) on ZnAl_2_O_4_.^79,88–90^ The presence of carbonates can be an important step during CO_2_ activation, helping to increase its interaction with the support, and can be associated with the enrichment of basic sites on the surface in the ZnAl_2_O_4_ phase. In fact, the observation of carbonate species in the CuZn sample is consistent with previous reports on ZnAl_2_O_4_, which has been shown to activate CO_2_ and form surface carbonates in the absence of Cu.^78^

### 3.4 The Reaction Mechanism Studied under *Operando* Conditions

The mechanistic studies have so far been conducted at ambient pressure, however the CO_2_ hydrogenation to methanol is typically a high-pressure process. To gain insight into the conditions closer to the real reaction environment, *operando* DRIFTS was performed at 20 bar (Figure 10).

**Figure 10:**
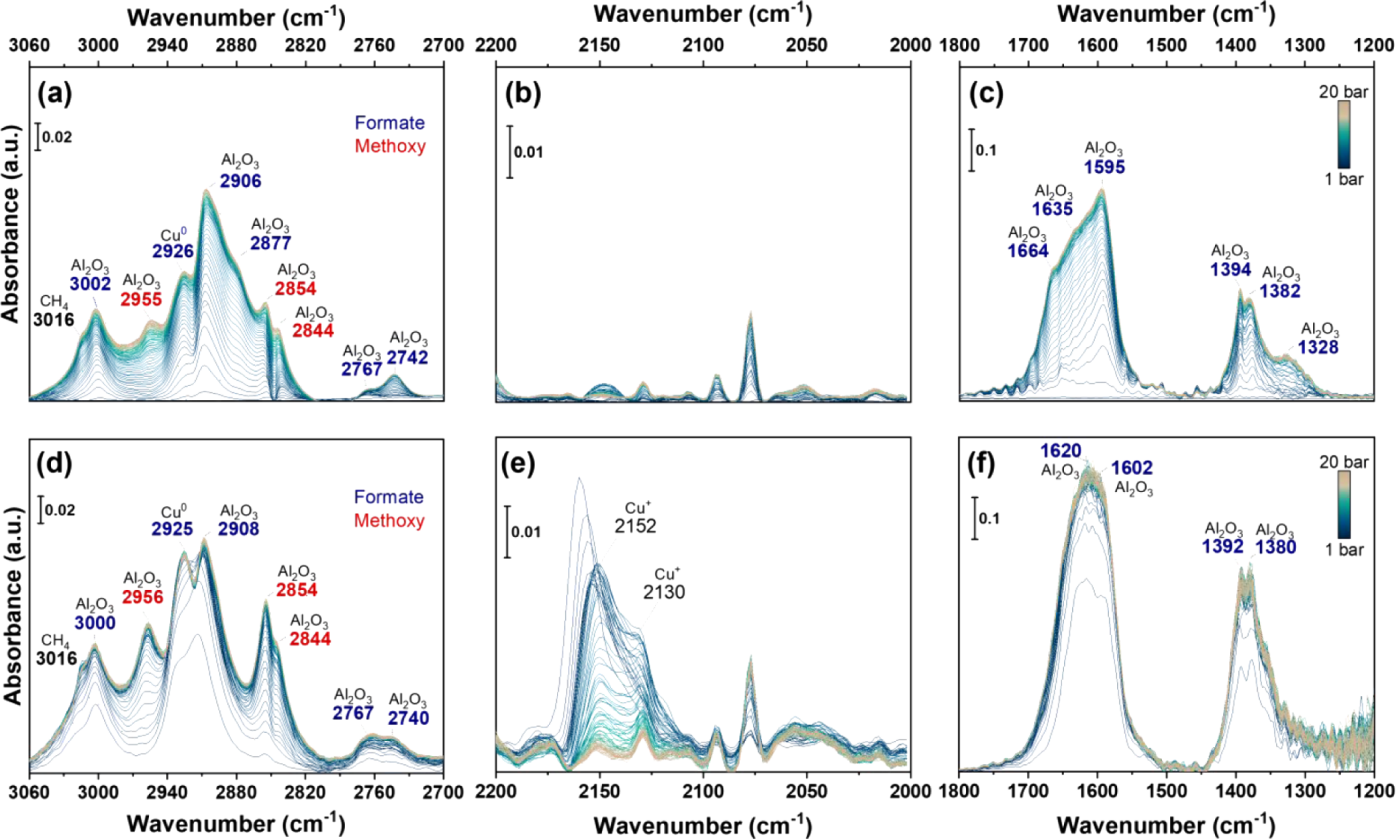
*Operando* reactional DRIFTS as a function of pressure for (a–c) Cu/Al_2_O_3__C500R400 and (d–f) CuZn/Al_2_O_3__C500R400, highlighting the C–H stretching region (left), carbon monoxide (middle), and C–O stretching (right). Experimental conditions: 1:3 CO_2_:H_2_, 30 mL.min^–1^, 250 °C, from 1 to 20 bar.

Upon comparison with the ambient pressure data, it becomes clear that the high pressure induces a broadening of the bands on the monometallic Cu/Al_2_O_3__C500R400 (Figure 10a–c), mainly in the region of the reactive species described above. Under these conditions, the bands at 2926 cm^–1^ for HCOO-Cu^0^ and 2890, 1664, and 1328 cm^–1^ for *m-*HCOO are now distinguishable. In addition, a second band appears at 2844 cm^–1^ (ν­(CH_3_)) for methanol, which is related to methanol chemisorbed to Al_V_ sites and stabilized by neighboring strong Lewis base sites.^24^ Herein, we refer to this species as MeOH II. The presence of MeOH II only under high-pressure conditions can be understood by the weak adsorption on Al_V_ (weak Lewis acid), which is disfavored at ambient pressure and temperatures as high as 250 °C. The same behavior was observed in the soft-reduced samples (Cu and CuZn/Al_2_O_3__C500R200, Figure S22). While Cu/Al_2_O_3__C500R400 had no bands in the CO region (Figure 10b), CuZn/Al_2_O_3__C500R400 (Figure 10e) underwent major changes in this region. As the pressure increased, the band centered at 2156 cm^–1^ shifted to 2152 cm^–1^, and a second band at 2130 cm^–1^ was observed. However, both sites were consumed, falling below the noise level under high-pressure conditions (raw data available in Figure S23). Despite the band covering, we still believe that CO is still playing a role on Cu^+^ sites.

### 3.5 Insights into the Kinetic Contributions of Different Species

The ME-PSD data can also provide essential insights into reaction kinetics from analysis of the phase angles, which represent the lag between the applied stimulus and the observed response. Species exhibiting close phase angular values behave kinetically in a similar manner, whereas significant differences in phase angular values indicate that the species are temporally well separated within the chemical process.^108^ Therefore, PSD can allow for kinetic differentiation of rapid and slower processes, further explanations available in the Supporting Information (Figure S24). For Cu/Al_2_O_3__C500 reduced at both 400 and 200 °C (Figure S25a,b), all three formate species were kinetically different and appeared in the sequence HCOO-Cu^0^ > *m*-HCOO (1664 cm^–1^) > *m*-HCOO (1643 cm^–1^), showing that HCOO-Cu^0^ plays a more relevant role in this catalyst than the support-bound formates. Regarding the band at 2850 cm^–1^ attribution ambiguities, this band is in phase with HCOO-Cu^0^ bands meaning they are related, which excludes any doubt of its assignment to formate rather than methanol. In Cu/Al_2_O_3__C500R200, it was possible to distinguish the methanol band at 1212 cm^–1^ that was out-of-phase with respect to the formates, implying that methanol originates from formates. Meanwhile, CO was the fastest reacting species. On the other hand, for CuZn/Al_2_O_3__C500 reduced at both 400 and 200 °C (Figure S25c,d), kinetic differentiation of the species was more difficult, especially for the catalyst reduced at 200 °C that is enriched in Cu^+^. The intermediates, including CO, possess very close phase angular behavior showing that the Zn promoter accelerates the conversion of supported *m*-HCOO, making it comparable to the more reactive HCOO-Cu^0^. This can help explain the higher activity of CuZn/Al_2_O_3__C500R200 and CuZn/Al_2_O_3__C500R400.

Taken together, these observations help explain the catalytic differences between the promoted and unpromoted catalysts and the effect of pressure. For the promoted catalyst, the presence of ZnAl_2_O_4_ helped to further activate CO_2_ through CO_3_^2–^ intermediates, it was also more selective toward the more reactive *m*-HCOO (1664 cm^–1^), accelerating this intermediate kinetics, making it kinetically comparable to the HCOO-Cu^0^ that was shown to be the fastest formate in the unpromoted catalyst. Zinc aluminate also worked as an indirect promoter by stabilizing Cu^+^ at higher temperatures under reducing conditions, and this site contributed to a second reaction mechanism, i.e., RWGS followed by CO hydrogenation. Our findings corroborate and demonstrate not only the ability to form formate but also the importance of forming the correct type of formate, which can effectively decompose.

### 3.6 Catalytic Performance and Extent of Cu**^+^** Contribution

The catalysts reduced under mild (30% H_2_ at 200 °C) and harsh reducing environments (30% H_2_ at 400 °C) were tested for CO_2_ hydrogenation to methanol in flow conditions to sustain all the structural and mechanistic studies. Their catalytic performance under 1:3 CO_2_:H_2_ at 30 bar from 225 to 300 °C is shown in Figure 11a. At 225 °C, all materials exhibited comparable STY, although a low CO_2_ conversion (∼2%) under these conditions. When the temperature was raised to 250 °C, the materials performed distinctly from each other; in this scenario, those reduced at lower temperature were the best performing materials, where CuZn/Al_2_O_3__C500R200 showed a slightly higher STY value (0.053 g_MeOH_·g_cat_^–1^·h^–1^) than Cu/Al_2_O_3__C500R200 (0.050 g_MeOH_·g_cat_^–1^·h^–1^). At 250 °C, the effect of reduction at a higher temperature on Cu/Al_2_O_3__C500R400 and CuZn/Al_2_O_3__C500R400 negatively affected the catalytic performance, lowering the activity to 0.048 and 0.044 g_MeOH_·g_cat_^–1^·h^–1^, respectively. With an increase in the reaction temperature, the STY improved owing to the higher conversion, with the samples reduced at 200 °C being more responsive to temperature changes, followed by those reduced at 400 °C. For the mildly reduced catalysts, no statistically significant difference in activity was observed at temperatures above 275 °C.

**Figure 11:**
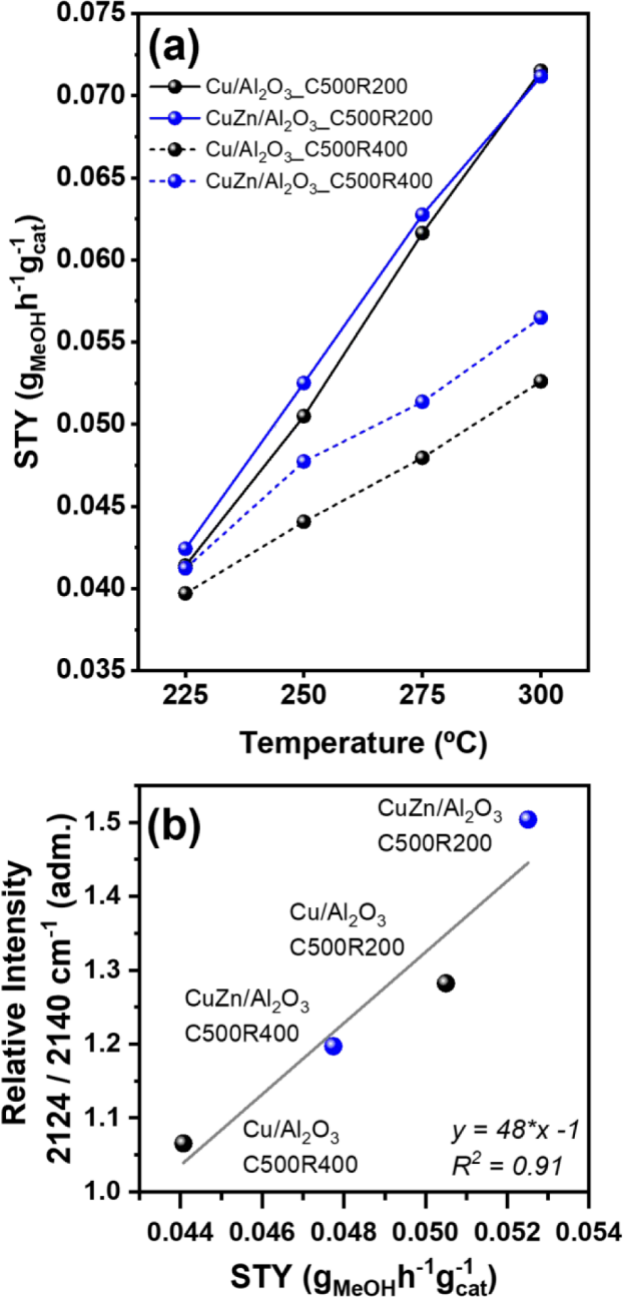
(a) STY as a function of temperature for Cu/Al_2_O_3__C500 and CuZn/Al_2_O_3__C500 reduced at 200 or 400 °C; (b) linear relationship between the Cu^+^ 2124 cm^–1^ band and reaction STY. Experimental conditions: 500 mg catalyst, 30 bar, 140 mL.min^–1^, CO_2_:H_2_ 1:3.

The mild reduction clearly outperformed the harsh reduction. To understand the extent of Cu^+^ sites in this improvement, we focused on the CO contribution within the mechanistic studies. Looking at the time domain DRIFTS (1 or 20 bar) and the phase domain, there are CO contributions at Cu^+^ sites, especially for Zn-promoted catalysts. Both CuZn/Al_2_O_3__C200 (Figure S19e) and CuZn/Al_2_O_3__C400 (Figure 9e) have intense bands at 2153 and 2134 cm^–1^ distinguishable from the background. In the case of Cu/Al_2_O_3__C200 (Figure S19b) and Cu/Al_2_O_3__C400 (Figure 9b), the former had a low-intensity signal and an intense band at 2152 cm^–1^ for the latter. To gain better sensitivity to Cu^+^ bound to CO species and to look at intermediates instead of spectators, we focused on the ME-PSD-DRIFTS data. Interestingly, for the promoted and unpromoted systems (Figure 9b,e) the contributions around 2152 cm^–1^ disappeared during the ME-PSD experiments, demonstrating that they were spectators, and two bands at 2140 and 2121 cm^–1^ became clear, matching exactly the Cu^+^ sites observed during the *in situ* characterization of the catalyst. To understand if this CO was contributing only to the RWGS reaction, or if it was further hydrogenated toward methanol, we plotted the CO_2_ hydrogenation STY against the relative intensity of the 2120/2140 cm^–1^ bands, the use of relative intensity between these bands was to account for inhomogeneities coming from diffuse reflectance. Also considering the data for catalysts reduced under soft conditions (Cu and CuZn/Al_2_O_3__C500R200, Figure S19), we established a linear relationship between the two variables (Figure 11b). The results showed that the higher the amount of Cu^+^ (2124 cm^–1^) in the material, the greater the catalytic activity. The fact that the Cu^+^ sites specifically contribute to the CO mechanism and not the metallic ones can be explained by the nature of the interaction between Cu^+^ and CO molecules. While CO weakly binds to Cu^0^ sites, it moderately binds to Cu^+^, which can lead to an adsorption strong enough to guarantee further conversion and weak enough to avoid poisoning. In fact, our CO-TPD experiments (Figure S16) showed that most of the Cu^+^ bound CO resisted up to 150 °C before being completely desorbed.

As a matter of fact, early works by Nakamura et al.^91,92^ on the Cu/ZnO system sustained that CO_2_ and CO hydrogenations take place at different copper sites, with the former being promoted by CuZn alloys and the latter occurring at Cu^+^ sites in the Cu–O–Zn interface. In recent years, the role of Cu^+^ has been revisited, with isolated Cu^+^ sites showing a direct correlation with methanol formation. For instance, small colloidal Cu nanoparticles (∼2 nm) deposited on Cu-MgO-Al_2_O_3_ under reaction conditions lead to the generation of Cu^+^ sites owing to the influence of water. A linear relationship between the catalytic activity and the presence of cationic copper was established. However, caution is necessary, as these correlations are based on Cu^+^ quantification via “*ex situ*” techniques. This work also emphasizes the importance of monodentate formate formation in the reaction mechanism.^93^ The same group later proposed a mechanism supported by theoretical studies, which suggested that *m*-HCOO is stabilized by Cu^+^.^94^ Another study on Cu/SiO_2_ demonstrated a stronger stabilization of Cu^+^ sites because of the synthetic method used (flame spray pyrolysis), and *in situ* DRIFTS studies supported the CO mechanism at these sites.^70^ A recent work by Li et al.^109^ unambiguously showed a clear linear relationship between the density of Cu^+^ sites with STY in model ZnO/Cu catalysts. In addition to aluminate and silicate supports, Cu^+^ sites have also been shown to be significant in Cu/ZrO_2_ systems, as recently reported.^95^ In the typical methanol synthesis conditions (CO + CO_2_ + H_2_) detailed microkinetic studies have shown that direct CO hydrogenation can contribute up to one-third of the methanol produced.^96^ Recent DFT calculations also helped to elucidate the preferable CO_2_ activation in metallic Cu^0^, and CO activation in Cu^+^ from Cu_2_O surface.^110^

The CO and formate hydrogenation pathways are not competitive when they take place at different Cu species, i.e., oxidic (Cu^+^) and metallic (Cu^0^) sites, and both can contribute to catalytic activity. Figure 12 summarizes our mechanistic proposal according to the copper species. On metallic copper clusters, CO_2_ is initially hydrogenated to formate, here represented as bidentate formate *b-*HCOO, as proposed in previous DFT studies.^111–113^ Based on previously published DFT studies we believe that both *b-*HCOO on copper particle and *m*-HCOO in the interface with the support can be hydrogenated to HCOOH, H_2_COOH, then this intermediate breaks into −OH and formyl (H_2_CO) that is further reduced toward H_3_CO. Finally, the methoxy species can either abstract a −H group and desorb as methanol or diffuse further into the alumina surface. In the absence of promoter, the copper-bound formate is the most reactive species, with a majoritarian contribution to the reactional mechanism. The diffusion of formate species away from the copper center gives rise to most of the spectator *b*-HCOO observed in regular DRIFTS experiments. On the Cu^+^ sites, we propose an initial RWGS step converting CO_2_ into CO with the aid of neighboring −OH groups, followed by consecutive additions of −H to the carbonyl until complete hydrogenation to methoxy and finally methanol. Zinc in the ZnAl_2_O_4_ phase promotes the reaction mechanism by offering a second route for CO_2_ activation through the formation of reactive carbonates. It is also more selective toward the reactive *m*-HCOO, improving its kinetics, making it comparable to the HCOO-Cu^0^. Zinc aluminate also functioned as a structural promoter stabilizing the Cu^+^ species at higher temperatures, which in turn worked as a second route for CO_2_ conversion by means of RWGS and CO hydrogenation.

**Figure 12:**
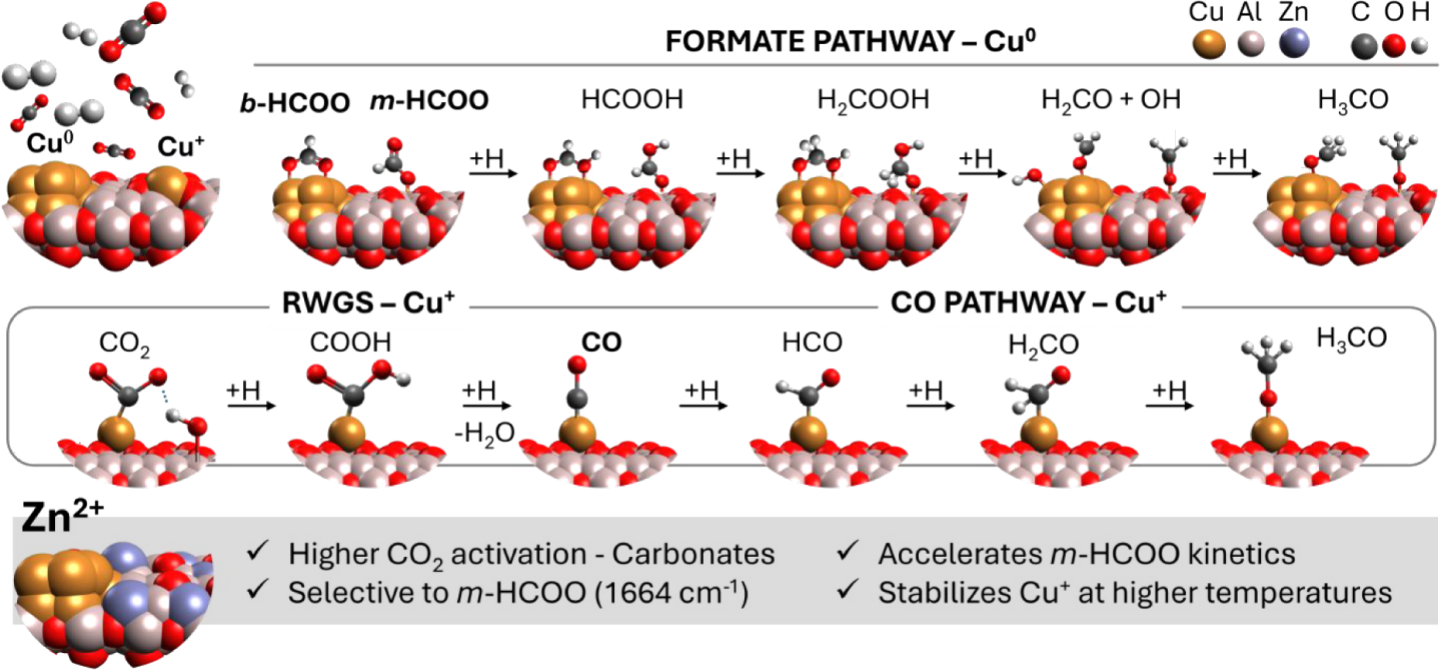
Possible reaction mechanisms according to copper sites, formate mechanism for metallic copper clusters (Cu^0^), and RWGS + CO pathway for oxidic copper (Cu^+^) sites.

## 4 Conclusions

Transient spectroscopic techniques, such as MCR-ALS and ME-PSD-DRIFTS, offer substantial advantages in deciphering complex catalyst behaviors under realistic reaction conditions. To the best of our knowledge, this study represents the first instance where these complementary approaches have been simultaneously employed to unambiguously identify active species and reactive intermediates in CO_2_ hydrogenation. This methodological synergy promises significant advancements for catalytic research and rational catalyst design.

Herein, we studied a highly dispersed CZA catalyst and its unpromoted analog for the direct CO_2_ hydrogenation to methanol. We demonstrated that copper catalysts are highly dynamic, even in controlled model systems, underscoring the importance of *in situ* experiments to better correlate structural characteristics with catalytic performance. Under a reducing atmosphere, even at temperatures as high as 400 °C, Cu^+^ sites were stable on the Al_2_O_3_ surface. Cooling under inert atmosphere produced a size-dependent redispersion of the metallic particles into Cu^+^/Cu^2+^, with larger particles being more resistant to dispersion. After exposure to the reaction atmosphere, the redispersed sites agglomerated back into metallic clusters. Our experiments revealed that the hydrogenation of CO_2_ to methanol is a complex process that responds differently to copper species. The reactivity of Cu/Al_2_O_3_ and CuZn/Al_2_O_3_ was rationalized considering the formate species coordinated to the metallic copper clusters and their vicinity to the support, thus highlighting the key role of copper in CO_2_ activation and H_2_ dissociation. At the cationic Cu^+^ sites, we showed evidence of CO_2_ activation through the RWGS reaction, followed by CO hydrogenation. In the case of bimetallic CuZn/Al_2_O_3_, Zn was incorporated into the alumina lattice to form ZnAl_2_O_4_, which offered a second mechanism for CO_2_ activation through carbonate formation. It also promoted the formation of the more reactive *m*-HCOO and its conversion. As a structural promoter, ZnAl_2_O_4_ favored the formation of Cu^+^ depending on the reduction temperature, which significantly influenced the catalytic behavior.

Our findings emphasize the importance of promoting the formation of the optimal metal species of Cu or the support depending on the input feed and operating conditions and how this correlates with the formation of particular intermediates and their subsequent evolution via hydrogenation steps.

## Supplementary Material



## Data Availability

Raw spectroscopy data (UV–vis, IR, XAFS) recorded on Cu/Al_2_O_3_ and CuZn/Al_2_O_3_ model catalysts during calcination, reduction and reaction (CO_2_ hydrogenation) can be found via the following doi: 10.5522/04/30456467.

## Notes

Detailed synthesis procedures for the Cu and CuZn catalysts and additional spectroscopic data (e.g., DR-UV–vis, XPS, EXAFS, XANES), fitting and analysis (PDF)
