# Supplementary material for: Identification of Transient Intermediates and Active Species in Atomic CZA Catalysts for CO2 Hydrogenation to Methanol
Source: J Am Chem Soc. 2025 Nov 11;147(47):43295–316. doi: 10.1021/jacs.5c08043 (PMC12673602; doi:10.1021/jacs.5c08043)
Supplement: Supplementary file 1 [file ja5c08043_si_001.pdf]

SUPPORTING INFORMATION

Identification of Transient Intermediates and Active Species in Atomic CZA Catalysts for CO_2 Hydrogenation to Methanol

Leonardo da Silva Sousa[1,2,3], Andrea Bertuzzi[4], Tanna Elyn Rodrigues Fiuza[5], Edson Roberto Leite[5,7], Patricia Benito[4], Davide Ferri[6], Daniela Zanchet[1,*] and Andrew M. Beale[2,3,*]

[1]*Instituto de Química, Universidade Estadual de Campinas, Cidade Universitária Zeferino Vaz, Campinas, São Paulo, 13083-970 Brazil*

[2]*Department of Chemistry, University College London, London, WC1H 0AJ, UK*

[3]*Research Complex at Harwell, Rutherford Appleton Laboratory, Harwell Science and Innovation Campus, Didcot, OX11 0FA, UK*

[4]*Department of Industrial Chemistry, Università di Bologna, Viale Risorgimento 4, Bologna, 40136, Italy*

[5]*Brazilian National Nanotechnology Laboratory (LNNano), CNPEM, Campinas, São Paulo 13083-970, Brazil*

[6]*Paul Scherrer Institute, PSI Center for Energy and Environmental Sciences, Villigen CH-5232, Switzerland*

[7]*Chemistry Department, Federal University of São Carlos (UFSCar), São Carlos, São Paulo 13565-905, Brazil*

*Corresponding authors: andrew.beale@ucl.ac.uk, zanchet@unicamp.br

1. Detailed Precursor Synthesis

1.1. Ligand and Complexes Synthesis

Valen: In a 250 ml round-bottom flask, 120 ml of ethanol was added, followed by *o*-vanilin (1.00 g, 6.58 mmol) and 1,2-ethylenediamine (220 μl, 3.29 mmol), the solution was stirred under reflux for 2 h. Then, cooled down to room temperature, and the yellow precipitate was filtered and washed with cold ethanol (3 x 10 ml) and cold diethyl ether (3 x 10 ml). **Yield:** 88%. **MP:** 164.4 ºC. **^{1}HNMR** (250 MHz, $CDCl_3$): d = 13.58 (s, 2H), 8.33 (s, 2H), 6.89 (m, 2.4H), 6.80 (m, 3.4H), 3.96 (s, 4H), 3.89 (s, 6H). **IR (ATR)** υ/cm^{-1}: 2998 (w, νCH), 2930 (w, νCH), 1630 (s, νC=N), 1459 (s, νC=C), 1244 (s, νCO), 1079 (m, δCH, δCH2). **UV-Vis** (Ethanol): 266, 303, and 334 nm.

Cu(valen): In a 250 ml round-bottom flask, 60 ml of ethanol was added and heated up to 75 ºC, then (547 mg, 1.67 mmol) of valen ligand was added. Once the ligand was completely dissolved, (332 mg, 1.67 mol) of $Cu(AcO)_2$ in 30 ml EtOH was dropwise added. The solution was stirred under reflux for 2h, then concentrated until 30 ml of solvent was left, and the dark green precipitated filtered and washed with cold ethanol (3 x 10 ml) and cold diethyl ether (3 x 10 ml). **Yield:** 62%. **MP:** 257 ºC. **^{1}HNMR** (250 MHz, $CDCl_3$): the isolated product was paramagnetic, and the peaks were broad. **UV-Vis** (Ethanol): 284, 382, and 568 nm.

CuZn(valen): In a 100 ml round-bottom flask with 15 ml of MeCN:EtOH 7:3 solution at 75 ºC, (40.4 mg, 0.10 mmol) of Cu(valen) complex was solubilized, then a solution of (30.8 mg, 0.10 mol) of $Zn(NO_3)_2.6H_2O$ solubilized in 5 ml of the same solvent mixture was quickly added to the solubilized Cu(valen). Then, the final solution was kept under agitation and heating until completely dry. The light brown precipitate was washed with cold ethanol (3 x 10 ml) and cold diethyl ether (3 x 10 ml). **Yield:** 95%. **MP:** decomposition. **^{1}HNMR** (250 MHz, $CDCl_3$): the isolated product was paramagnetic, and the peaks were broad. **UV-Vis** (Ethanol): 284, 295, 379, and 568 nm.

2. *In situ* Diffuse Reflectance UV-Vis (DR-UV-Vis)

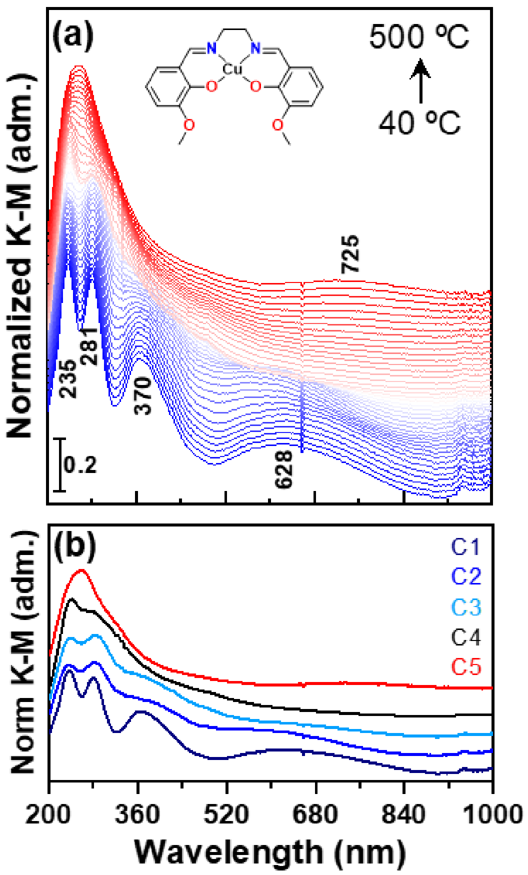

Figure S1. (a) *In situ* DR-UV-Vis calcination; (b) the corresponding MCR-ALS components of Cu(valen)/Al_2O_3. Experimental conditions: 20% O_2 in Ar, 50 ml.min^{-1}, 10 °C.min^{-1}, from 40 to 500 °C.

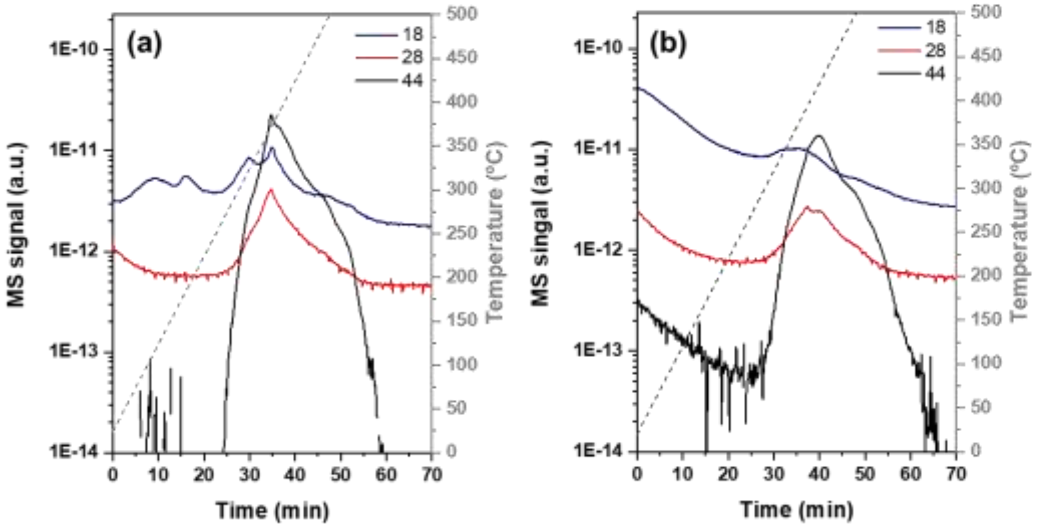

Figure S2. Mass spectrometry results from the *in situ* DR-UV-Vis for (a) Cu(valen)/Al_2O_3; (b) CuZn(valen)/Al_2O_3. Experimental conditions: 20% O_2 in Ar, 50 ml.min^{-1}, 10 °C.min^{-1}, from 40 to 500 °C. Fragment 18 = H_2O, 28 = CO, and 44 = CO_2.

Table S1. EXAFS at Cu K-edge with k^2-weighting fitting parameters for Cu/Al_2O_3_C500 and $CuZn/Al_2O_3$_C500 (Cu_C5 and CuZn_C5, respectively. Cu foil, Cu_2O and CuO standards were also included.

	Cu_foil	Cu_2O	CuO	Cu_C5	CuZn_C5
**CN Cu-O(1)**	-	2	4	2.8 (2)	2.6 (2)
**R Cu-O(1) (Å)**	-	1.85 (1)	1.96 (1)	1.94 (1)	1.94 (1)
**σ^2 Cu-O(1) (Å^2)**	-	0.003 (1)	0.004 (1)	0.003 (1)	0.003 (1)
**CN Cu-Cu**	12	12	4	-	-
**R Cu-Cu (Å)**	2.54	2.99 (1)	2.91 (1)	-	-
**σ^2 Cu-Cu (Å^2)**	0.009	0.014 (1)	0.007 (1)	-	-
**R-fator**	0.02	0.02	0.007	0.008	0.01
**Reduced χ^2**	614	343	438	15	11
**k (Å^{-1})**	3.2 - 16	1.8 - 16	2 - 16	1.2 – 13	1.2 – 13
**R (Å)**	1.3 – 5.2	1.1 – 4.2	1.2 – 3.8	1.15 – 2.3	1.1 – 2.3

3. *Quasi in situ* X Ray Photoelectron Spectroscopy (XPS)

To further understand the species distribution between the bulk and the surface of the support, we analyzed the samples by XPS, and the spectra are shown in Figures S3a and S4, with the attributions summarized in Table S2. All three materials had an experimental Cu:Al ratio of 0.01, which was very close to the theoretical value of 0.008, indicating that Cu was located on the surface. For Zn, the experimental Zn:Al ratio is 0.02 above the expected theoretical 0.01, showing that the surface is indeed enriched with Zn. Table S3 presents the quantitative results.

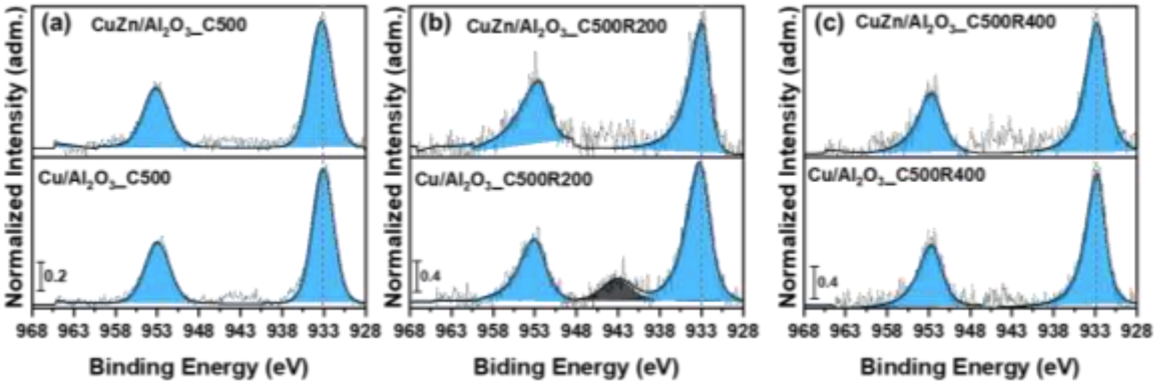

Figure S3. *Ex situ* XPS spectra of Cu 2p region for (a) calcined alumina supported Cu/Al_2O_3_C500 and $CuZn/Al_2O_3$_C500; and *Quasi in situ* XPS spectra of Cu 2p region for same set of catalysts (b) reduced at 200 ºC; (c) reduced at 400 ºC.

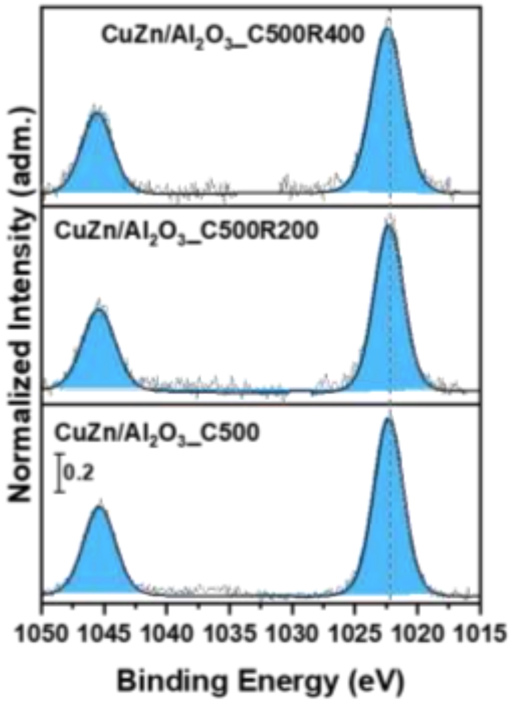

Figure S4. *Ex situ* (calcined sample) and *Quasi in situ* (reduced samples) XPS spectra of Zn 2p region for $CuZn/Al_2O_3$.

Table S2. Band position, FWHM, and atomic ratio results extracted from XPS spectra of the Cu 2p region for alumina-supported calcined samples.

Sample	Position eV	FWHM	Species	Ratios Cu:Al[a]	Zn:Al[b]
Cu/Al_2O_3_C500	933.1	3.02	Cu^{2+}	0.01	-
$CuZn/Al_2O_3$_C500	933.3	3.23	Cu^{2+}	0.01	0.02

Expected values: a = 0.008; b = 0.01

Table S3. Band position, FWHM and atomic ratios results extracted from XPS spectra of Cu 2p region for alumina supported calcined, and calcined and reduced samples.

Sample	*Position* *eV*	*FWHM*	*Specie*	*Ratios*	
				Cu:Al[a]	*Zn:Al*[b]
**Cu/Al2O3_C500**	933.1	3.02	Cu^{2+}	0.01	-
**CuZn/Al2O3_C500**	933.3	3.23	Cu^{2+}	0.01	0.02
**Cu/Al2O3_C500R200**	933.2	2.91	Cu^{2+}	0.01	-
**CuZn/Al2O3_C500R200**	932.9	2.05	Cu^{+}	0.01	0.02
**Cu/Al2O3_C500R400**	932.8	2.41	Cu^{+} (?)	0.01	-
**CuZn/Al2O3_C500R400**	932.8	2.80	Cu^{+} (?)	0.01	0.02

4. XAFS

EXAFS data were modeled using the Artemis module of the Demeter software package, employing theoretical scattering paths generated by FEFF6. The structural models were built from the crystallographic data of reference compounds (e.g., Cu metal, Cu_2O, ZnO, and $ZnAl_2O_4$), which were used to extract relevant Cu–O, Cu–Cu, and, Zn–O scattering paths. The scattering paths were selected based on their relevance as indicated by the FEFF6 simulations. During the fitting, the amplitude reduction factor (S_o^2) was fixed to 0.9, based on fitting of reference metal foils. The energy shift (ΔE_o) was initially allowed to vary during the fitting of the first coordination shell and then fixed at the optimized value once a satisfactory fit was achieved. The coordination numbers (N) were refined with no constraints. Debye–Waller factors (σ^2) were refined independently for each path with no constraints.

All fits were performed in R-space with a k-weighting of 2, within a k-range of 3 to 11.5 $Å^{-1}$ and an R-range appropriate for the scattering shell (typically 1.0 to 3.0 Å). Fitting quality was assessed using the reduced χ^2 and R-factor values, as well as visual agreement between experimental and modeled spectra. For the particle size estimation from the coordination numbers, a Cu–O coordination number of ~0.5 was observed for Cu/Al_2O_3_C500R400 and $CuZn/Al_2O_3$_C500R400. Assuming Cu^+ species are primarily linearly coordinated (CN = 2), this Cu–O CN corresponds well with an oxidized fraction of ~25%. Consequently, the Cu–Cu coordination number obtained directly from EXAFS (CN = 5.9) was corrected to reflect only the metallic fraction by dividing by 0.75, yielding a

corrected CN of ~7.9. This value was then used in a semi-spherical particle model to estimate the nanoparticle size, resulting in a diameter of ~1.8 nm.

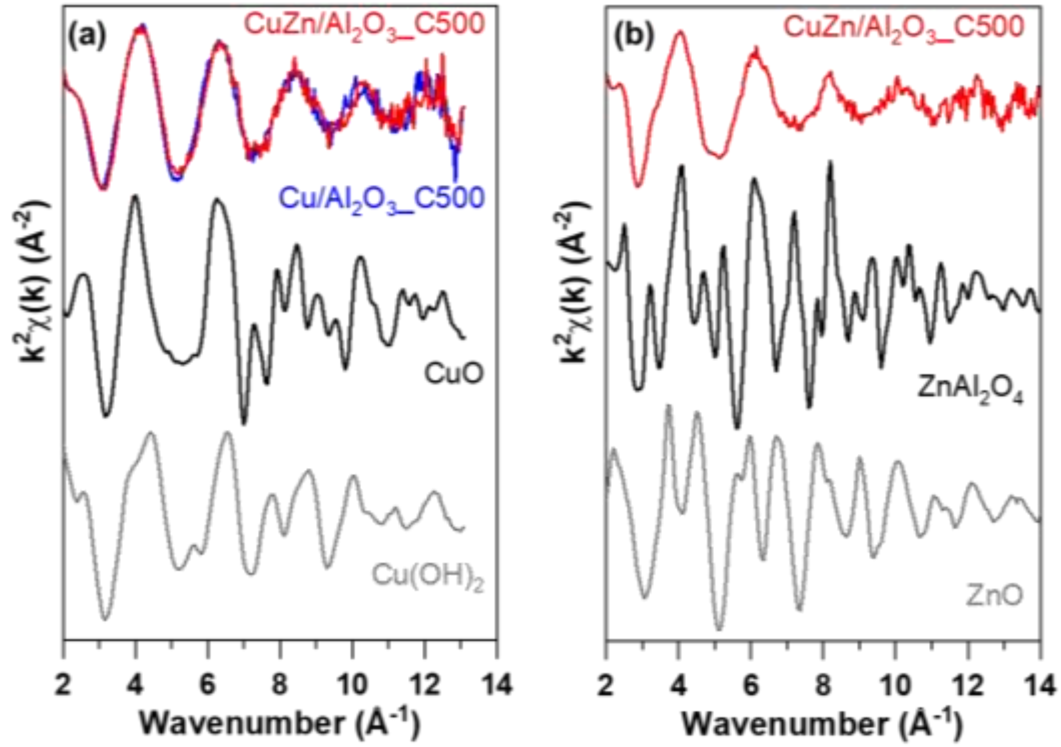

Figure S5. Oscillations in k-space (a) at Cu K-edge for Cu/Al_2O_3_C500 and CuZn/Al_2O_3_C500; (b) at Zn K-edge for CuZn/Al_2O_3_C500. The standards CuO, $Cu(OH)_2$, ZnO, and $ZnAl_2O_4$ were added for reference.

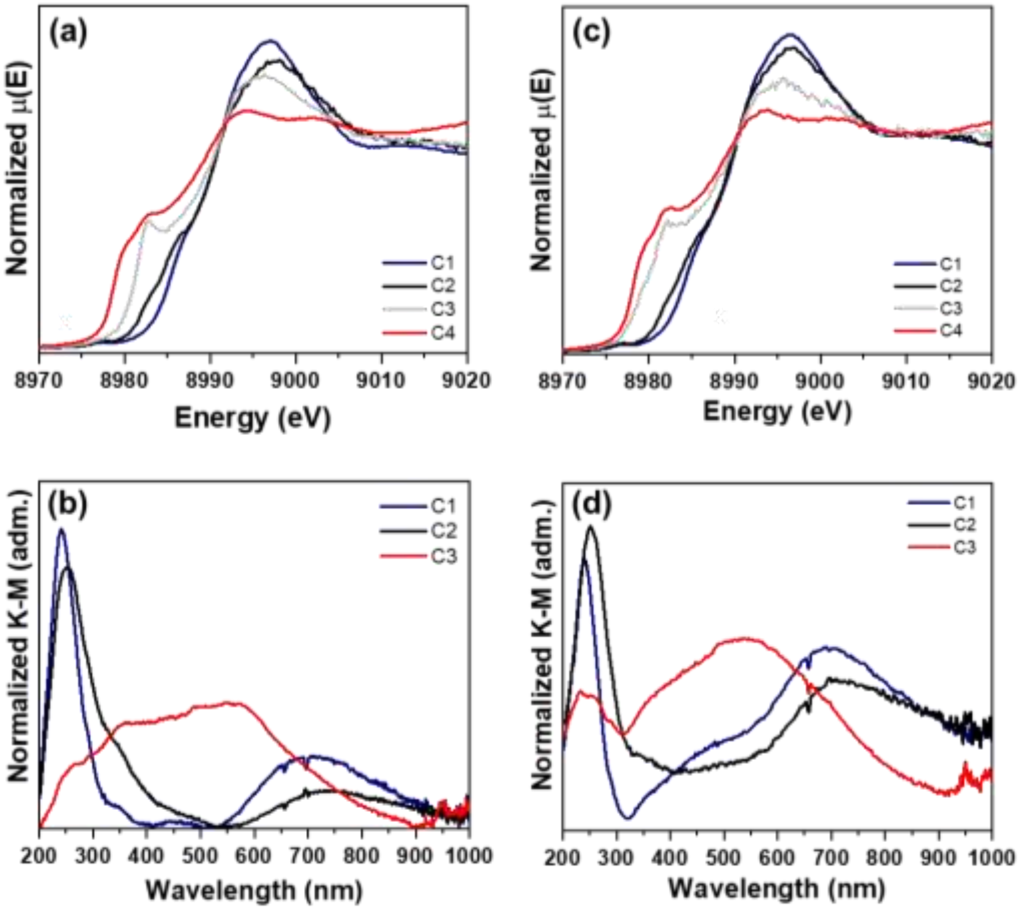

Figure S6. MCR-ALS components extracted from *in situ* (a) XANES and (b) DR-UV-Vis reduction experiments for Cu/Al_2O_3_C500, and the corresponding components for *in situ* (c) XANES and (d) DR-UV-Vis for CuZn/Al_2O_3_C500.

To gain further insights in the Cu^+ morphological nature, we compared the Cu^+ MCR-ALS extracted component to a reference Cu_2O XANES spectrum. As shown in Figure S7, the MCR-derived Cu^+ spectrum displays a broadened white line and lacks the sharp post-edge multiple scattering features (notably at 9010.7 and 9015.0 eV) that are typical of crystalline Cu_2O. This is consistent with a disordered and highly dispersed copper environment, as would be expected for isolated atoms or non-crystalline clusters. These findings support the interpretation that the Cu^+ component arises from single atoms or non-crystalline sites rather than extended oxide domains.

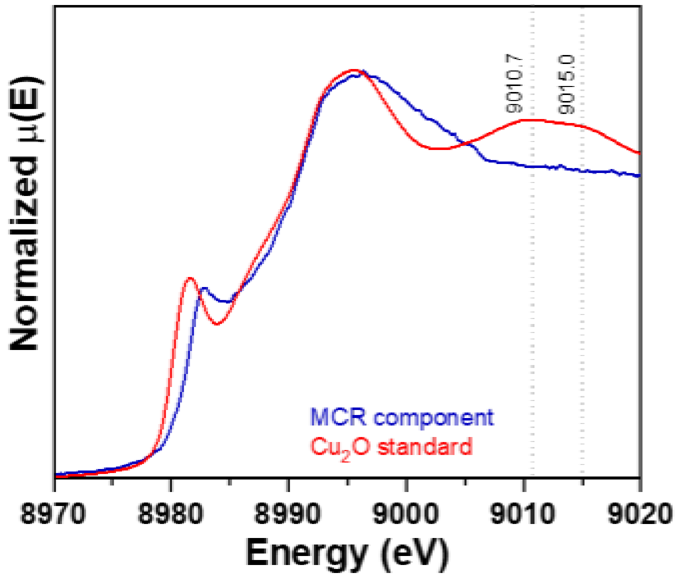

Figure S7. XANES spectrum at the Cu K-edge for Cu_2O reference material, and MCR-ALS component "C3" extracted from *in situ* XANES during reduction experiments.

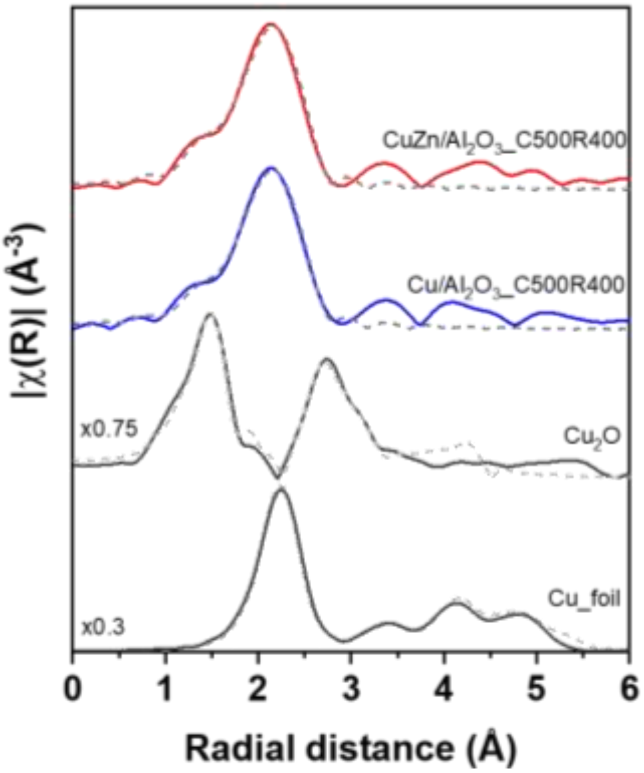

Figure S8. EXAFS spectra on Cu K-edge for Cu/Al_2O_3_C500R400 and $CuZn/Al_2O_3$_C500R400 measured at 400 ºC. References: Cu foil and Cu_2O. Solid lines: measured profiles; dashed lines: fitting results.

Table S4. EXAFS k^2-weighting fitting parameters for Cu/Al_2O_3_C500R400 and $CuZn/Al_2O_3$_C500R400 (Cu_C5R4 and CuZn_C5R4, respectively) at Cu K-edge; $CuZn/Al_2O_3$_C500 (CuZn_C5) and $CuZn/Al_2O_3$_C500R400(CuZn_C5R4) at Zn K-edge. $ZnAl_2O_4$ standard was also included.

	**Cu K-edge**		**Zn K-edge**		
	**Cu_C5R4**	**CuZn_C5R4**	**$ZnAl_2O_4$**	**CuZn_C5**	**CuZn_C5R4**
**CN M-O**	0.4 (2)	0.4 (2)	4	2.9 (3)	2.6 (4)
**R M-O (Å)**	1.87 (3)	1.88 (2)	1.93 (3)	1.96 (1)	1.95 (2)
**σ^2 M-O (Å^2)**	0.01 (1)	0.002 (7)	0.002 (1)	0.005 (2)	0.007 (3)
**CN Cu-Cu**	5.6 (8)	5.9 (9)	-	-	-
**R Cu-Cu (Å)**	2.50 (1)	2.50 (1)	-	-	-
**σ^2 Cu-Cu (Å^2)**	0.016 (2)	0.015 (2)	-	-	-
**R-fator**	0.007	0.004	0.004	0.004	0.009
**Reduced χ^2**	8	7	63	31	27
**k (Å^{-1})**	3 – 10	3 – 10	3 – 12	3 – 11	3 – 11
**R (Å)**	1.1 – 2.9	1.1 – 2.9	1.1 – 2.5	1.1 – 2.5	1.1 – 2.5

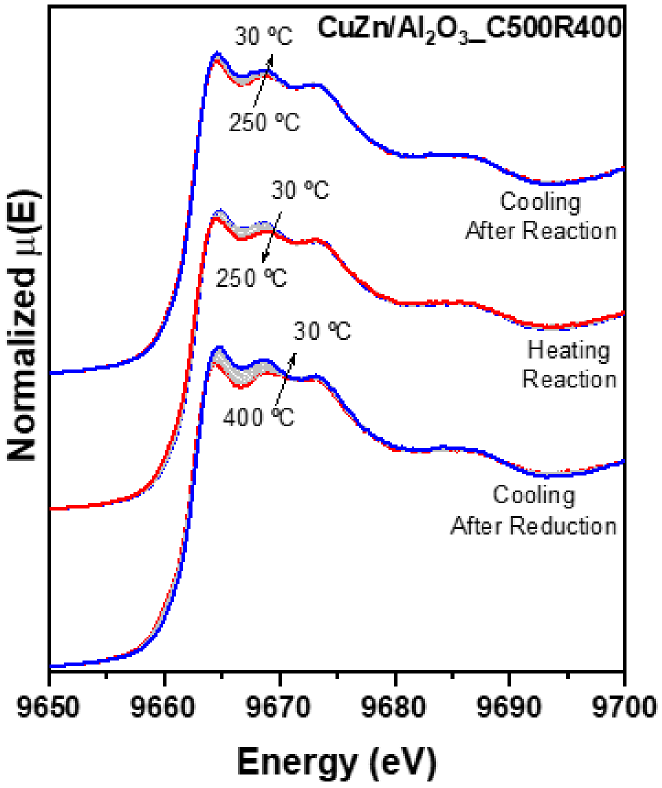

Figure S9. *In situ* XANES in the Zn K-edge during cooling step after reduction, heating under reactional atmosphere, and cooling after reaction for $CuZn/Al_2O_3$_C500R400.

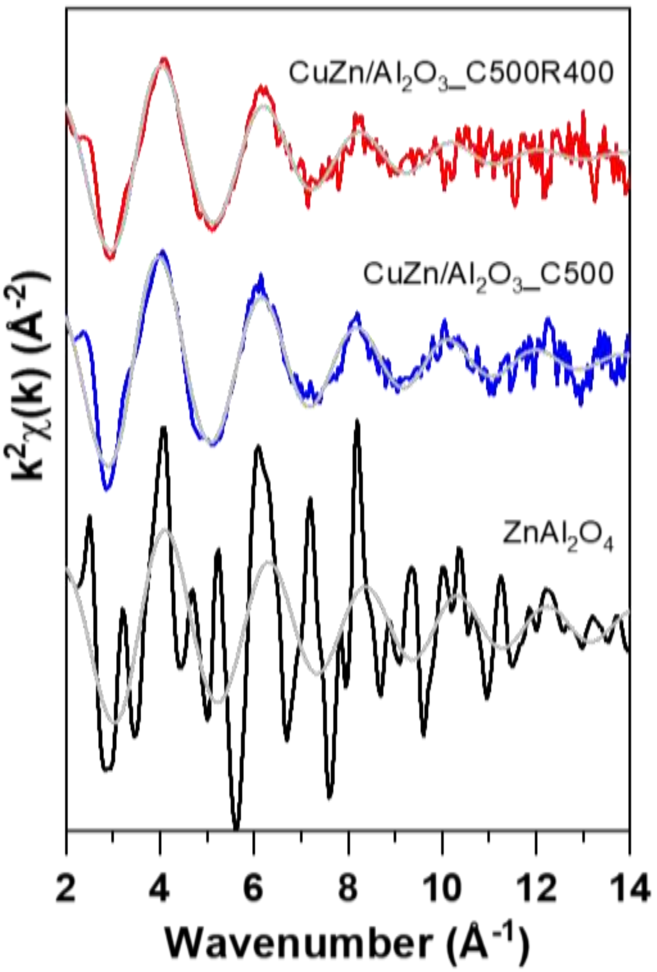

Figure S10. EXAFS oscillations in k-space at Zn K-edge for (a) CuZn/Al_2O_3_C500 and (b) CuZn/Al_2O_3_C500R400. The standard $ZnAl_2O_4$ was added for reference.

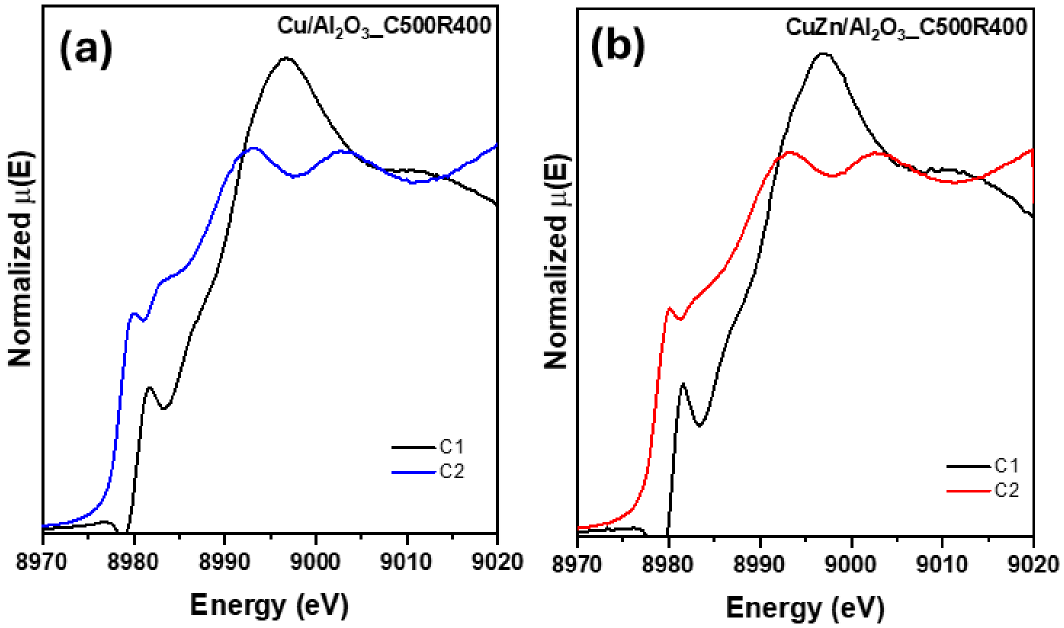

Figure S11. MCR-ALS components C1 (Cu^{+}) and C2 (Cu^{0}) extracted from *in situ* XANES during cool down step after reduction, reactional conditions, and cool down step after reaction for (a) Cu/Al_2O_3_C500R400 and (b) $CuZn/Al_2O_3$_C500R400.

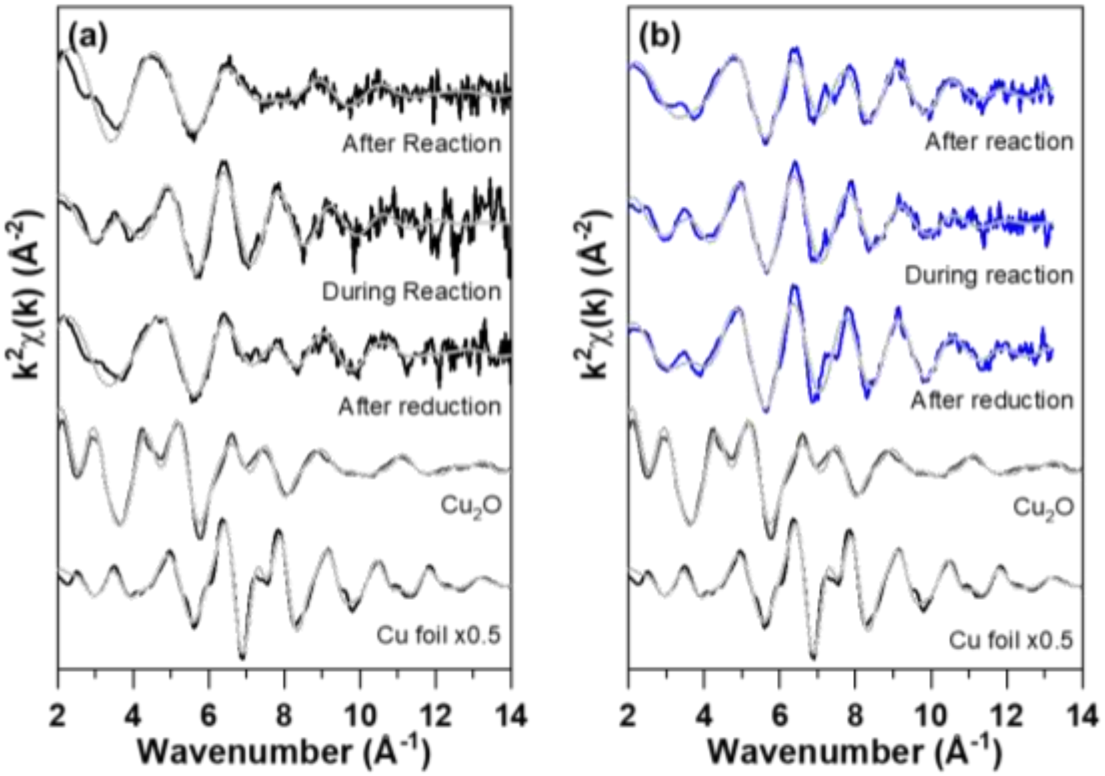

Figure S12. EXAFS oscillations in k-space at Cu K-edge for (a) Cu/Al_2O_3_C500 and (b) $CuZn/Al_2O_3$_C500 after reduction, during reaction, and after reaction. The standards Cu foil and Cu_2O CuO were added for reference.

In an attempt to evaluate the influence of water and/or hydroxyl groups on the support surface effects to the Cu speciation, we performed additional *in situ* XAFS experiments. This time, the gas was bubbled through a water vessel heated to 75 °C to enrich the flow with steam. The experimental sequence is shown in Figure S13: an initial step under standard reducing conditions (referred to as dry reduction), followed by diversion of the gas flow through the bubbler (wet reduction). Long XAFS scans were collected at four key stages: the initial state, after 15 min under dry reduction, after 15 min under wet reduction, and finally at room temperature following all treatments.

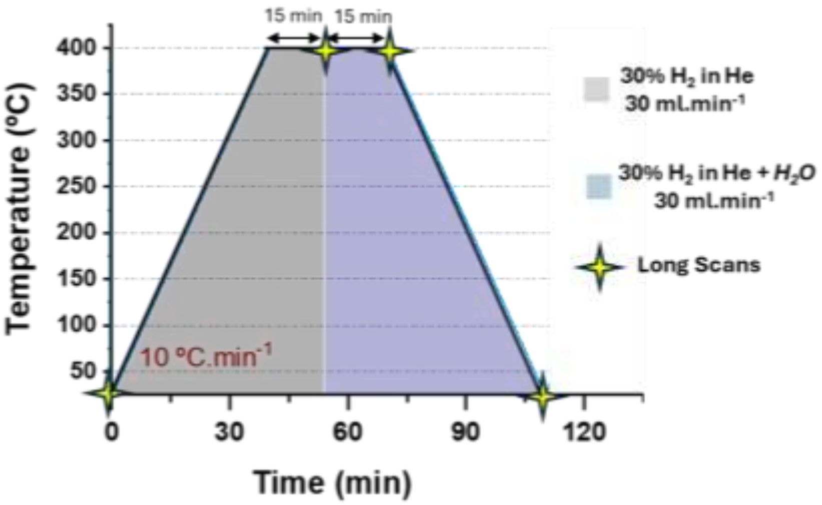

Figure S13. Illustration of the heating profile used during *in situ* XAFS experiments for studying the influence of water vapor in Cu speciation.

The results for Cu/Al_2O_3_C500 are shown in Figure S14. The dry reduction dataset was reproduced here for comparison. From the XANES data (Figure S14a), the sample reduced in the presence of steam, Cu/Al_2O_3_C500WR400_rt, exhibited a higher oxidation state, as evidenced by the increased white line (WL) intensity compared to Cu/ Al_2O_3_C500R400_rt. In the EXAFS region (Figure S14b), dry reduction led to the coexistence of Cu^0 and Cu_xO_y species, while wet reduction resulted in a more oxidized Cu phase, with little to no evidence of Cu^0. This trend was reflected in the coordination numbers (Table S5): the Cu–O CN increased from 1.7 ± 0.3 to 2.0 ± 0.3, while the Cu–Cu CN decreased from 2.2 ± 0.7 to undetectable (no successful fitting could be performed).

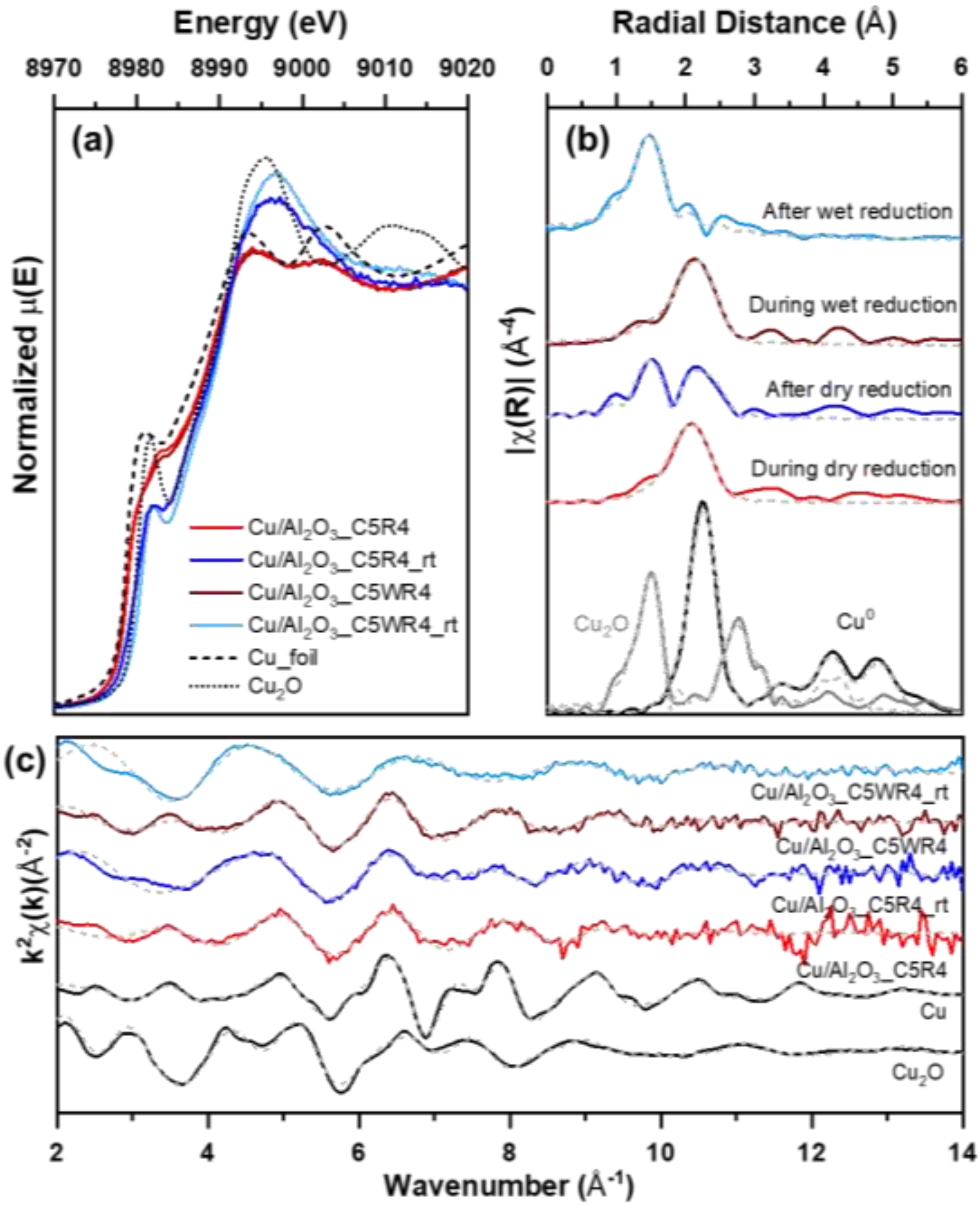

Figure S14. *In situ* XAFS spectra at Cu K-edge for Cu/Al_2O_3_C500 in the (a) XANES region, (b) EXAFS region, and (c) k plotting, taken under dry reduction at high temperature (400 ºC, Cu/Al_2O_3_C5R4) or room temperature (Cu/Al_2O_3_C5R4_rt), and wet reduction at high (Cu/Al_2O_3_C5WR4) and room temperatures (Cu/Al_2O_3_C5WR4_rt). Cu foil and Cu_2O were added for reference, and dotted light grey lines represent the best fit (typical k range: 3-11).

The dataset for CuZn/ Al_2O_3_C500 is shown in Figure S15. A similar trend was observed. In the XANES region (Figure S15a), a notable increase in WL intensity was seen for CuZn/Al_2O_3_C500WR400_rt relative to CuZn/Al_2O_3_C500R400_rt, indicating higher oxidation under wet reduction. Quantitative values extracted from the EXAFS fitting (Figure

S15b, Table S5) confirmed this: the Cu–O CN increased from 0.9 ± 0.1 to 2.1 ± 0.4 when transitioning from dry to wet reduction conditions, while the Cu–Cu CN decreased from 5.2 ± 0.3 to undetectable. Taken together, the data for both Cu and CuZn samples suggest that the presence of Zn in a steam-rich environment promotes a more extensive oxidation of the copper phase.

Table S5. *EXAFS k^2-weighting fitting parameters for Cu/Al_2O_3_C500R400 and CuZn/ Al_2O_3_C500R400 (named only as Cu and CuZn, respectively) at Cu K-edge during reduction (named with an "R") or wet reduction (named with "WR") measured either at high temperature or room temperature (RT). Cu foil and Cu_2O standards were also included.*

	Cu_foil	Cu_2O	C500R400		C500R400_RT		C500WR400		C500WR400_RT	
			Cu	CuZn	Cu	CuZn	Cu	CuZn	Cu	CuZn
**CN M-O**	-	2	0.4 (2)	0.4 (2)	1.7 (3)	0.9 (1)	0.4 (4)	-	2.0 (3)	2.1 (4)
**R M-O (Å)**	-	1.85 (1)	1.87 (3)	1.88 (2)	1.89 (1)	1.85 (1)	1.88 (4)	-	1.88 (2)	1.89 (1)
**σ^2 M-O (Å^2)**	-	0.003 (1)	0.01 (1)	0.002 (7)	0.004 (2)	0.003 (1)	0.01 (2)	-	0.004 (2)	0.005 (2)
**CN Cu-Cu**	12	12	5.6 (8)	5.9 (9)	2.2 (7)	5.2 (3)	6.0 (8)	6 (1)	-	-
**R Cu-Cu (Å)**	2.54	2.99 (1)	2.50 (1)	2.50 (1)	2.54 (1)	2.52 (2)	2.50 (1)	2.48 (1)	-	-
**σ^2 Cu-Cu (Å^2)**	0.009	0.014 (1)	0.016 (2)	0.015 (2)	0.010 (2)	0.010 (1)	0.016 (1)	0.016 (1)	-	-
**R-fator**	0.02	0.02	0.007	0.004	0.011	0.002	0.008	0.02	0.02	0.01
**Reduced χ^2**	614	343	8	7	5	8	9	9	61	40
**K (Å^{-1})**	3 - 16	2 - 16	3 – 10	3 – 10	3 – 11	3 – 11	3 – 11	3 – 11	3 – 11	3 – 11
**R (Å)**	1.3 – 5.2	1.1 – 4.2	1.1 – 2.9	1.1 – 2.9	1.1 – 2.8	1.1 – 2.8	1.1 – 2.9	1.1 – 2.6	1.1 – 2.3	1.1 – 2.3

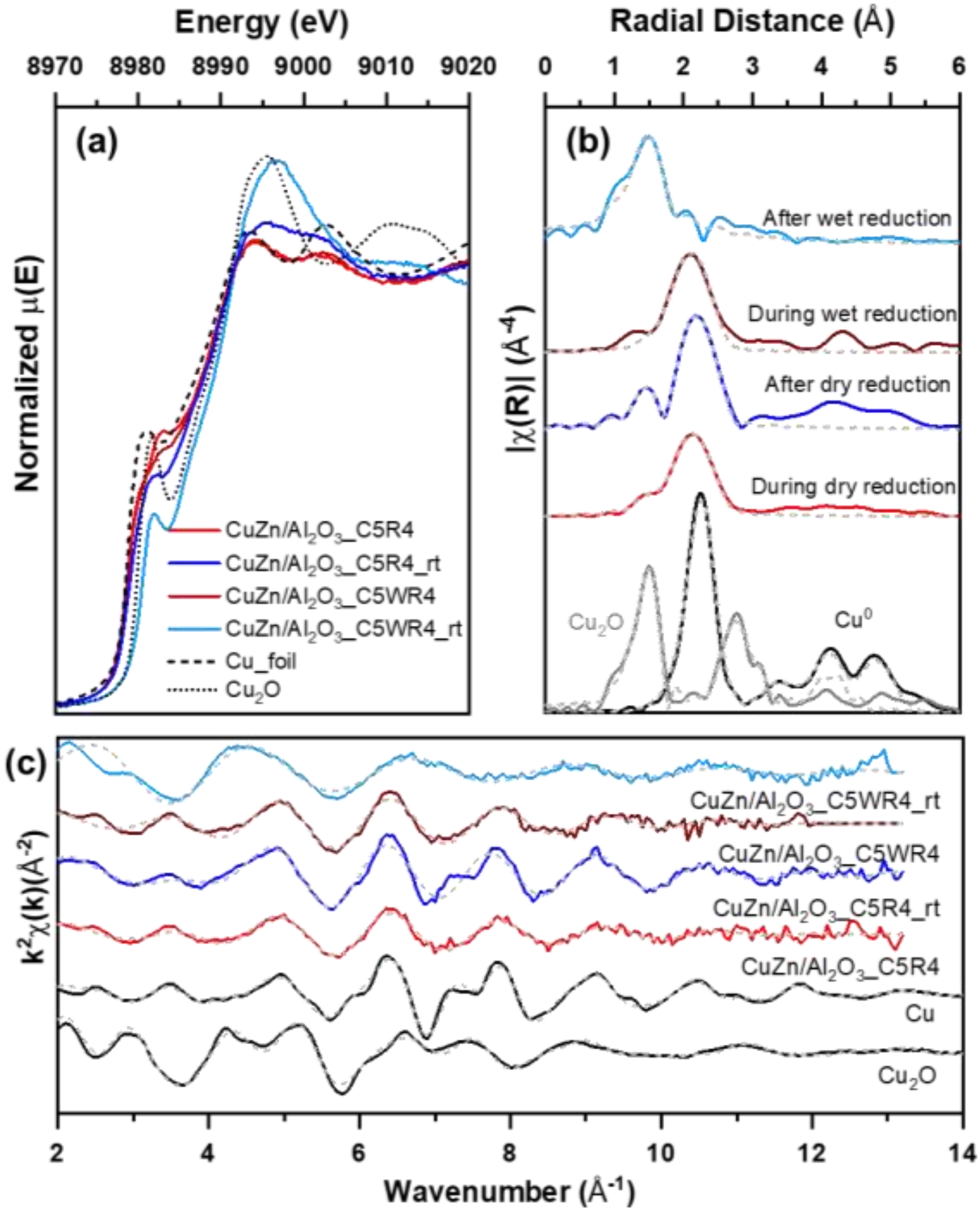

Figure S15. *In situ* XAFS spectra at Cu K-edge for CuZn/Al_2O_3_C500 in the (a) XANES region, (b) EXAFS region, and (c) k plotting, taken under dry reduction at high or room temperatures (labeled as CuZn/Al_2O_3_C5R4 and CuZn/Al_2O_3_C5R4_rt, respectively), and wet reduction at high and room temperatures (labeled as CuZn/Al_2O_3_C5WR4 and CuZn/Al_2O_3_C5WR4_rt, respectively). Cu foil and Cu_2O were added for reference, and dotted light grey lines represent the best fit (typical k range: 3-11).

5. *In situ* CO DRIFTS

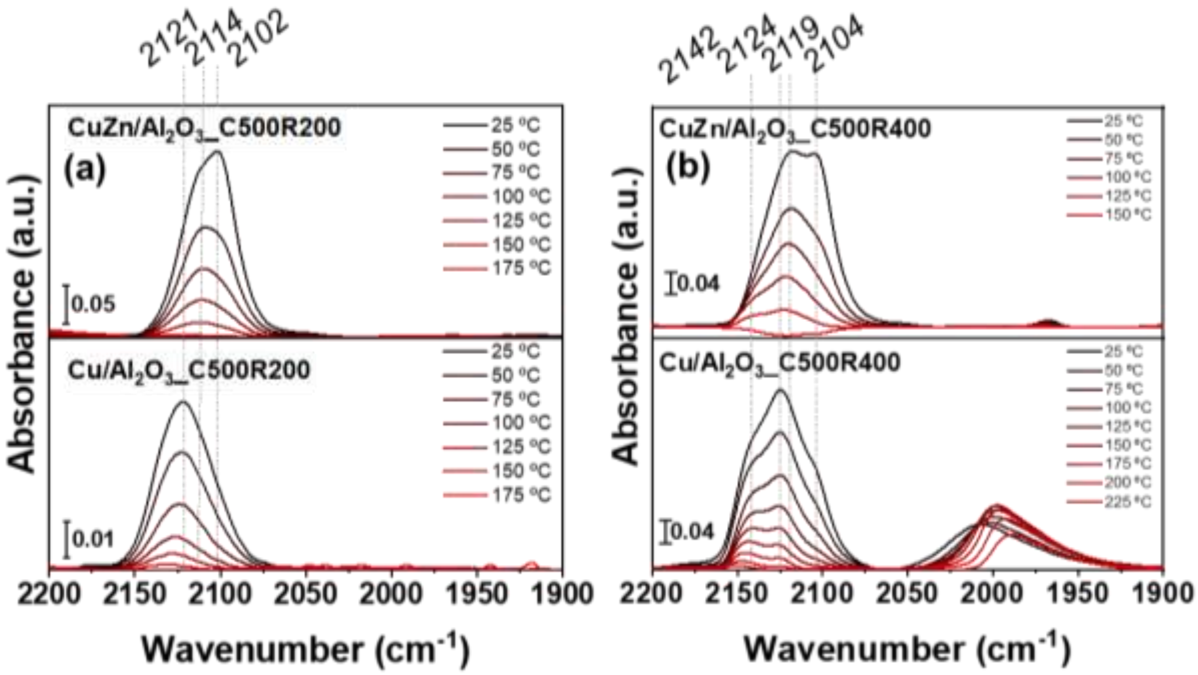

Figure S16. *In situ* CO DRIFTS Temperature Programmed Desorption for (a) Cu/Al$_2$O$_3$_C500R200 and CuZn/Al$_2$O$_3$_C500R200; (b) Cu/Al$_2$O$_3$_C500R400 and CuZn/Al$_2$O$_3$_C500R400.

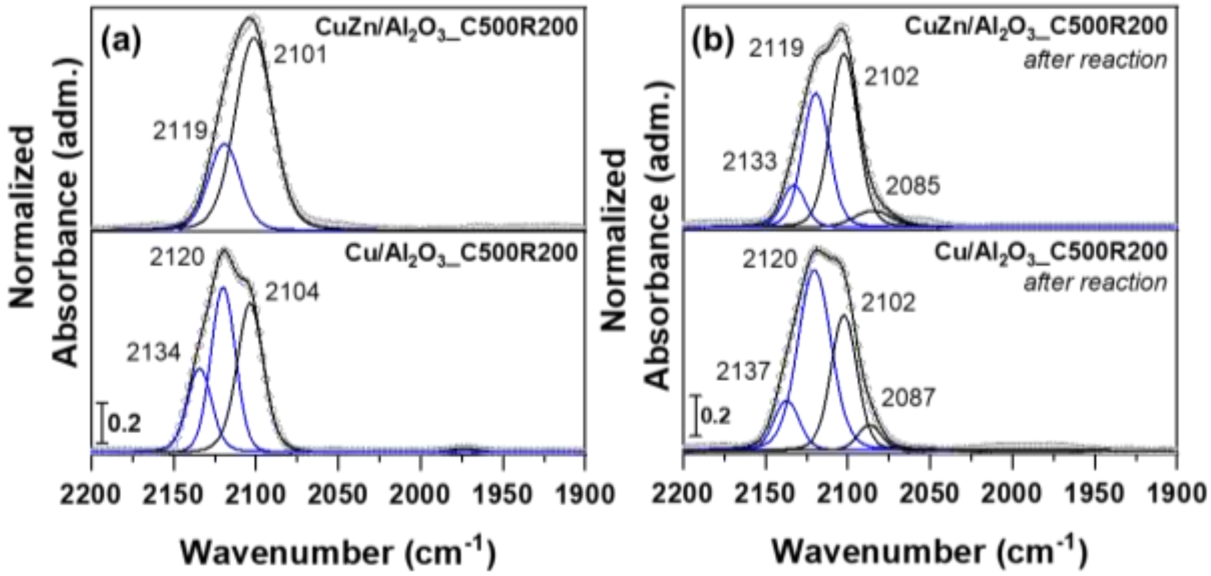

Figure S17. *In situ* CO DRIFTS for Cu/Al$_2$O$_3$_C500R200 and CuZn/Al$_2$O$_3$_C500R200 (a) after reduction; (b) materials reduced at 200 ºC after reaction.

6. *In situ* Diffuse Reflectance UV-Vis

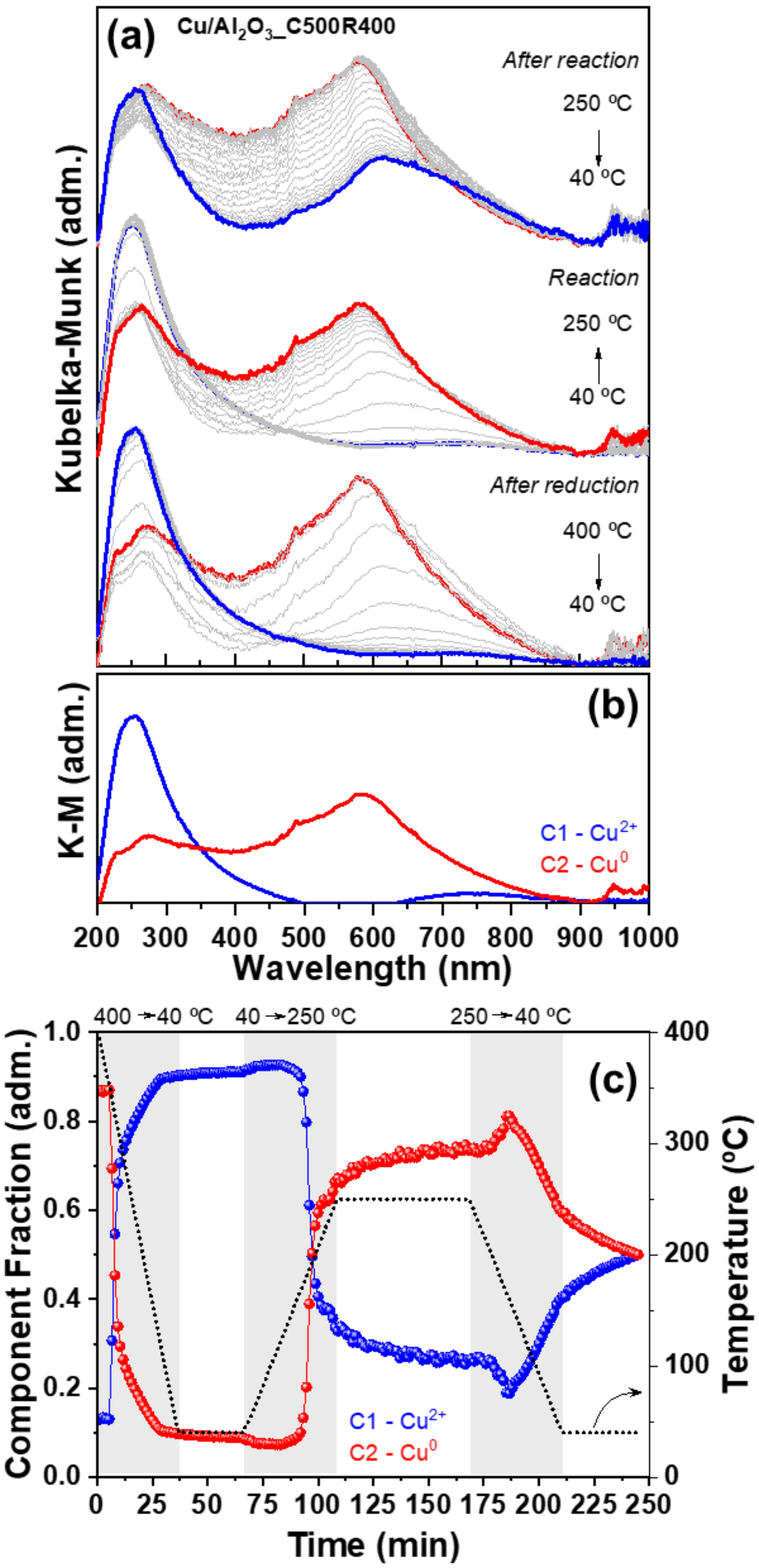

Figure S18. *In situ* DR-UV-Vis during the cooling step after reduction, heating under the reaction atmosphere, and cooling after reaction for (a) Cu/Al_2O_3_C500R400; (b) MCR-ALS components C1 (Cu^{2+}) and C2 (Cu^0); (c) MCR-ALS temperature profile.

7. Diffuse Reflectance Infrared Fourier Transform Spectroscopy (DRIFTS)

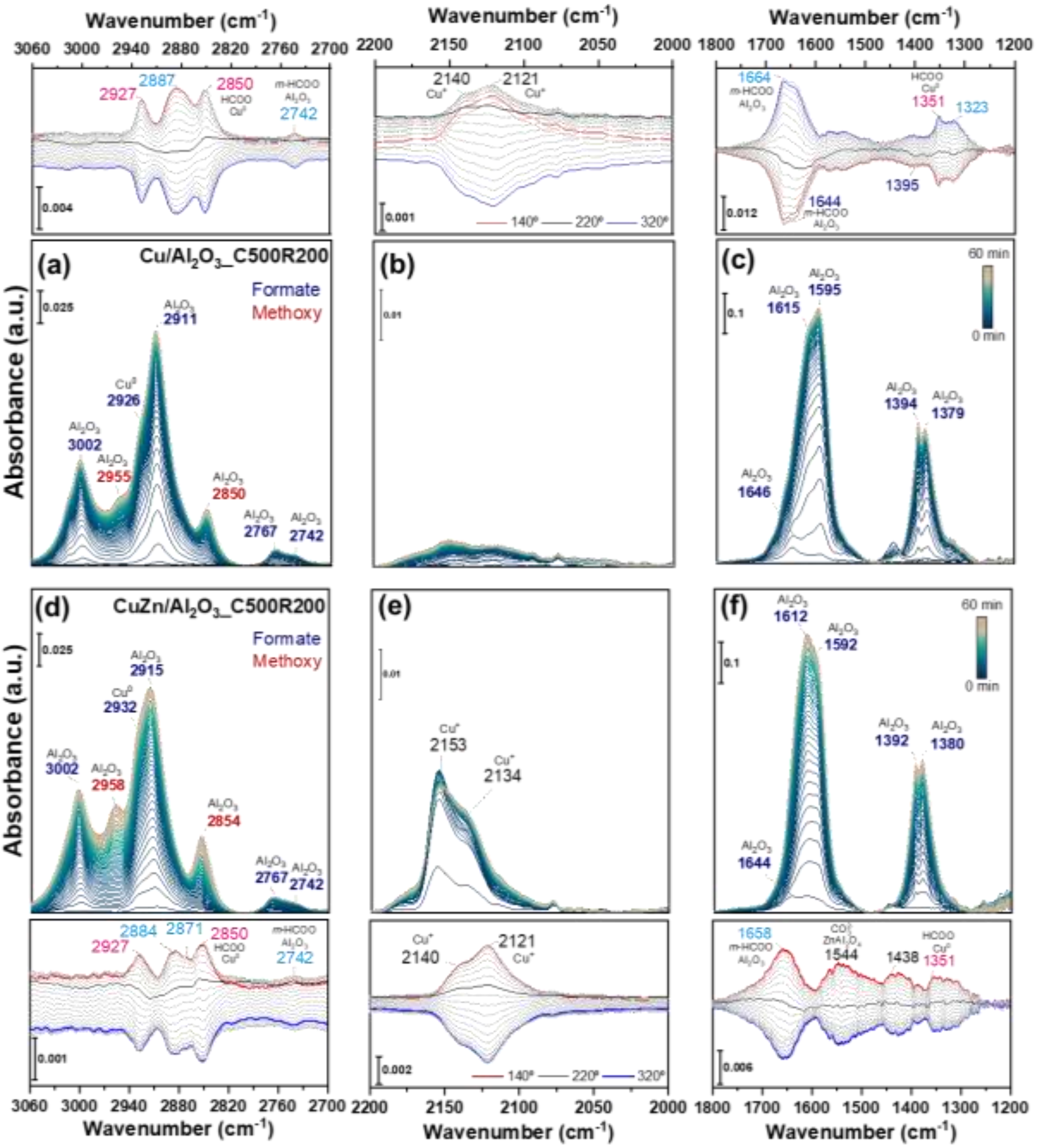

Figure S19. *In situ* reactional DRIFTS spectra as a function of time for (a-c) Cu/Al_2O_3_C500R200; (d-f) CuZn/Al_2O_3_C500R200. The experimental conditions were 1:3 CO_2:H_2, 250 °C, 1 bar, 1 h.

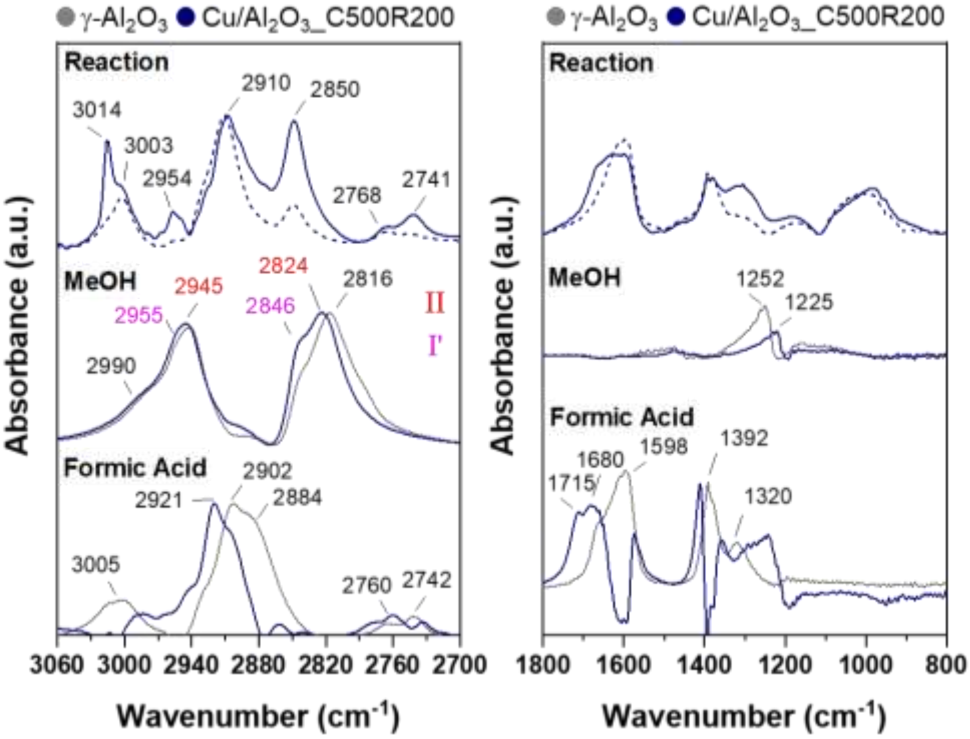

Figure S20. DRIFTS spectra for controlled chemisorption of Formic acid and Methanol at the bare γ-Al_2O_3 support and Cu/Al_2O_3_C500R200 in the (a) 3060 to 2700 cm^{-1} region; and (b) 1800 to 1200 cm^{-1} region. Steady state spectrum for Cu/Al_2O_3_C500R200 at low pressure (dotted line) and high pressure (full line) CO_2 hydrogenation is shown for reference (denoted as "reaction"). Experimental conditions for controlled chemisorption: 10 µl of formic acid or methanol, Ar 30 ml.min^{-1}, 130 ºC, 1 bar.

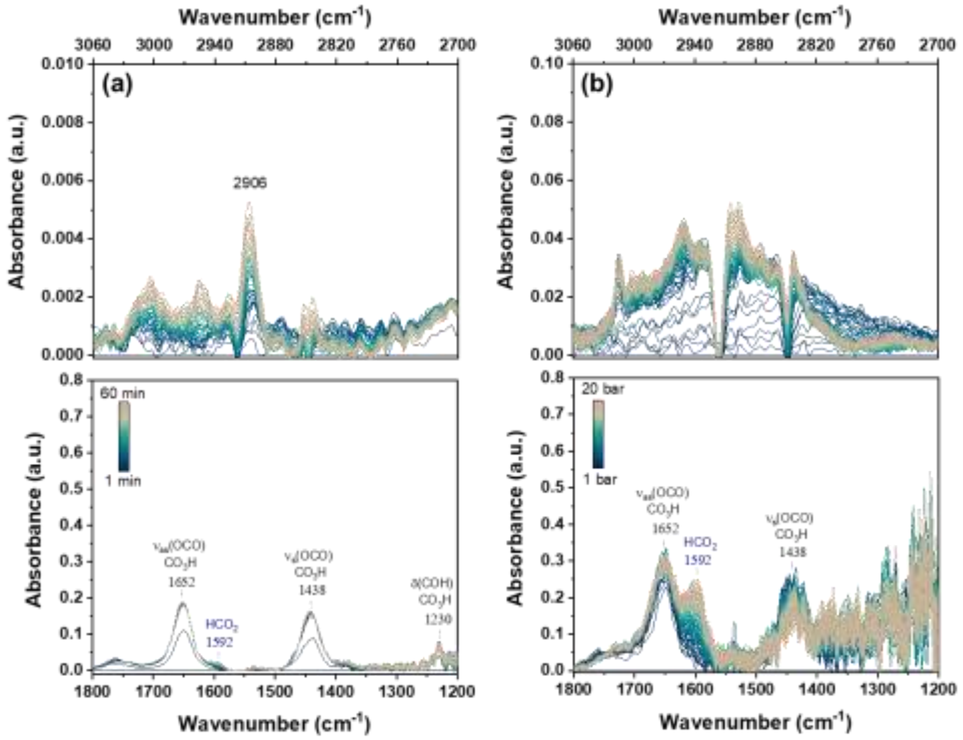

Figure S21. *In situ* and *operando* reactional DRIFTS as a function of time for the bare γ-Al_2O_3 support (a) at ambient pressure, as a function of time; and (b) from ambient pressure to 20 bar. Experimental conditions: 1:3 CO_2:H_2, 30 ml.min^{-1}, 250 ºC, 1 or 20 bar.

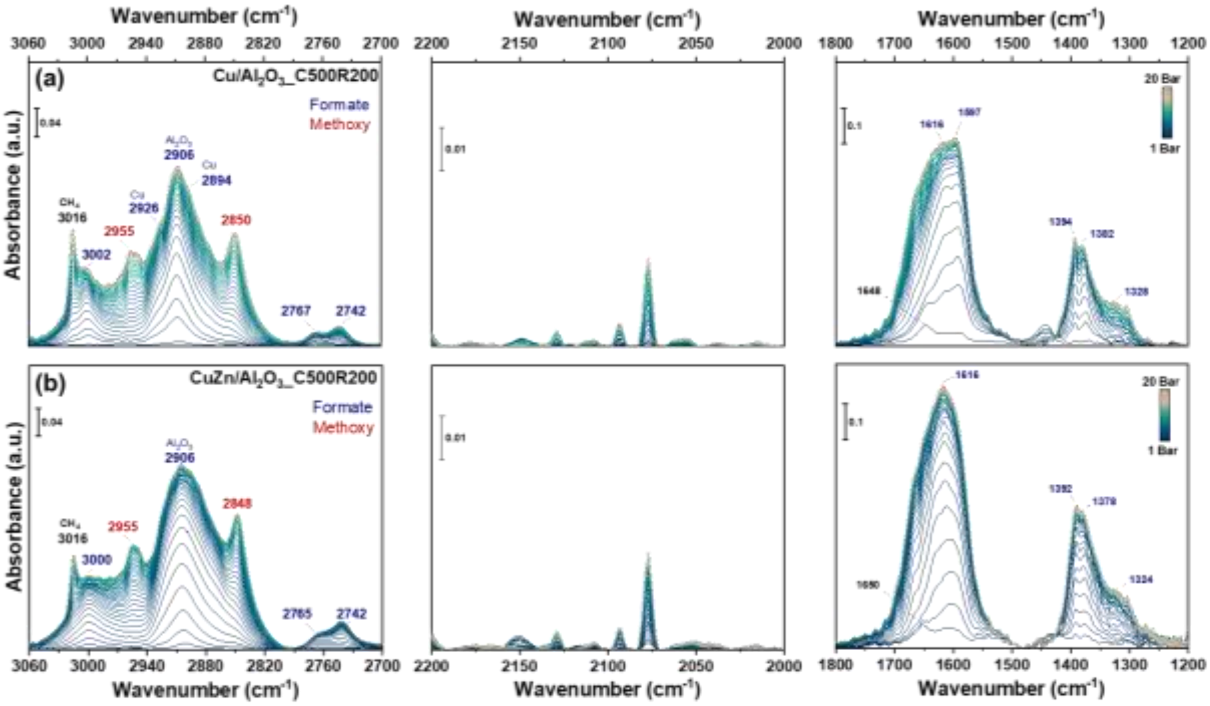

Figure S22. *Operando* reactional DRIFTS as a function of pressure for (a) Cu/Al_2O_3_C500R200; (b) CuZn/Al_2O_3_C500R200. Experimental conditions: 1:3 CO_2:H_2, 30 ml.min^{-1}, 250 ºC, from 1 bar to 20 bar.

Figure S23 illustrates the gradual increase in baseline and background contributions with pressure, the inset plots highlight the influence in the CO region. The CO bands were weaker than other surface intermediates and thus were especially susceptible to baseline fluctuations and signal-to-noise deterioration under high-pressure conditions. As a result, even small increases in background or gas-phase interference could mask these weaker CO features without implying their actual disappearance from the surface.

At higher pressures, the gas-phase absorption bands become stronger, multiple scattering within the cell increases, and the diffuse reflectance path changes due to changes in gas density. All these factors contribute to a higher background and more pronounced baseline fluctuations in the spectra. These details and cautions needed for reliable DRIFTS measurements are covered in comprehensive reviews by F.C. Meunier[1] and Francisco Zaera[2].

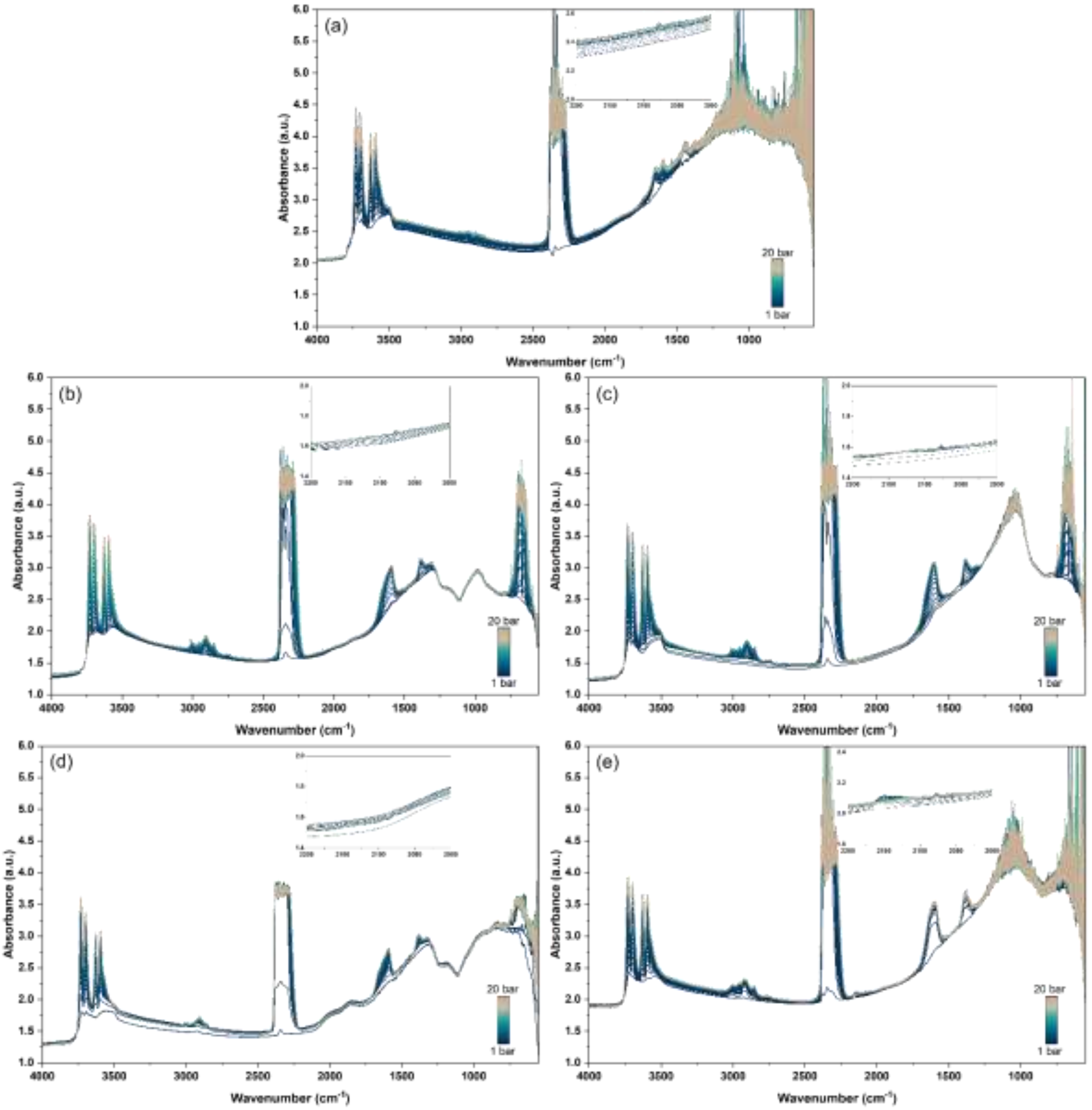

Figure S23. *Operando* reactional DRIFTS spectra (without baseline correction) as a function of pressure for (a) γ-Al_2O_3 support; (b) Cu/Al_2O_3_C500R200; (b) CuZn/Al_2O_3_C500R200; (d) Cu/Al_2O_3_C500R400; and (e) CuZn/Al_2O_3_C500R400. Inset graphs highlight the CO stretching region. Experimental conditions: 1:3 CO_2:H_2, 30 ml.min^{-1}, 250 ºC, from 1 bar to 20 bar.

8. Kinetic Insights from ME-PSD-DRIFTS

In Modulation Excitation combined with Phase-Sensitive Detection (ME-PSD), we apply a periodic stimulus (such as gas switching) to the system and observe how different chemical species respond in time. Each species may respond more or less quickly, depending on its chemical kinetics. As discussed in the manuscript, the PSD demodulation can be done following Equation 1.

$$I(\varphi^{PSD}) = \frac{2}{T}\int_0^T I(t)\sin(k\omega t + \varphi^{PSD})\,dt \qquad \text{Equation 1}$$

where $I(\varphi^{PSD})$ is the PSD data signal (absorbance) intensity, T is the modulation period, I(t) the intensity at time t, k is the demodulation index, t is the time, and φ^{PSD} is the phase angle.

In this sense, instead of directly plotting time-domain responses, ME-PSD transforms the data into the frequency domain by projecting the response signal onto a sinusoidal reference function at the same frequency as the modulation. To do this, we sweep through different demodulation phase angles (φ^{PSD}). At each φ^{PSD}, we mathematically extract how well the system's response "matches" a reference sine wave that is shifted by that angle. When this reference waveform is aligned with the system's real signal, the projection yields a large positive value — this is where the peak appears on the φ^{PSD}.

A fast species responds almost immediately to the modulation. Its signal is nearly in phase with the input. A slow species responds with a delay, its signal is lagging behind the modulation. However, in PSD, we are not measuring the delay directly. Instead, we are rotating the reference waveform to find where the overlap is strongest. This overlap becomes maximal when the demodulation phase φ^{PSD} shifts the reference waveform in such a way that it aligns with the delayed response of the system — in other words, when the φ^{PSD} value compensates for the system's internal phase lag, allowing maximum signal extraction. That is, the demodulation angle acts like a "mirror" that reveals the system's internal phase behavior, as shown in Figure S23 for an example in which two signals with delays of 30° and 120° relative to the modulation stimulus are considered.

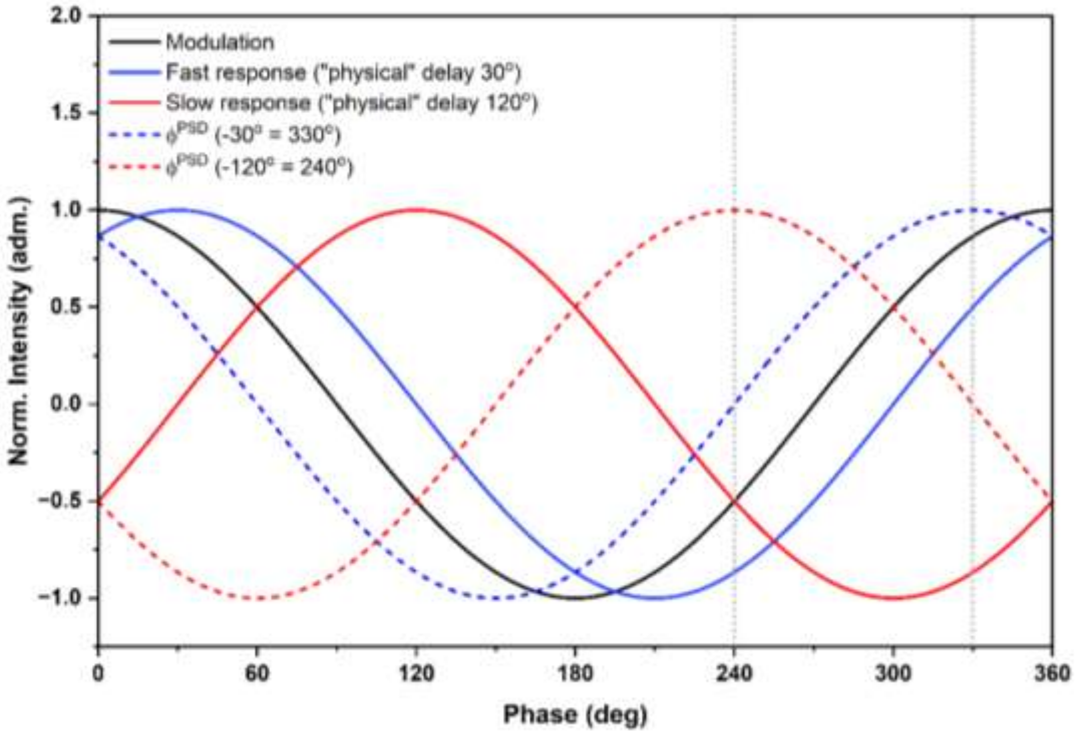

Figure S24. Relationship among the modulation stimulus with the phase response from a fast and slow responding species. Physical delay relates directly to the intermediate response, while φ^{PSD} is the phase angle needed in the PSD equation to cancel out the physical delay and have the maximum superposition between the time function and the demodulating reference sine wave. The grey lines show where the φ^{PSD} peaks in a phase plot such as Figure S24.

In this sense:

a.) A **faster species** has a **smaller actual lag** relative to the modulation. But because φ^{PSD} is chosen to cancel that lag, the **peak ends up at a larger** φ^{PSD}

b.) A **slower species**, with a larger lag, gets cancelled by a **smaller** φ^{PSD}, so its peak appears earlier.

In this way, **there is an inverse relationship** between the actual physical lag of a species and the φ^{PSD} value at which its demodulated signal peaks. The PSD method inverts the lag through this cancellation mechanism

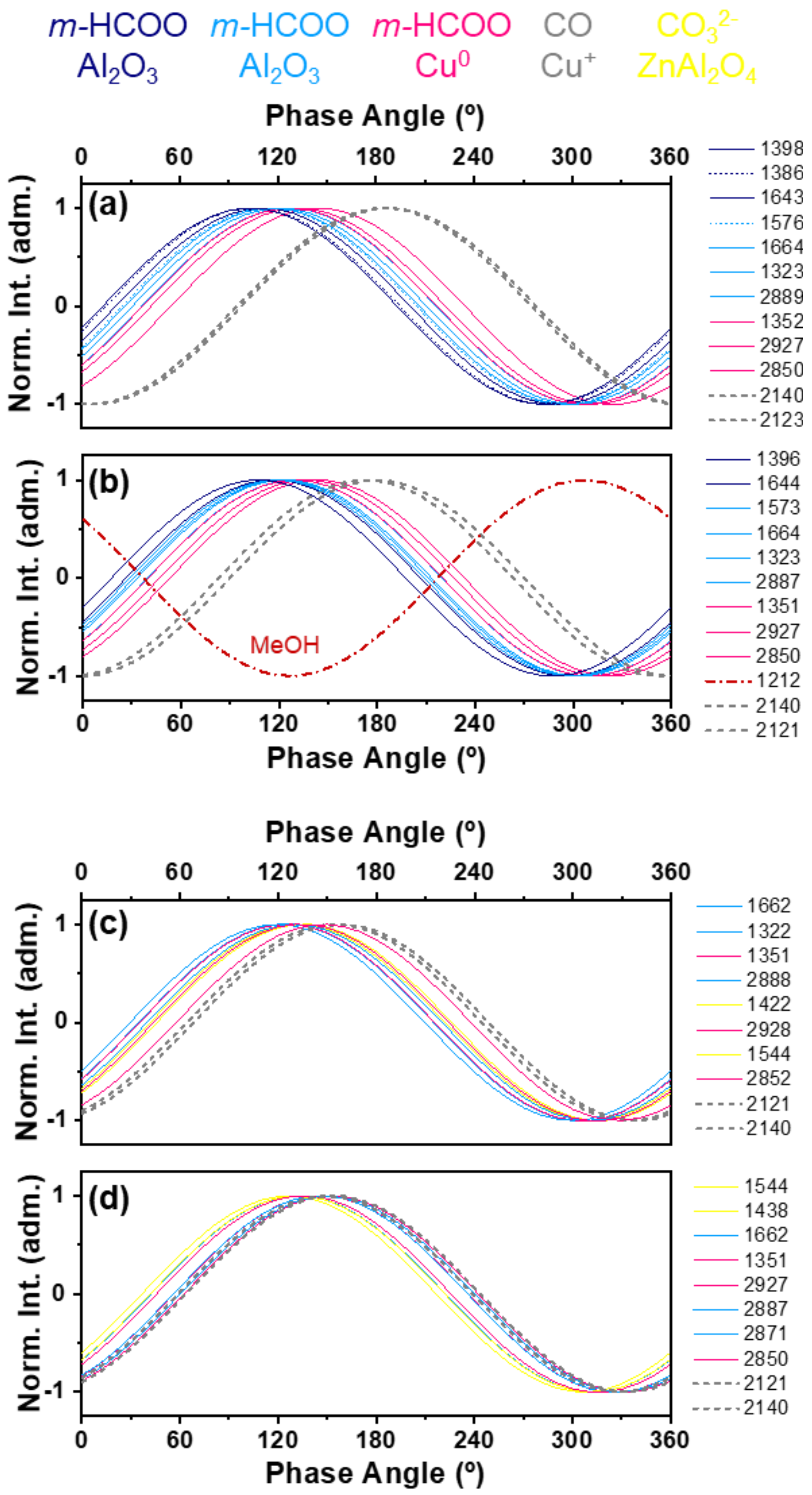

Figure S25. Normalized intensity of the bands observed in ME-PSD-DRIFTS as a function of the phase angle for (a) Cu/Al_2O_3_C500R400, (b) Cu/Al_2O_3_C500R200, (c) $CuZn/Al_2O_3$_C500R400, and (d) $CuZn/Al_2O_3$_C500R200. Highlighted in dark blue *m*-HCOO at 1644 cm^{-1}, light blue *m*-HCOO at 1664 cm^{-1}, pink HCOO-Cu^0, gray CO on Cu^+, and yellow CO_3^{2-} on $ZnAl_2O_4$.

REFERENCES

1. Meunier, F. C. Pitfalls and Benefits of in Situ and Operando Diffuse Reflectance FT-IR Spectroscopy (DRIFTS) Applied to Catalytic Reactions. *Reaction Chemistry & Engineering* **2016**, *1* (2), 134–141. https://doi.org/10.1039/c5re00018a.
2. Zaera, F. New Advances in the Use of Infrared Absorption Spectroscopy for the Characterization of Heterogeneous Catalytic Reactions. *Chem. Soc. Rev.* **2014**, *43* (22), 7624–7663. https://doi.org/10.1039/c3cs60374a.
